# CD44 restricts EGFR mobility to polarize cytoskeletal signalling modules driving bleb-based migration

**DOI:** 10.1038/s41556-026-01981-1

**Published:** 2026-07-06

**Authors:** Ankita Jha, Ankit Chandra, Payam E. Farahani, Jared E. Toettcher, Ana M. Pasapera, Jason M. Haugh, Clare M. Waterman

**Affiliations:** 1 Cell and Developmental Biology Center, National Heart Lung and Blood Institute, National Institutes of Health, Bethesda, MD USA; 2 Department of Chemical and Biomolecular Engineering, North Carolina State University, Raleigh, NC USA; 3 Department of Chemical and Biological Engineering, Princeton University, Princeton, NJ USA; 4 InduPro, Seattle, WA USA; 5 Department of Molecular Biology, Princeton University, Princeton, NJ USA; 6 Omenn-Darling Bioengineering Institute, Princeton University, Princeton, NJ USA

**Keywords:** Amoeboid migration, Membrane biophysics, Metastasis

## Abstract

Cells under high confinement migrate efficiently in low-adhesion environments by forming stable, polarized, hydrostatic pressure-driven leader blebs. Here we investigated the basis of polarized bleb morphology in metastatic melanoma cells migrating under low-adhesive and highly confined microenvironments. Using high-resolution live imaging, molecular perturbations and biosensors, we show that EGF signalling through PI3K stabilizes and maintains polarized leader blebs. EGFR and PI3K activities form a gradient within leader blebs that decreases from rear to front, promoting phosphatidylinositol 3,4,5-trisphosphate and Rac1-GTP accumulation at the bleb rear, whereas phosphatidylinositol 4,5-bisphosphate and RhoA-GTP concentrate at the bleb tip, the inverse of the organization observed in integrin-dependent mesenchymal migration. Optogenetic disruption of this gradient triggers bleb retraction, underscoring its functional importance. Mathematical modelling and experiments identified a mechanism whereby during bleb initiation, CD44 and ERM proteins restrict EGFR mobility within a membrane-apposed cortical actin meshwork at the bleb rear, establishing the EGFR–PI3K–Rac gradient. Together, these findings define the biophysical and molecular mechanisms that underlie polarity in bleb-based migration and highlight how alternative spatial organization of signalling modules supports distinct migration modes in different microenvironments.

## Abstract (web summary)

Jha et al. show that the transmembrane protein CD44 restricts EGFR mobility to maintain an EGFR–PI3K–Rac gradient during bleb-based cell migration.

## Main

Cancer cell migration has a crucial role in invasion and metastasis, which is often fatal^1–3^. During metastasis, tumour cells migrate through various tissue and extracellular matrix (ECM) geometries, including confined spaces, to spread to distant sites^4,5^. For cells to migrate, they must establish spatial polarity to create an actomyosin architecture that generates protrusion in the direction of movement, adhesion, traction forces to move relative to the surroundings, and retraction of the cell rear to make forward progress^6–8^. Tumour cells can utilize different modes of migration, influenced by their interactions with the local microenvironment^9^. Depending on the availability of adhesion ligands, cancer cells can dynamically switch between integrin-mediated mesenchymal migration and adhesion-independent amoeboid migration^9–12^. When ECM ligand is unavailable or integrin signalling is suppressed, metastatic tumour cells switch to amoeboid migration, in which they adopt a bleb-driven mode of protrusion^13–15^. Blebbing is associated with increased Rho activity and is particularly pronounced in confined spaces, where high Rho-mediated contractility and elevated hydrostatic pressure from the confinement drive formation of large blebs that facilitate persistent migration^13,16–21^. Despite its prevalence in metastatic cancer, the mechanisms that govern bleb-based migration in confined environments remain unclear.

Although establishment of cell polarity and its maintenance during mesenchymal migration are well understood^6,7,22^, how these are achieved during bleb-based migration remain largely unknown. Generation of polarity requires symmetry breaking, which can occur in response to asymmetric cues, including diffusible or substrate-bound molecules and physical cues^22–24^ that establishes and maintains cytoskeletal asymmetry. For instance, in mesenchymal migration, growth factor gradients result in receptor tyrosine kinase (RTK) and G-protein-coupled receptor activation at the cell front^25^ triggering self-amplifying signalling through PI3K, resulting in local accumulation of phosphatidylinositol 3,4,5-trisphosphate (PtdIns(3,4,5)P_3_) and Rac or Cdc42 activation to promote actin-based protrusions at the cell front^7^. Simultaneously, phosphatidylinositol 4,5-bisphosphate (PtdIns(4,5)P_2_) and Rho GTPase activity at the cell rear drive formin-based actin bundle formation and myosin II activation, generating traction and rear retraction^7,26^. By contrast, cells migrating via bleb formation in high confinement undergo spontaneous symmetry breaking^20,21,24,27^, which has been shown to occur in other systems via cortical cytoskeletal instabilities^27^. In bleb-based migration, formin-based actin bundles form just proximal to the bleb tip^15,28^ and dense actin networks, along with myosin II activation, dominate the rear of the bleb^17,20,28^. This asymmetry in polymerization and contraction drives cortical actomyosin flow within the bleb, which when coupled to transmembrane proteins, generate friction on the tightly confined microenvironment to propel the cell forward^20,21,28^. However, how polarized signalling is established to maintain and reinforce cytoskeletal polarity in the absence of directional cues in bleb-based migration remains unknown^15^.

Spatial regulation of the cortical cytoskeleton arises from asymmetries in plasma membrane receptors and their downstream signalling through multiple mechanisms. One process to spatially localize cytoskeletal signalling is by advective movement of signalling proteins along with lipid or cortical flows driven by membrane tension gradients or direct or indirect binding to flowing cortical actomyosin^21,29–31^, as occurs in establishment of body-axis polarity in the early *Caenorhabditis elegans* embryo^32^. Alternatively, regions of specific plasma membrane composition can be formed by local addition or removal of surface proteins by spatial regulation of endo- and exocytosis, as occurs in *Drosophila melanogaster* ovary, where RTK signalling is spatially restricted in migrating border cells^33,34^. In addition, spatial restriction of receptor lateral mobility within the plasma membrane, known as ‘corralling’ by transmembrane ‘picket proteins’ can generate and maintain domains of composition-specific plasma membrane^35^. For instance, during phagocytosis, large transmembrane proteins, such as CD44 act as pickets to limit the diffusion of Fcγ receptors by corralling at the rear of polarized macrophages, leaving the receptors more mobile at the leading edge^36^. Whether these mechanisms govern establishment of stable polarity in bleb-based migration is yet to be determined.

In this study, we aimed to identify the mechanisms that enable the persistent cell polarity that drives bleb-based migration of metastatic cancer cells in confined, low-adhesive environments. We previously showed that leader-bleb-based migration in metastatic melanoma cells under confinement is driven by Erk signalling^19^, which is linked to the EGF–EGFR–Ras–Raf pathways. We thus hypothesized that the polarized distribution of growth factor RTKs and their downstream signalling components, such as phosphoinositides and Rho GTPases^37–39^, are required for maintaining polarity and driving persistent migration in metastatic melanoma cells under confinement. Using confinement assays, live cell imaging and optogenetics we show that bleb-based migration repurposes polarity signalling modules of mesenchymal migration, but with inverted spatial organization. Whereas EGFR–PI3K–Rac signalling localizes to the front of mesenchymal cells, we find this signalling must localize to the bleb rear to maintain bleb stability and persistent directional migration. We further reveal that this rear-to-front EGFR gradient is maintained by restricting EGFR mobility mediated by CD44 and the cortical actin linker protein ezrin. These findings reveal a novel polarity mechanism that promotes rapid cancer cell migration under confinement that may be critical to their highly invasive behaviour.

## Results

### EGFR signalling through PI3K is required for leader-bleb stability and persistent migration under low-adhesive confinement

**Fig. 1:**
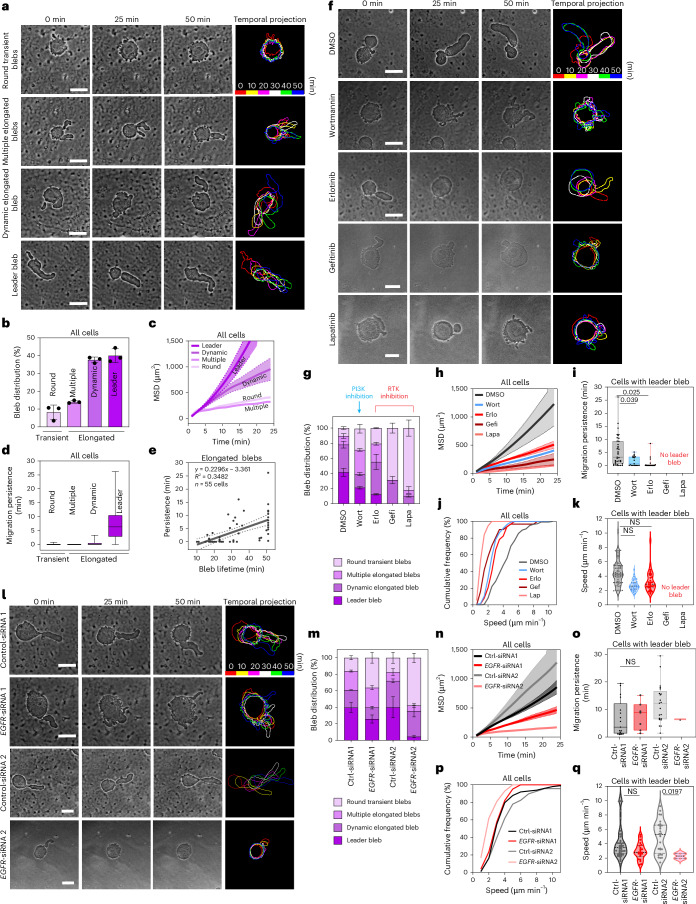
EGFR signals via PI3K to maintain leader-bleb-based migration in low-adhesive confinement. **a**,**f**,**l**, Representative time-lapse phase-contrast images (left three columns) of A375M2 cells migrating under 3-µm confinement, with temporal colour-encoded projections of cell outlines at 10-min intervals (right). Scale bars, 25 μm. **a**, Four major bleb phenotypes are observed: round transient blebs (top row), multiple elongated blebs (row 2), dynamic elongated blebs (row 3) and leader blebs (bottom row). **b**,**c**, Quantification of bleb phenotype distribution (**b**; bars show mean ± s.d.) and mean square displacement (MSD) over time (**c**; dark line shows mean, dotted lines and shaded area represent s.d.). Analyses include round transient blebs (*n* = 21), and multiple (*n* = 18), dynamic (*n* = 31) and leader blebs (*n* = 44). **d**, Box plot of migration persistence for each phenotype as in **b** (left to right: *n* = 14, 17, 25, 29). **e**, Migration persistence as a function of bleb lifetime in cells with polarized elongated blebs (*n* = 55 blebs). Linear fit with 95% confidence interval. **f**, Cells treated with DMSO, wortmannin (100 nM), erlotinib (50 nM), gefitinib (1 µM) or lapatinib (1 µM). **g**,**h**, Bleb phenotype distribution (**g**; stacked column bars) and MSD over time (**h**; mean ± s.d.) following drug treatments (drug treatments, left to right in **g**: *n* = 405, 166, 208, 32, 39). Erlo, erlotinib; Gefi, gefitinib; Lapa, lapatinib; Wort, wortmannin. **i**, Migration persistence of leader-bleb cells after DMSO (*n* = 37), wortmannin (*n* = 19, *P* = 0.039), erlotinib (*n* = 23, *P* = 0.0235), gefitinib or lapatinib (no leader blebs) treatment; two-tailed Welch’s *t*-test on *n*. **j**, Cumulative frequency distributions of migration speed after drug treatments (drug treatments, top to bottom: *n* = 145, 133, 97, 31, 38). **k**, Violin plots of migration speed of leader-bleb cells after drug treatments (*n* = 64 (DMSO), 39 (wortmannin), 30 (erlotinib), 0 (gefitinib and lapatinib)); two-tailed Welch’s *t*-test on *N*. **l**, Cells transfected with non-targeting control siRNAs (control-siRNA1 and control-siRNA2) or pooled *EGFR*-targeting siRNAs (*EGFR*-siRNA1 and *EGFR*-siRNA2). **m**,**n**, Bleb distribution (**m**; bars show mean ± s.d.) and MSD (**n**; dark line shows mean and shaded area represents s.d.) for control (ctrl)-siRNA1 (*n* = 118; MSD *n* = 84), *EGFR*-siRNA1 (*n* = 116; MSD *n* = 98), control-siRNA2 (*n* = 75; MSD *n* = 52) and *EGFR*-siRNA2 (*n* = 45; MSD *n* = 42). **o**, Migration persistence of leader blebs after siRNA; left to right: *n* = 17, 7, 21, 1; not significant, two-tailed unpaired *t*-test with Welch’s correction on *n*. **p**, Cumulative frequency distributions of migration speed after siRNA as in **l** (groups, top to bottom: *n* = 98,123, 68, 42). **q**, Violin plots of migration speed of leader-bleb cells after control-siRNA1 (*n* = 47) versus *EGFR*-siRNA1 (*n* = 22) and control-siRNA2 (*n* = 28) versus *EGFR*-siRNA2 (*n* = 22, *P* = 0.0197); two-tailed unpaired *t*-test with Welch’s correction on *N*. *n* denotes number of cells; *N* = 3 independent experiments. In box plots (**d**,**i**,**o**) the centre line is the median, box edges delineate interquartile range and whiskers extend to minimum and maximum values. *P* < 0.05 was considered significant; NS, not significant (*P* ≥ 0.05). Source data

**Extended Data Fig. 1:**
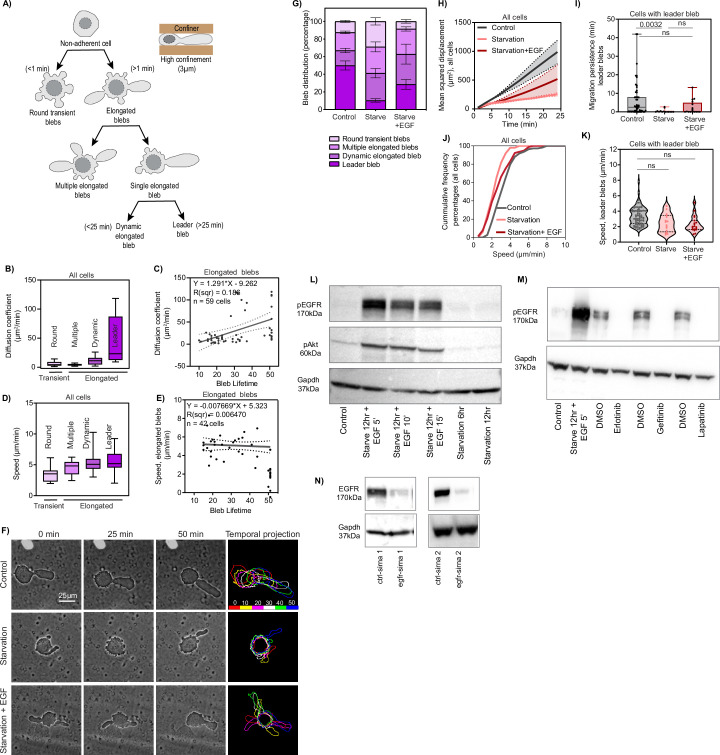
Leader bleb-based migration requires EGFR signaling. (**A**) Schematic of the confinement geometry used in this study (top right) and classification of bleb morphologies observed in A375M2 melanoma cells under high confinement and low adhesion (left). Cells form either round transient blebs or elongated blebs. Elongated blebs are classified as multiple or single; single elongated blebs are further categorized as dynamic ( < 25 min) or leader blebs ( > 25 min) based on lifetime. (**B**) Box plots of diffusion coefficients for round transient, multiple elongated, dynamic elongated, and leader bleb phenotypes, n = 14, 16, 13, and 24 cells, respectively. (**C**) Diffusion coefficient as a function of bleb lifetime in cells with polarized elongated blebs (n = 59 cells); solid line, linear fit; dotted line, 95% confidence interval. (**D**) Box plots of migration speed for bleb phenotypes as in (**B**); n = 17, 17, 31, and 35. (**E**) Average migration speed of cells with a single elongated bleb as a function of bleb lifetime (n = 55 blebs); linear fit with 95% confidence interval. (**F**) Representative time-lapse phase-contrast images of A375M2 cells under 3-µm confinement with temporal color-encoded projections at 10-min intervals. Cells were untreated (control), serum starved, or serum starved and stimulated with EGF (50 ng mL⁻^1^). (**G**,**H**) Quantification of bleb phenotype distribution (**G**) and mean-square displacement (MSD) over time (**H**; mean ± s.d.) for control (n = 112), starvation (n = 97), and starvation + EGF (n = 70). (**I**) Migration persistence of leader-bleb cells under the same conditions, n = 55, 14, and 17; p values: control vs starvation, p = 0.0032; all other comparisons, p=ns; Two-tailed unpaired t-test with Welch’s correction. (**J**) Cumulative frequency distributions of migration speed (n = 109, 77, and 49). (**K**) Violin plots of migration speed of leader-bleb cells (n = 77, 16, and 19); all comparisons p=ns. (**L**–**N**) Immunoblot analysis of A375M2 cell lysates. (**L**) Cells were untreated, serum starved for 6 or 12 h, or serum starved for 12 h and restimulated with EGF (50 ng mL⁻^1^) for 5–15 min; blots probed for pEGFR, pAkt, and GAPDH. (**M**) Cells were untreated, serum starved, or treated with EGF, DMSO, erlotinib (50 nM), gefitinib (1 µM), or lapatinib (1 µM); blots probed for pEGFR and GAPDH. (**N**) Cells transfected with control siRNA or EGFR siRNAs (two sequences) were probed for total EGFR and GAPDH. **n** denotes number of cells from N = 3 independent experiments. Box plots show median, interquartile range, and minimum–maximum values.

We first sought to determine whether the highly polarized bleb morphology of metastatic melanoma cells migrating in a confined, low-adhesive microenvironment was functionally relevant to cell migration. We performed time-lapse imaging of A375M2 human metastatic melanoma cells that were held in low-adhesive confinement to 3 μm height using a polydimethylsiloxane (PDMS) cellular confinement device, mimicking the degree of tissue confinement observed during metastasis in vivo^5^. Characterizing the relationship between cell morphology and motility showed that confinement caused blebs that were typified by distinct states of morphology and stability (Extended Data Fig. 1a). This included a minor population of cells with small transient round blebs around their peripheries with a lifetime of less than 1 min (ref. ^16^), whereas most cells displayed elongated blebs (Fig. 1a,b), as described previously^19–21,40^. Quantitative analysis showed that cells with elongated blebs exhibited a minor population in a non-polarized state with multiple elongated blebs around their peripheries, with most displaying a highly polarized morphology with a single elongated bleb (Fig. 1b). We classified cells with single elongated blebs as either dynamic blebs (lifetime <25 min) or leader blebs^19^ (lifetime >25 min; Fig. 1a,b and Supplementary Video 1). Analysis of motility parameters showed that cells exhibiting stable leader blebs migrated efficiently compared with cells exhibiting other morphologies, displaying significantly greater migratory range, as measured by a greater slope on a mean squared displacement over time plot, increased migration persistence, and a higher diffusion coefficient (Fig. 1c,d and Extended Data Fig. 1b). Indeed, plotting the lifetime of elongated blebs versus either their migration persistence or diffusion coefficient showed positive correlations (Fig. 1e and Extended Data Fig. 1c). By contrast, similar analysis of bleb lifetime versus speed showed that cells with an elongated bleb moved at a wide range of speeds, independent of bleb lifetime (Extended Data Fig. 1d,e). This is consistent with the previous studies which shows that cells move with a wide range of speed. Hence, the distribution of speed, as opposed to the mean speed, is a better metric for the effects of perturbations on changes in cell migration speed^30^. These results show that stabilization and maintenance of a polarized leader-bleb morphology is critical to efficient and persistent migration in metastatic melanoma cells in low-adhesive confinement.

As growth factor gradients are critical to cell polarization in mesenchymal migration of cancer cells^41,42^, we next probed the role of serum components and growth factors in leader-bleb polarization and stability. We found that serum starvation of confined A375M2 cells decreased formation of stable leader blebs and increased the fraction of cells with round transient blebs compared with cells confined in serum-containing medium (Extended Data Fig. 1f,g). Analysis of cell motility showed that serum starvation strongly reduced migratory range for all cells, and the few cells that exhibited leader-bleb morphology had reduced migration persistence but no difference in their speed while moving compared with leader-bleb cells in serum (Extended Data Fig. 1h–k). Thus, serum factors stabilize leader-bleb formation and persistent migration.

As EGF signalling is critical for cell migration and developing drug resistance in metastatic melanoma^43,44^, and starvation downregulates this signalling (Extended Data Fig. 1l), we reasoned that EGF could be the serum factor responsible for promoting leader-bleb-based migration in low-adhesive confinement. Notably, addition of EGF to serum-starved cells partially rescued the leader-bleb morphology (Extended Data Fig. 1f,g and Supplementary Video 2), increased the migratory range of all cells, and for leader-bleb cells, increased the migration persistence, but had no significant effect on their speed (Extended Data Fig. 1h–k). Furthermore, targeting EGFR tyrosine kinase activity with erlotinib (50 nM), gefitinib (1 µM), lapatinib (1 µM) or blocking its downstream effector PI3K with wortmannin (100 nM; Extended Data Fig. 1m), strongly reduced leader-bleb formation, cell migratory range and speed of all cells (Fig. 1f–h,j and Supplementary Video 3). The few cells that did make leader blebs under erlotinib or wortmannin treatment showed significantly reduced migration persistence, but showed no difference in migration speed compared with leader-bleb cells in DMSO control (Fig. 1i,k). Similarly, knockdown of *EGFR* expression using two independent small interfering RNAs (siRNAs) (Extended Data Fig. 1n) strongly inhibited the formation of leader blebs, cell migratory range and speed of all cells compared with non-targeting siRNAs, (Fig. 1l,m,p and Supplementary Video 4J). Cells exhibiting leader-bleb morphology, after *EGFR*-siRNA2 knockdown, reduced both migration persistence and speed (Fig. 1o,q). Thus, EGF signalling through PI3K is required for stabilizing and maintaining polarized, elongated stable leader-bleb morphology to drive persistent migration and promote migratory range under low-adhesive confinement. Furthermore, these results show that once cells establish and maintain a leader bleb, they migrate persistently and rapidly, independent of the perturbation, with the primary effect of perturbations being to inhibit formation and maintenance of leader blebs.

### EGFR, PI3K and Rac1 activities exhibit rear-to-front gradients in leader blebs

**Fig. 2:**
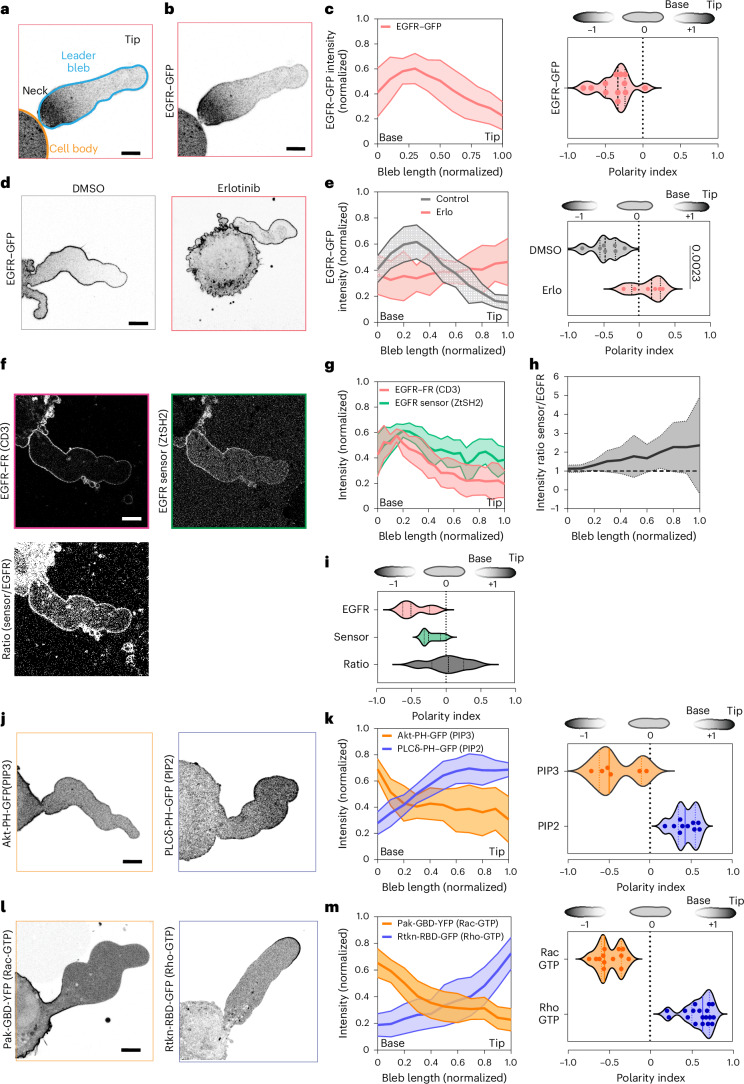
EGFR, PI3K and Rac1 activities form rear-to-front gradients in leader blebs. **a**,**b**,**d**,**f**,**j**,**l**, Super-resolution confocal images taken during confinement to 3 µm of A375M2 melanoma cells. Scale bars, 5 μm. **a**, Inverted contrast image of a cell transfected with EGFR–GFP with marked leader bleb in blue and cell body in orange. **b**, Inverted contrast image of a cell transfected with EGFR–GFP. **c**, Normalized intensity (dark line shows mean and shaded area represents s.d.) of EGFR–GFP across the normalized length of the leader bleb (left) and violin plot of EGFR–GFP polarity index (right; *n* = 12 cells). **d**, Inverted contrast images of cells transfected with EGFR–GFP and treated with DMSO (grey box) or erlotinib (50 nM, red box). **e**, Normalized intensity (dark line shows mean and shaded area represents s.d.) of EGFR–GFP across the normalized length of the leader bleb (left) and violin plot of EGFR–GFP polarity index (right) for cells treated with either DMSO (grey, *n* = 7 blebs) or erlotinib (pink, *n* = 6 blebs). *P* = 0.0023, two-tailed, Mann–Whitney test. **f**, EGFR–CD3–FR (red box, left) and ZtSH2–CLV (sensor, green box, right) and ratio image of sensor to EGFR–CD3–FR (bottom). **g**, Normalized intensity (dark line shows mean and shaded area represents s.d.) of the constructs in **f** across the normalized length of leader blebs. **h**, Normalized intensity of the ratio of the sensor to EGFR–CD3 across the normalized length of the bleb. **i**, Violin plot of polarity indices of fluorescence distribution along the leader bleb of constructs from **f** and ratio. **g**–**i**, *n* = 8 cells. **j**, Inverted contrast images of cells transfected with Akt-PH–GFP (orange box) or PLCδ-PH–GFP (purple box). **k**, Normalized intensity profile of Akt-PH–GFP (orange, *n* = 6 blebs) or PLCδ-PH–GFP (purple, *n* = 10 blebs) across the normalized length of the bleb, (dark line shows mean and shaded area represents s.d.), violin plot of polarity indices of fluorescence distribution along the leader bleb of constructs from **j**. **l**, Inverted contrast images of cells transfected with Pak-GBD–YFP or Rtkn-RBD–GFP. **m**, Normalized intensity profile of Pak-GBD–YFP (*n* = 13 blebs) or Rtkn-RBD–GFP (*n* = 18 blebs) across normalized length of the bleb (left; dark line shows mean and shaded area represents s.d.) and violin plot of polarity indices of fluorescence distribution along the leader bleb of constructs from **l** (right). Source data

We next focused on understanding how EGF signalling stabilizes the polarized morphology of elongated blebs in melanoma cells under low-adhesive confinement. Super-resolution confocal imaging of EGFR–GFP revealed a strong gradient along the plasma membrane of leader blebs, with low concentration at the distal bleb tip that increased to a peak at about a quarter of the length from the bleb base, and remained high at the neck where the bleb connects to the cells body (Fig. 2a–c). Quantification of the polarity index across the bleb confirmed this rear-to-front gradient, with the bleb base exhibiting around 2.5-fold enrichment of EGFR–GFP compared with the bleb tip (Fig. 2c). We found that EGFR activity itself was required for the rear-to-front EGFR gradient in leader blebs, as erlotinib treatment showed that inhibition of EGFR phosphorylation caused flattening of the EGFR–GFP rear-to-front gradient and significantly skewed the polarity index towards zero compared with DMSO control (Fig. 2d,e). We next tested whether the activation of EGFR differed from that of the total EGFR—that is, whether there was a local difference in sensitivity to EGFR activation within the bleb. We utilized a modular biosensor in which a FusionRed (FR)-labelled EGFR–CD3 chimera (EGFR–CD3–FR) recruits a clover-tagged tandem SH2 domain of ZAP70 (ZtSH2–CLV) to show where EGF signalling is active^45^. Live imaging showed that both constructs formed rear-to-front gradients, similar to the one seen for EGFR–GFP (Fig. 2f,g). Ratio imaging of ZtSH2–CLV to EGFR–CD3–FR showed that although there appeared to be a slight increase in the fraction of activated receptor towards the bleb tip, the polarity index of the ratio was close to zero, indicating that there was no spatial bias in sensitivity of EGFR (Fig. 2h,i). Together, these results demonstrate that EGFR localization and activity are highly polarized in a rear-to-front gradient in leader blebs during migration in low-adhesive confinement.

We next explored whether phospholipid and small GTPase signalling downstream of EGFR also exhibit a polarized distribution during leader-bleb-based migration. To visualize the subcellular localization of PI3K signalling, we expressed either a fusion of the PH domain of Akt with GFP (Akt-PH–GFP) as a biosensor for PtdIns(3,4,5)P_3_ localization^46^ or a fusion of the PH domain of PLCδ with GFP (PLCδ-PH–GFP) to report on PtdIns(4,5)P_2_^47^ (Fig. 2j). Quantification of images showed that the polarity index of Akt-PH–GFP (PtdIns(3,4,5)P_3_) within leader blebs was similar to that of EGFR, with a high intensity at the base of the bleb and low levels at the tip (Fig. 2j,k). By contrast, PLCδ-PH–GFP (PtdIns(4,5)P_2_) revealed a front-to-rear gradient that was inverted relative to that of PtdIns(3,4,5)P_3_, with highest concentration at the tip of the leader bleb (Fig. 2j,k). We next expressed the GTP-binding domains (GBD) of either Pak fused to YFP (Pak-GBD–YFP) or Rhotekin (Rtkn) fused to GFP (Rtkn-RBD–GFP), which act as GTP-dependent binding sensors for Rac1 or RhoA, respectively. This showed that Pak-GBD–YFP, and thus Rac1 activity, concentrated at the base of blebs, whereas Rtkn-RBD–GFP, and thus RhoA activity, localized toward the bleb tip (Fig. 2l,m). Thus, cells in low-adhesive confinement spontaneously polarize EGFR in the leader-bleb plasma membrane to localize PI3K, promoting PtdIns(3,4,5)P_3_ accumulation and Rac1 activity in rear-to-front gradients, and localize their antagonists, PtdIns(4,5)P_2_ and RhoA activity, to the bleb tip. Furthermore, this rear-to-front polarization of signalling in leader-bleb-based migration is the inverse of the canonical front-to-rear polarization of the same signalling factors in mesenchymal migration.

### Rear-to-front gradients of EGFR and RAC1 activities are required for leader-bleb stabilization

**Fig. 3:**
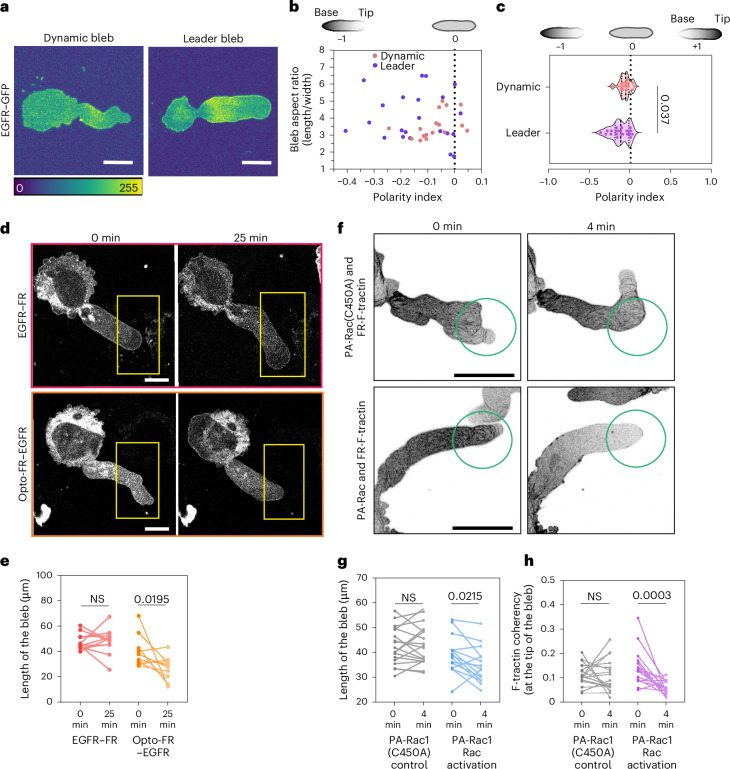
EGFR and Rac1 activity at the rear of the bleb is required for bleb stability. **a**, Intensity-pseudocoloured confocal image of EGFR–GFP in A375M2 melanoma cells under 3-μm confinement exhibiting dynamic elongated bleb (left) or leader-bleb (right) morphology. Scale bars, 20 μm. **b**, Scatter plot of bleb aspect ratio of dynamic elongate blebs (rose, *n* = 17 blebs) and leader blebs (purple, *n* = 22 blebs) versus polarity index of EGFR–GFP in these blebs. **c**, Violin plot of polarity indices of EGFR–GFP in morphologies shown in **a**. *P* = 0.037, two-tailed unpaired *t*-test with Welch’s correction. **d**, EGFR–FR and Opto-EGFR. Bleb morphology before (left) and after continuous activation illumination for 25 min (right) in yellow boxed region at the tip of the bleb. Scale bars, 20 μm. **e**, Quantification and pairwise comparison of the length of the bleb before (dark points) and after (light points) activation illumination in cells expressing EGFR–FR (*n* = 11 blebs) or Opto-EGFR (*n* = 10 blebs, *P* = 0.0195. **f**, Inverted contrast confocal images of FR–F-tractin in cells co-expressing either PA-Rac1(C450A) (top) or PA-Rac1 (bottom) before (left) and after (right) continuous activation illumination for 4 min in the region of the green circle. Scale bars, 20 μm. **g**, Quantification and pairwise comparison of the length of the bleb before (dark points) and after (light points) activation illumination in cells expressing PA-Rac1(C450A) (*n* = 18 blebs) or PA-Rac1 (*n* = 15 blebs, *P* = 0.0215). **h**, Quantification and pairwise comparison of FR–F-tractin coherency before (dark points) and after (light points) photoactivation for the same conditions and cells as in **g**. *P* = 0.0003. **e**,**g**,**h**, *N* = 3 independent experiments, two-tailed Wilcoxon matched-pairs rank test.

We next examined the requirements for the rear-to-front gradients of EGF activity and its downstream signalling in maintaining leader-bleb stability during leader-bleb-based migration. To approach this, we first investigated whether the degree of EGFR polarization correlated with bleb morphology or stability. We analysed aspect ratio as a function of EGFR–GFP polarity index for either dynamic elongated blebs or stabilized leader blebs (Fig. 3a–c). This showed that bleb aspect ratio did not directly correlate with EGFR polarity for either dynamic or stable elongated blebs. However, we found that even if dynamic blebs had a high aspect ratio, they did not have a high polarity index (Fig. 3b). Direct comparison of EGFR polarity index for dynamic elongated blebs versus stabilized leader blebs confirmed this, with leader blebs showing significantly higher EGFR polarity than transient elongated blebs (Fig. 3c). To determine the requirement for the rear-to-front EGFR and Rac1 activity gradients in stabilization of leader blebs, we utilized an optogenetic approach to spatially mislocalize these signalling activities to the bleb tip. To mislocalize EGFR or Rac1 activity, cells were transfected with either Opto-EGFR^48,49^ (or EGFR–FR as a control) or photoactivatable Rac1 (PA-Rac1) (or non-photoactivatable control (PA-Rac1(C450A)) as a control). A region of the leader-bleb tip was then exposed to repeated pulses of 405 nm light to locally induce clustering and activation of Opto-EGFR (Fig. 3d) or relieve steric hindrance from PA-Rac1 to allow Rac1 activation^50–52^. Time-lapse imaging of cells expressing Opto-EGFR showed that activation in the bleb tip caused a significant decrease in leader-bleb length after 25 min compared with similar light exposure on the bleb tip of cells expressing EGFR-FR (Fig. 3e). In cells co-expressing PA-Rac1 and FR–F-tractin, activation in the bleb tip was sufficient to decrease bleb length (Fig. 3f,g), cause local disassembly of the actin bundles to reduce actin filament coherence at the bleb tip, drive assembly of tiny lamellipodial actin protrusions, and eventually retract the bleb (Fig. 3f,h and Supplementary Video 5). This was in contrast to the lack of actin remodelling or retraction in response to similar light exposure at bleb tips in cells expressing PA-Rac1(C450A) (Fig. 3f–h). Together, these results demonstrate that the rear-to-front gradients of EGFR and Rac1 activities are required for bleb stability and maintenance to drive persistent migration in low-adhesive confinement.

### Rear-to-front EGFR gradient is maintained by local restriction of EGFR mobility near the bleb base

**Extended Data Fig. 2:**
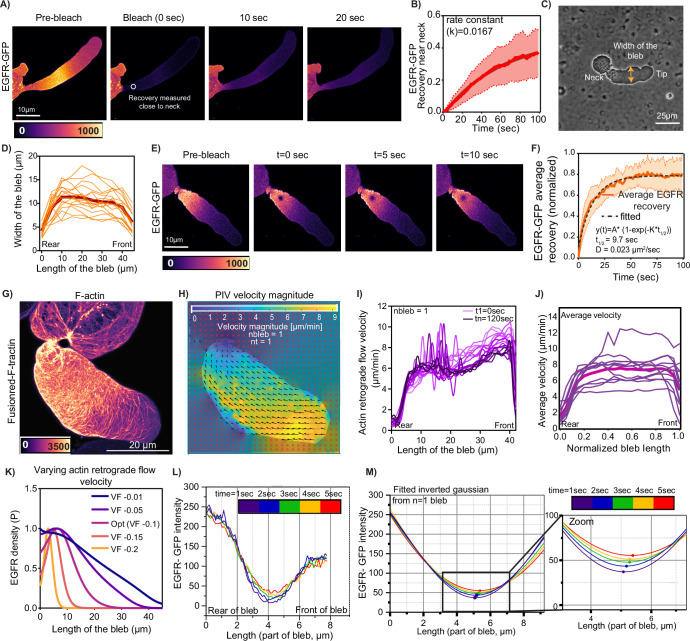
Characterization of EGFR and actin dynamics in leader blebs. (**A**, **E**, **G**) Confocal and (**C**) phase-contrast images of A375M2 melanoma cells under 3μm confinement. **A**) Pseudocolored intensity image of cells expressing EGFR-GFP that were subjected to photo-bleaching of the leader bleb (left-center panel) and time-lapse imaging during fluorescence recovery (right 2 panels). (**B**). Average normalized fluorescence intensity over time (solid lines) +/- standard deviation (dotted lines and shaded area), single exponential least squares fit (gray line) from small areas near the bleb neck (example region shown in second panel) in cells subjected to photobleaching like that shown in (**A**), Rate of recovery k = 0.0167, n = 18 cells. (**C**) Phase contrast image of cell undergoing leader bleb-based migration under high confinement, illustrating parameters plotted in (**D**) Bleb width as a function of position along the bleb, light orange= individual cells, dark orange= average, (n = 14 cells). (**E**) Pseudocolored intensity image of cells expressing EGFR-GFP that were subjected to photo-bleaching of small area near the base of the bleb (t = 0 sec) and time-lapse imaging during fluorescence recovery. (**F**) Average normalized fluorescence intensity over time (solid line, shaded area +/- SD) from photobleached regions like that shown in (**E**), time zero is the first image after photobleaching. Single exponential least squares fits (dotted lines), where n = 9 cells. (**G**) Pseudocolored intensity super-resolution image of a leader bleb in a cell expressing FusionRed-F-tractin to label actin filaments. (**H**) PIV analysis from two consecutive frames of a time-lapse movie of FusionRed-F-tractin of the bleb in (**G**) showing the direction (arrows) and magnitude (color encoded) of actin retrograde flow. (**I**) Actin retrograde flow velocities as a function of position along the bleb from a single bleb over time (light purple, t1 = 0 sec to dark purple, tn=120 sec) (**J**) Actin retrograde flow velocities as a function of normalized position along the leader bleb. Purple lines= individual cells, magenta line= average of n = 14 cells. (**K**) Outcome of the mathematical model. EGFR density along the length of the leader bleb with varying actin retrograde flow velocities, with optimum being the measured actin retrograde flow velocity. EGFR density (**P**) with changes in actin retrograde flow where measured ${V}_{F}\left(x\right)=-0.10+0.08{e}^{-0.2x}$ (μm/s) was taken optimum. Following retrograde flow velocities were considered: 0.01, 0.05, 0.1 (optimum), 0.15, 0.2 (μm/s). ${\varepsilon }_{1}(x)$ which reflects the ‘strength’ of membrane protein mobility restriction was set to 0.1 (weak) while ${\varepsilon }_{2}(x)$ which reflects the interaction with flowing F-actin was set constant to 0.5 (strong) (**L**) Intensity line-scans of EGFR-GFP over time from the highlighted position across the photobleached mark in Fig. (4, upper panels), taken at one-second intervals (time color encoded). (**M**) Left and Right: Linescan traces from the graph in (**L**) were fitted with an inverse gaussian and the minima of the fit is marked with a point, time color encoded. Right: Zoom of the minima region of the graph.

We next approached the question of the physical mechanism by which the rear-to-front gradient of EGFR is maintained within leader blebs in cells migrating in low-adhesive confinement. We began by ruling out the possibility that the EGFR gradient was maintained by spatial restriction of endocytosis or exocytosis. We photobleached EGFR–GFP throughout the entire bleb and examined the recovery pattern by time-lapse imaging. This showed that recovery was very slow (Extended Data Fig. 2a,b and Supplementary Video 6), with no localized bursts of EGFR–GFP anywhere along the length of the bleb that would be indicative of exocytosis of EGFR-containing vesicles^53,54^. Furthermore, previous studies show that leader-bleb cells under confinement have their secretory and endocytic apparatus localized almost exclusively in the cell body and that they do not extend into the bleb^55,56^.

**Fig. 4:**
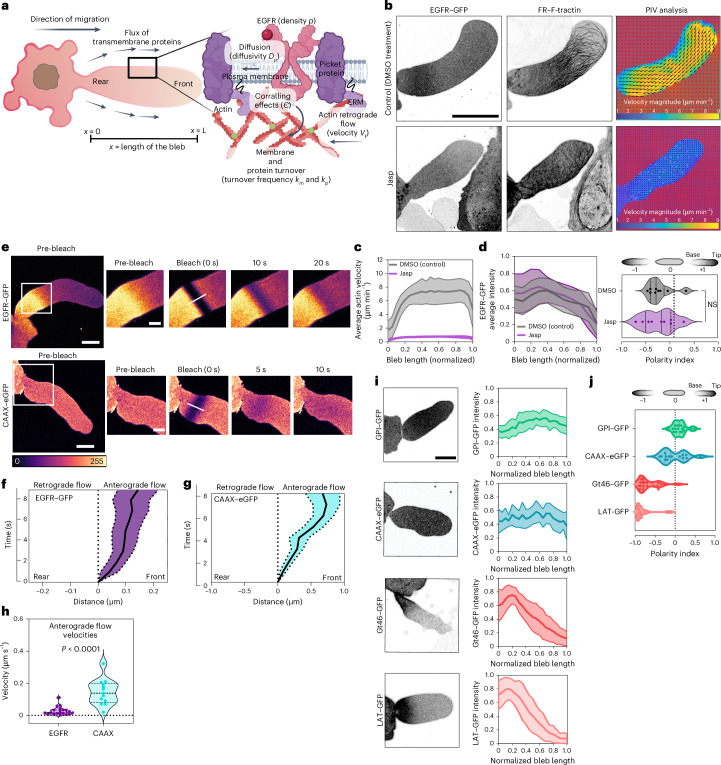
Experiment and modelling indicate that neither cortical nor membrane flow mediate EGFR gradient formation in leader blebs. **a**, Schematic of a confined cell undergoing leader-bleb-based migration (left) and plasma membrane components (right). The direction of migration (large arrow, top) and transmembrane protein flux (small arrows around the bleb) are indicated. EGFR (pink) with its ligand EGF (red), picket proteins (purple), ERM linker proteins (light purple), actin cytoskeleton (red) and plasma membrane (grey) are shown. Canonical mechanisms regulating receptor segregation, diffusion (*D*_p_), advection by actin retrograde flow (*V*_f_), corralling (*ε*) and membrane and protein turnover (*k*_m_ and *k*_p_, respectively) are illustrated in semi-transparent overlays. **b**,**e**, Representative confocal images of blebs in A375M2 melanoma cells under 3-µm confinement. **b**, Inverted contrast images of blebs in cells co-expressing EGFR–GFP (left) and FR–F-tractin (middle), treated with vehicle (DMSO, top) or jasplakinolide (Jasp; 100 nM, bottom). Right, PIV analysis of FR–F-tractin time-lapse videos showing direction (arrows) and magnitude (colour-coded) of actin retrograde flow between consecutive frames. Scale bar, 20 μm. **c**, Average actin retrograde flow velocity (dark line shows mean and shaded area represents s.d.) in blebs of DMSO-treated (*n* = 14 cells) or jasplakinolide-treated (*n* = 18 cells) cells as a function of normalized bleb length (*N* = 3 experiments). **d**, Left, average normalized EGFR–GFP intensity (dark line shows mean and shaded area represents s.d.) along the bleb length in DMSO-treated (*n* = 10) or jasplakinolide-treated (*n* = 11) cells. Right, violin plot of EGFR polarity indices; two-tailed Welch’s *t*-test. **e**, Pseudocoloured time-lapse images of blebs expressing EGFR–GFP (top) or CAAX–eGFP (bottom) following photobleaching of a rectangular region (*t* = 0 s). Right: fluorescence recovery; white lines indicate line-scan positions. Scale bars, 10 μm (larger views), 5 μm (insets). **f**,**g**, Displacement of the minima of inverted Gaussian fits to line scans relative to the bleached position over time for EGFR–GFP (**f**; *n* = 21 blebs) and CAAX–eGFP (g; *n* = 14 blebs). Dark line shows mean and shaded area represents s.d. **h**, Violin plot of anterograde flow velocities measured from photobleaching experiments; each dot represents one bleb. Two-tailed unpaired *t*-test with Welch’s correction. **i**, Inverted contrast images of cells expressing GPI–GFP, CAAX–GFP, Gt46–GFP or LAT–GFP (left), with corresponding average normalized intensity profiles (dark line shows mean and shaded area represents s.d.) along the bleb length (right). Scale bar, 10 μm. **j**, Violin plots of polarity indices for GPI–GFP (*n* = 16), CAAX–GFP (*n* = 14), Gt46–GFP (*n* = 24) and LAT–GFP (*n* = 20). Panel **a** created in BioRender; Jha, A. https://biorender.com/w9sb9y6 (2026).

To differentiate between the hypotheses of advection and local restriction of free mobility as possible mechanisms of maintaining a rear-to-front gradient of EGFR in leader blebs, we used a combination of mathematical modelling and experiments. The model was based on the assumptions that EGFR and membrane synthesis occur in the cell body, the bleb has a geometry based on our average measurements (Extended Data Fig. 2c,d), EGFR has an apparent diffusivity based on fluorescence recovery after photobleaching (FRAP) measurements (lateral diffusion coefficient (*D*_p_); Extended Data Fig. 2e,f), a forward advection velocity from bleb base to tip (*V*_p_), a negative retrograde cytoskeletal flow velocity (*V*_f_; Extended Data Fig. 2g–j) and a turnover (degradation or endocytic removal) frequency (membrane *k*_m_ and protein *k*_p_), each of which can potentially vary with position along the bleb length (*L*) (Fig. 4a). An important insight gained from initial consideration that EGFR and membrane synthesis occur in the cell body was that both bulk membrane and EGFR must both undergo a net anterograde movement from the bleb base towards the tip (Supplementary Note). This was unexpected, and in opposition to the notion that advective movement via retrograde cytoskeletal flow would mediate the formation of the rear-to-front EGFR gradient.

We thus explored the possible contribution of advection by actin retrograde flow in forming the EGFR gradient^30^. We first characterized actin dynamics in leader blebs to obtain parameters for our model. Imaging and particle image velocimetry (PIV) analysis of F-tractin–FR showed, similar to previous reports^20,28^, that actin retrograde flow was slow at the bleb tip, quickly becoming fast in the middle, and slower again at the bleb base (Extended Data Fig. 2g–j and Supplementary Video 7). Modelling the effect of this actin retrograde flow profile on EGFR distribution produced a rear-to-front gradient of EGFR, similar to the measured distribution. Increasing or decreasing the actin retrograde flow speed changed the slope of the gradient (Extended Data Fig. 2k), suggesting that an EGFR gradient could be modulated by cytoskeletal advection, as has been suggested before^30^. To test this experimentally, we perfused leader-bleb cells expressing F-tractin–FR and EGFR–GFP with jasplakinolide to inhibit the actin treadmilling that drives retrograde flow^20^ (Fig. 4b and Supplementary Video 8). PIV analysis confirmed that jasplakinolide strongly hindered actin retrograde flow compared with DMSO control (Fig. 4b,c), but unexpectedly, had no significant effect on the polarity index of EGFR distribution along the bleb (Fig. 4d and Supplementary Video 8). These results show that although advection by cytoskeletal flow could in theory mediate formation of a rear-to-front EGFR gradient in leader blebs, experimental evidence surprisingly shows that this gradient is not established by actin retrograde flow.

We then sought to assess the model prediction that EGFR moved from the cell body towards the bleb tip. We expressed either EGFR–GFP or a bulk membrane marker consisting of GFP fused to the CAAX prenylation signal sequence (CAAX–GFP) (Fig. 4e), photobleached them in a stripe near the base of the bleb, and analysed the directionality of the intensity recovery by determining the position of the minima of an inverted Gaussian fitted to the bleach stripe over time (Extended Data Fig. 2l,m). This showed that photobleached stripes in either EGFR–GFP or CAAX–GFP exhibited anterograde movement from the bleb base towards the tip, but with different apparent velocities, with CAAX–GFP moving significantly faster than EGFR–GFP (Fig. 4f–h and Supplementary Video 9). Together, these results support the model prediction that EGFR enters the bleb plasma membrane by a net anterograde movement from the cell body towards the bleb tip, and that EGFR movement is somehow slowed relative to that of the bulk membrane.

We then explored what type of membrane association could possibly cause a protein to resist anterograde membrane flow and accumulate at the bleb rear to form a rear-to-front gradient. We analysed the distribution of different types of membrane-associated markers within the leader bleb, including other transmembrane proteins besides EGFR: Gt46–GFP (an artificial secretory protein containing a signal sequence, GFP, a consensus N-glycosylation site, the transmembrane domain of the LDL receptor, and the cytoplasmic tail of CD46^57^), LAT–GFP, an inner leaflet-associated marker (CAAX–GFP), or an outer leaflet-associated marker (GFP fused to glycosylphosphatidylinositol (GPI–GFP))^57^. Imaging and analysis of polarity index of these markers in leader blebs showed that, similar to EGFR–GFP, the transmembrane proteins Gt46–GFP and LAT–GFP formed rear-to-front gradients, accumulating towards the base of the bleb. By contrast, the inner and outer leaflet membrane markers CAAX–GFP and GPI–GFP were evenly distributed along the bleb (Fig. 4i,j). These results suggest that transmembrane proteins in general are capable of resisting anterograde membrane flow to mediate their accumulation in rear-to-front gradients along leader blebs.

**Fig. 5:**
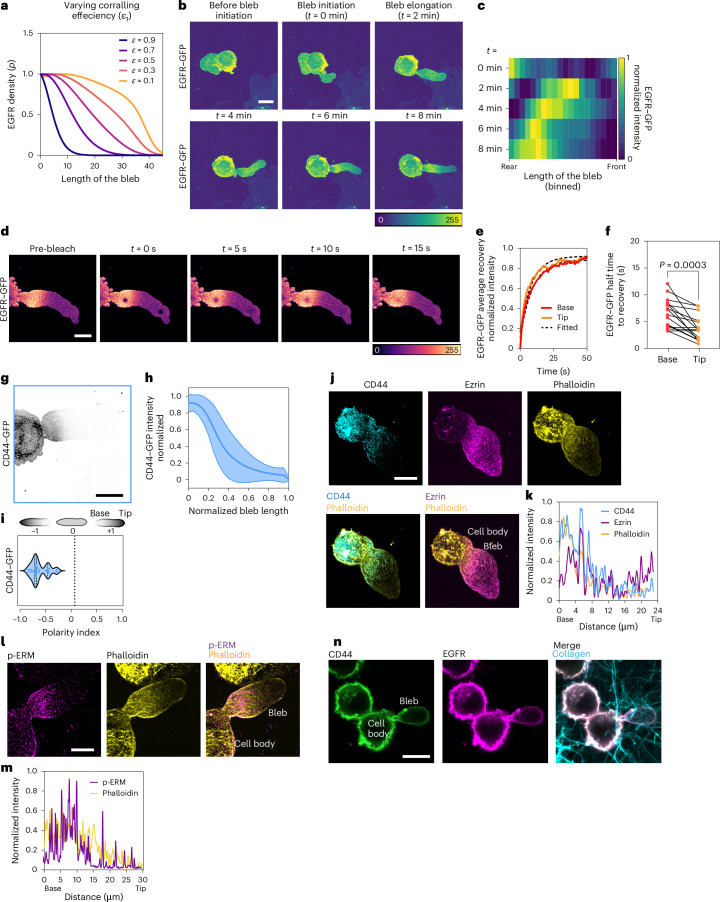
EGFR has restricted diffusion at the rear of the leader bleb. **a**, Outcomes of the transmembrane protein gradient mathematical model. EGFR receptor density as a function of length along the bleb under different corralling efficiencies *ε*, with actin retrograde flow velocity *V*_f_(*x*) = −0.10 + 0.80e^−0.2*x*^ (μm s^−1^) and the interaction with flowing F-actin *ε*_2_(*x*) set to 0.1 (weak interaction). **b**,**d**,**g**,**n**, Representative confocal images of blebs in live A375M2 melanoma cells under 3-µm confinement (**b**,**d**,**g**) or migrating within a 3D fibrillar collagen ECM (**n**). **b**, Left: intensity-pseudocoloured time-lapse images showing initiation and elongation of a leader bleb in a cell expressing EGFR–GFP. Scale bar, 20 μm. **c**, Heat map of normalized EGFR–GFP intensity as a function of normalized bleb length over time (binned). **d**, Intensity-pseudocoloured time-lapse images of a leader bleb expressing EGFR–GFP following photobleaching of two small regions near the bleb base and tip. Scale bar, 10 μm. **e**, Average normalized fluorescence intensity over time from the two photobleached regions shown in **d**. Time zero corresponds to the first frame after bleaching. Solid lines indicate measured intensity and dotted lines show single-exponential least-square fits. **f**, Half-times of FRAP derived from fits as in **e**. Paired data represent bleb base and tip within the same cell (*n* = 16 blebs; two-tailed paired *t*-test). **g**, Inverted contrast image of a cell expressing CD44–GFP. Scale bar, 10 μm. **h**,**i**, Mean normalized CD44–GFP intensity along the leader bleb (**h**; dark line shows mean and shaded area represents s.d.) and violin plot of CD44–GFP polarity indices (**i**). *n* = 22 cells. **j**, Immunofluorescence staining of CD44, ezrin and F-actin (phalloidin) (top), with merged images of CD44 and F-actin (bottom left) and ezrin and F-actin (bottom right). Scale bar, 10 μm. **k**, Normalized intensity line-scan analysis across the leader bleb shown in **j**, from bleb base to tip. **l**,**m**, Immunostaining of phospho-ERM (p-ERM) and F-actin (**l**, left), merged images (**l**, right) and corresponding normalized intensity line-scan analysis from bleb base to tip (**m**). Scale bar, 10 μm. **n**, Immunostaining of CD44 and EGFR in an A375M2 cell migrating in 3D collagen (left), with collagen fibres shown in cyan in the merged image (right). Scale bar, 10 μm.

We next pursued the question of how transmembrane proteins resist anterograde membrane flow. We modelled the effect of restriction of EGFR mobility relative to that of the bulk membrane by ‘corralling’ (*ε*_1_(*x*)), but while considering minimal advection by retrograde actin flow (thus *ε*_2_(*x*) = 0.1), in line with our experimental observations. Our model showed that corralling EGFR in the leader-bleb rear resulted in a rear-to-front EGFR gradient that decreased steepness with decreasing corralling (Fig. 5a). To determine whether restriction of EGFR mobility could cause its rear-to-front gradient in cells, we first analysed the evolution of EGFR–GFP distribution during initial formation of an elongated bleb. We found that as the bleb elongated, EGFR–GFP accumulated at the base of the bleb and never advanced towards the bleb tip, suggesting that EGFR–GFP was corralled at the rear (Fig. 5b,c and Supplementary Video 10). We then performed FRAP to simultaneously bleach small regions near both the bleb base and tip, and analysed their intensity recoveries over time (Fig. 5d and Supplementary Video 11). This showed that EGFR–GFP intensity recovery at the bleb base was significantly slower than at the bleb tip, indicating a locally reduced mobility of EGFR (Fig. 5e,f). Together, these results suggest that a rear-to-front gradient of EGFR along leader blebs is generated and maintained by restriction of lateral mobility of transmembrane proteins in the bleb rear, which mediate bleb stabilization and drive persistent migration in low-adhesive confinement.

### CD44, Ezrin and a membrane-apposed actin meshwork restrict EGFR mobility at the bleb base to establish the rear-to-front EGFR gradient in leader blebs

**Extended Data Fig. 3:**
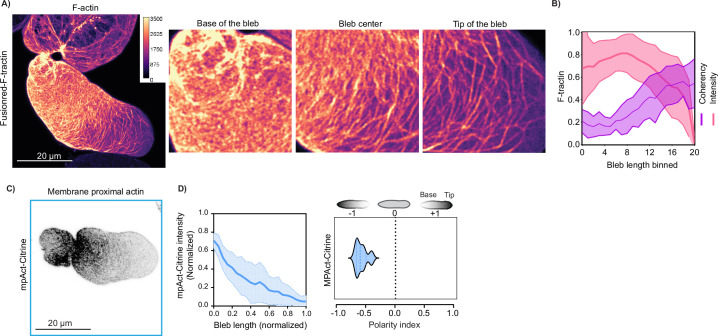
A dense actin meshwork forms close to the plasma membrane at the rear of leader blebs. (**A**, **C**) Confocal images of A375M2 melanoma cells under 3μm confinement. (**A**) Left: Pseudocolored intensity super-resolution image of cell expressing Fusionred-F-tractin. Right panels: Zooms of the noted regions of bleb in the cell at (right), showing the actin architecture at the base (left center), center (right center) and tip (right) of the bleb. (**B**) Average normalized intensity (red) and actin filament coherency (purple) (thick lines) +/- standard deviation (thin lines and shaded areas) of FusionRed-F-tractin as a function of binned position along the bleb length, n = 22 cells. (**C**) Inverted grayscale image of mpAct-Citrine. (**D**) Left panel: Average normalized intensity (thick line) +/- standard deviation (dotted lines and shaded areas) of cells expressing mpAct-citrine like in (**C**) as a function of position along the normalized bleb length, right panel: Violin plot of polarity index of mpAct-citrine in blebs. n = 7 cells from 3 experiments.

We next sought to determine the molecular basis of EGFR mobility restriction to maintain its gradient along leader blebs. As CD44 and ERM (ezrin–radixin–moesin) proteins are known to link to the sub-membrane cortical actin meshwork to create membrane corrals that restrict free diffusion of transmembrane proteins in other cell types^36^ and ezrin is crucial for bleb stability^19,28^, we examined their distributions in leader blebs. We found that leader blebs in cells expressing CD44–GFP in low-adhesive confinement showed an asymmetric localization, with a high concentration at the rear of the bleb that decreased towards the tip (Fig. 5g–i). Immunostaining for CD44, ezrin or active, phosphorylated ERM proteins (phospho-ERM) in confined cells revealed a concentration of CD44 in the cell body and both ezrin and phospho-ERM concentrated in the leader bleb. Line-scan analysis of leader blebs showed that whereas ezrin was localized throughout the bleb, CD44 and phospho-ERM formed rear-to-front gradients (Fig. 5j–m). Notably, immunostaining CD44 and EGFR in melanoma cells migrating in a dense 3D collagen matrix showed that, like cells in PDMS confinement, cells formed leader blebs that displayed CD44 and EGFR rear-to-front gradients in this more physiologically relevant environment (Fig. 5n). We next examined whether actin organization was conducive to corral formation in leader blebs. Analysis of FR–F-tractin organization showed that actin filament coherence was low towards the bleb base, indicating a dense actin meshwork, and increased towards the bleb tip where sparser bundles formed (Extended Data Fig. 3a,b). To determine whether the actin meshwork was in close proximity to the plasma membrane, we utilized the MPAct–citrine biosensor that diffuses freely in the membrane except in regions where cortical actin is within about 10 nm from the plasma membrane, where it becomes immobilized and enriched by binding actin^58^. This analysis revealed that membrane-proximal actin was predominantly located at the rear of the bleb (Extended Data Fig. 3c,d). Together, these results show that CD44 and activated ERM proteins are concentrated at the rear of leader blebs where the actin cortex forms a dense meshwork in close proximity to the membrane, suggesting these components could form membrane corrals that locally restrict the mobility of EGFR in cells migrating in confined microenvironments.

**Fig. 6:**
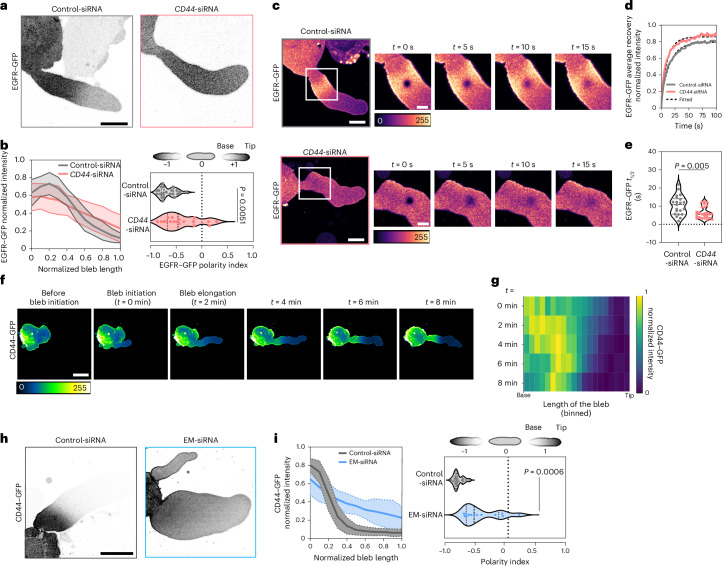
CD44 restricts the diffusion of EGFR at the rear of the leader bleb. **a**,**c**,**f**,**h**, Confocal images of blebs in A375M2 melanoma cells under 3-μm confinement. **a**, Inverted contrast image of blebs in cells expressing EGFR–GFP and either scramble control siRNA (left) or siRNA targeting *CD44* (right). Scale bar, 10 μm. **b**, Left: mean normalized intensity profile of EGFR–GFP across the normalized length of the leader bleb (dark line shows mean and shaded area represents s.d.) from line-scan analysis of cells under treatments shown in **a**, and violin plot of EGFR–GFP polarity index (Control-siRNA, *n* = 21 cells; *CD44*-siRNA, *n* = 20 cells). *P* = 0.0051, two-tailed unpaired *t*-test with Welch’s correction. Scale bars, 10 μm (larger views), 5 μm (insets). **c**, Intensity-pseudocoloured time-lapse images of a leader bleb in a cell expressing EGFR–GFP together with either scrambled (control) siRNA (top row) or siRNAs targeting *CD44* (bottom row), with photobleaching in a small region near the rear of the bleb. **d**, Average normalized fluorescence intensity over time (solid lines) from the photobleached regions in the cells in **c**; time zero is the first image after photobleaching. Dotted lines show single-exponential least squares fits. **e**, Violin plot of the half-times of FRAP determined from single-exponential fits of data such as those represented in **d**; (Control-siRNA, *n* = 21 cells; *CD44*-siRNA, *n* = 20 cells). *P* = 0.005, two-tailed unpaired *t*-test with Welch’s correction. **f**, Intensity-pseudocoloured time-lapse images of leader-bleb formation in a cell expressing CD44–GFP as the bleb initiates and elongates over time. Scale bar, 20 μm. **g**, Heat map of the normalized CD44–GFP intensity over time as a function of normalized length of bleb (binned). **h**, Inverted contrast image of blebs in cells expressing CD44–GFP and either scramble control siRNA (left) or siRNAs targeting ezrin and moesin (EM-siRNA). Scale bar, 10 μm. **i**, Left: mean normalized intensity profile of EGFR–GFP across the normalized length of the leader bleb (dark line shows mean and shaded area represents s.d.), Control-siRNA (*n* = 16 cells) or EM-siRNA (*n* = 13 cells). *N* = 3 independent experiments. Right: violin plot of CD44–GFP polarity indices for the conditions in **h**,**i**. *P* = 0.0006, two-tailed unpaired *t*-test with Welch’s correction.

**Extended Data Fig. 4:**
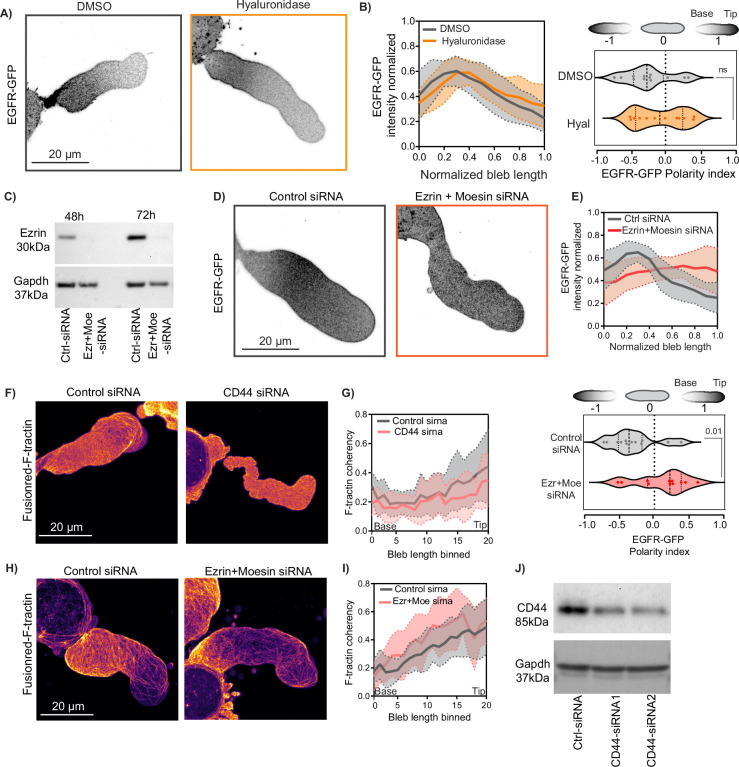
Role of CD44 and ERM proteins in EGFR and actin organization in leader blebs. (**A**, **D**, **F**, **H**) Confocal images of A375M2 melanoma cells under 3μm confinement. (**A**) Inverted contrast image of cells expressing EGFR-GFP treated with either DMSO or Hyaluronidase. (**B**) Left panel: Average normalized intensity (dark lines) +/- standard deviation (light lines and shaded areas) of EGFR-GFP as a function of normalized position along the normalized bleb length in cells treated with DMSO (gray, n = 12 cells) or Hyaluronidase (20 units/ml, orange, n = 12 cells). Right panel: Violin plot of polarity indices of EGFR-GFP in blebs of the cells analyzed at left, Two-tailed unpaired t-test with Welch’s correction. (**C**) Western blots of lysates of cells transfected with either non-targeting control siRNAs (ctrl-siRNA) or siRNAs targeting ezrin and moesin (ezr+moe siRNA) for either 48 and 72 hours. Blots probed for Ezrin and GAPDH as a loading control. (**D**) Inverted contrast image of cells expressing EGFR-GFP co-transfected with control-siRNA or siRNAs targeting ezrin and moesin. (**E**) Average normalized intensity (dark lines) +/- standard deviation (dotted lines and shaded areas) of EGFR-GFP as a function of normalized position along the bleb length in cells transfected with control-siRNA (gray, n = 12 cells) or siRNAs targeting ezrin and moesin (red, n = 11 cells). Right panel: Violin plot of polarity indices of EGFR-GFP in blebs of the cells analyzed at left, p = 0.01; Two-tailed unpaired t-test with Welch’s correction (**F**) Pseudo-colored intensity image of cells expressing FusionRed-F-tractin and co-transfected with control-siRNA (left) or CD44-siRNA (center). (**G**) Average normalized F-actin coherency (wide lines) +/- standard deviation (dotted lines and shaded areas) as a function of normalized position along the bleb length in cells co-transfected with control-siRNA (gray, n = 8 cells) or CD44-siRNA (pink, n = 9 cells). (**H**) Pseudo-colored intensity image of cells expressing FusionRed-F-tractin and co-transfected with control-siRNA (left) or ezrin and moesin-siRNA (center). (**I**) Average normalized F-actin coherency (thick lines) +/- standard deviation (dotted lines and shaded areas) of F-tractin staining as a function of normalized position along the bleb length in cells co-transfected with control-siRNA (gray, n = 10 cells) or ezrin and moesin-siRNA (pink, n = 10 cells). (**J**) Western blots of lysates of cells transfected with either non-targeting control siRNAs (ctrl-siRNA) or siRNAs targeting cd44 siRNA with two different sequences 1 and 2. Blots probed for CD44 and GAPDH as a loading control.

We next tested the requirement for CD44 and ERM proteins in restricting EGFR mobility in the plasma membrane to maintain the rear-to-front EGFR gradient in leader blebs. Cells were co-transfected with EGFR–GFP and pooled siRNAs targeting either *CD44*, ezrin (*EZR*) and moesin (*MSN*) (EM; the predominant ERMs expressed in A375 cells^14,59^) or non-targeting controls, and subjected to low-adhesive confinement (Fig. 6a). Analysis showed that either CD44 knockdown or EM knockdown (Extended Data Fig. 4c) caused flattening of the EGFR–GFP rear-to-front gradients and significantly skewed the polarity indices towards zero compared with non-targeting controls (Fig. 6b and Extended Data Fig. 4d,e). FRAP analysis for spots bleached near the bleb base showed that reducing CD44 levels caused significantly faster recovery compared with non-targeting control, confirming that CD44 reduces the mobility of EGFR–GFP (Fig. 6c–e and Supplementary Video 12). Treatment of cells expressing EGFR–GFP with hyaluronidase to degrade polyhyaluronic acid had no significant effect on the EGFR–GFP rear-to-front gradient or polarity index in leader blebs, indicating that the link between CD44 and the extracellular glycocalyx was not critical to EGFR gradient formation (Extended Data Fig. 4a,b). Of note, imaging of FR–F-tractin showed that CD44 knockdown or EM knockdown caused disparate effects on actin organization, with CD44 knockdown slightly decreasing and EM knockdown increasing actin filament coherency (Extended Data Fig. 4f–i). Together, these results show that CD44 and ERM proteins are required for maintaining spatial organization of actin architecture in leader blebs to restrict the mobility of EGFR near the bleb base during leader-bleb-based migration.

We next investigated how CD44 is maintained in a polarized distribution to restrict EGFR mobility at the bleb base. Examining the evolution of CD44 localization during leader-bleb formation (Fig. 6f) showed that, like EGFR, CD44–GFP accumulated at the base of the bleb and never advanced towards the bleb tip as the bleb elongated, suggesting that it was trapped towards the bleb rear (Fig. 6g and Supplementary Video 13). Knockdown of ERM proteins showed that compared with non-targeting controls (Fig. 6h,i), EM knockdown caused flattening of the CD44–GFP intensity gradient and significantly skewed the CD44 polarity index towards zero (Fig. 6i). Taken together, our data demonstrates that ERM proteins promote the polarized distribution of CD44, and together with a membrane-proximal actin meshwork at the rear of the bleb, EGFR mobility is curtailed as the bleb elongates to establish a rear-to-front EGFR gradient in leader blebs.

### CD44 is required for polarization of signalling to promote leader-bleb stability and persistent migration in low-adhesive confinement

**Fig. 7:**
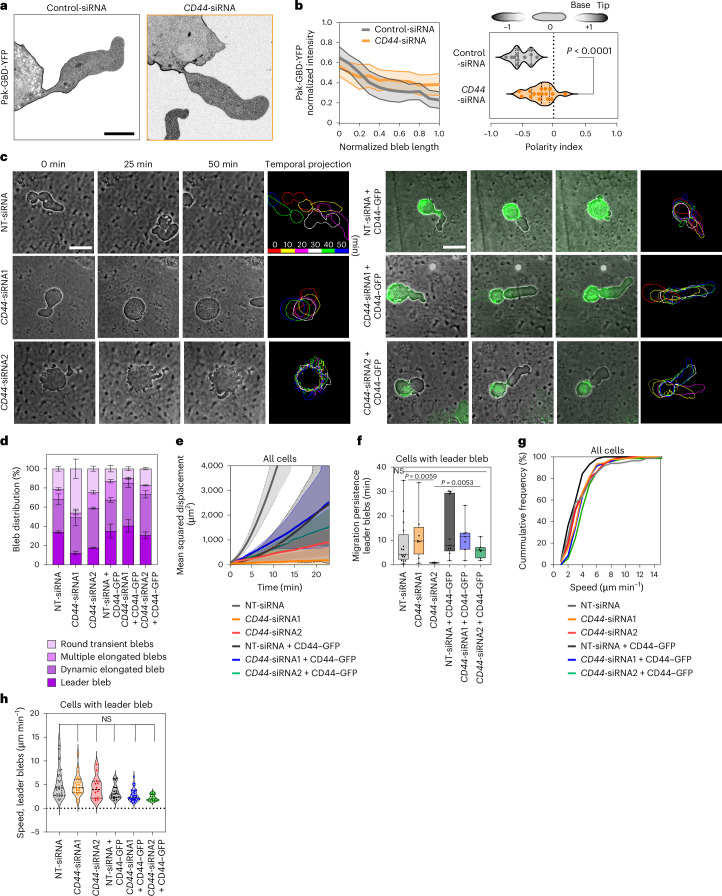
CD44 is required for rear-to-front Rac1 activity gradient and leader-bleb stability and persistent migration under confinement. **a**, Inverted contrast confocal images of cells co-expressing Pak-GBD–YFP, a reporter of active Rac1, following transfection with non-targeting control siRNA or *CD44*-siRNA. Scale bar, 10 μm. **b**, Left: mean normalized Pak-GBD–YFP intensity profiles along the normalized length of the leader bleb (dark line shows mean and shaded area represents s.d.) from line-scan analyses of cells shown in **a**. Right: violin plots of Pak-GBD–YFP polarity indices. Control siRNA (*n* = 13 cells) and *CD44*-siRNA (*n* = 16 cells); *P* = 0.0001, two-tailed unpaired *t*-test with Welch’s correction. **c**, Representative time-lapse phase-contrast images of A375M2 melanoma cells migrating under 3-µm confinement (left), with corresponding colour-encoded temporal projections of cell outlines at 10-min intervals (right). Cells were transfected with non-targeting control siRNA (NT-siRNA), *CD44*-siRNA targeting sequence 1 (*CD44*-siRNA1), *CD44*-siRNA targeting sequence 1 (*CD44*-siRNA1), or co-transfected with CD44–GFP (green) plus NT-siRNA, *CD44*-siRNA1 or *CD44*-siRNA2. Scale bars, 25 μm. **d**,**e**, Quantification of bleb phenotype distribution (**d**) and MSD over time (**e**; dark line shows mean and dotted line and shaded area represent s.d.) for conditions shown in **c**. NT-siRNA, *n* = 47; *CD44*-siRNA1, *n* = 122; *CD44*-siRNA2, *n* = 69; NT-siRNA + CD44–GFP, *n* = 77; *CD44*-siRNA1 + CD44–GFP, *n* = 121; *CD44*-siRNA2 + CD44–GFP, *n* = 75). **f**, Box plots of migration persistence for leader-bleb cells under the conditions in **c**. NT-siRNA, *n* = 16; *CD44*-siRNA1, *n* = 8; *CD44*-siRNA2, *n* = 2; NT-siRNA + CD44–GFP, *n* = 9; *CD44*-siRNA1 + CD44–GFP, *n* = 7; *CD44*-siRNA2 + CD44–GFP, *n* = 7). Statistical comparisons: NT-siRNA versus *CD44*-siRNA1, not significant; NT-siRNA versus *CD44*-siRNA2 (*P* = 0.0059); NT-siRNA versus NT-siRNA + CD44–GFP, not significant; NT-siRNA + CD44–GFP versus *CD44*-siRNA1 + CD44–GFP, not significant; NT-siRNA + CD44–GFP versus *CD44*-siRNA2 + CD44–GFP, *P* = 0.0053; two-tailed unpaired *t*-test with Welch’s correction. In box plots, the centre line is the median, box edges delineate interquartile range and whiskers extend to minimum and maximum values. **g**, Cumulative frequency distributions of migration speed for conditions in **c**. NT-siRNA, *n* = 4; *CD44*-siRNA1, *n* = 92; *CD44*-siRNA2, *n* = 77; NT-siRNA + CD44–GFP, *n* = 58; *CD44*-siRNA1 + CD44–GFP, *n* = 71; *CD44*-siRNA2 + CD44–GFP, *n* = 71. **h**, Violin plots of migration speed for leader-bleb cells under the same conditions (left to right: *n* = 30, 24, 12, 17, 30, 24); all comparisons not significant; two-tailed unpaired *t*-test with Welch’s correction on *N*. *n* represents number of cells, *N* = 3 independent experiments.

**Extended Data Fig. 5:**
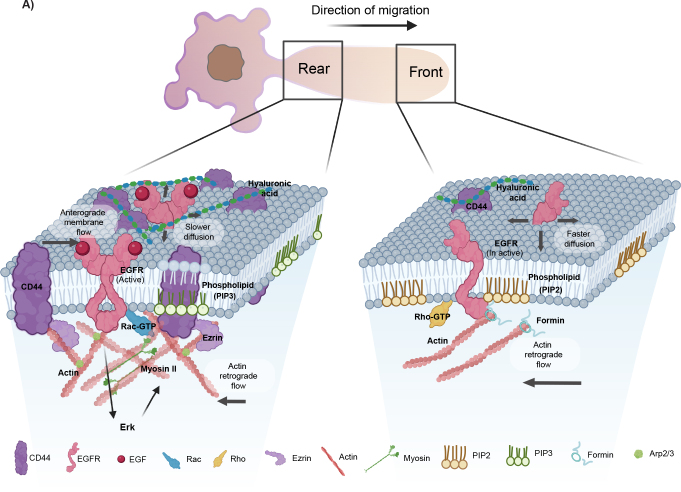
Model of molecular regulation of EGFR polarity during leader bleb-based migration under non-adhesive confinement. (**A**) Hypothetical model showing molecular mechanism of maintenance rear-tor front gradient of EGFR activity during leader bleb based migration. See discussion. Created in BioRender; Jha, **A**. https://BioRender.com/ktk0c4u (2026).

Next, we investigated the role of CD44-mediated corralling of EGFR mobility at the bleb base in promoting bleb polarity and persistent migration in low-adhesive confinement. We examined the effect of CD44 depletion on polarity signalling within leader blebs. Analysis of Pak-GBD–YFP in CD44-knockdown cells showed reduction in the rear-to-front polarization of Rac1 activity compared with non-targeting controls (Fig. 7a,b). Analysis of cell motility parameters showed that siRNAs targeting the 3′ untranslated region (UTR) of *CD44* (*CD44*-3′KD; Extended Data Fig. 4j) significantly inhibited leader-bleb formation (Fig. 7c,d and Supplementary Video 14) and markedly reduced the migratory range of cells in low-adhesive confinement (Fig. 7e). However, cells that managed to undergo leader-bleb-based migration exhibited persistent migration with similar velocity (Fig. 7f,h and Supplementary Video 14). Notably, reintroducing CD44–GFP into *CD44*-3′KD cells rescued the percentage of cells undergoing leader-bleb-based migration (Fig. 7d) and restored the migratory range to that of non-targeting controls (Fig. 7e), whereas overexpression of CD44 in non-targeting controls had no effect on leader-bleb formation. Together, our results show that during low-adhesive confinement, melanoma cells protrude elongated blebs that trap CD44 at their base owing to its interaction with the underlying actin cortex via ERM proteins, which in turn restricts EGFR mobility to mediate polarized signalling within the bleb to stabilize bleb morphology and promote persistent leader-bleb-based migration (Extended Data Fig. 5a).

## Discussion

Our study reveals a novel polarization of signalling driving bleb-based migration in metastatic melanoma cells under confinement that is the reverse of the classical model of cell polarity seen in integrin-dependent mesenchymal migration. Along with previous studies, our results show that confinement-induced intracellular pressure promotes a persistent, highly polarized, stable leader bleb containing an asymmetry in actin polymerization and contractility that drives actin retrograde flow, creating a feedback loop that sustains polarity in the absence of external chemical cues or integrin signalling^19–21,60^. We identify growth factor receptor and PI3K signalling as essential for stabilizing leader blebs. Using high-resolution microscopy and biosensors, we discovered a unique rear-to-front gradient of EGFR–PI3K activity, with PtdIns(3,4,5)P_3_ and Rac1 enriched at bleb rear, and their antagonists PtdIns(4,5)P_2_ and RhoA, concentrated at the bleb tip. Optogenetic mis-localization of EGFR or Rac1 activities caused bleb retraction, highlighting the requirement for this gradient in bleb stability. Our mathematical modelling predicted net anterograde EGFR movement from bleb base to tip, despite the presence of strong actin retrograde flow. Experiments revealed that during bleb initiation, CD44 and ERM proteins restrict EGFR lateral diffusion within a membrane-apposed cortical actin meshwork in the bleb rear, establishing a rear-to-front EGFR–PI3K–Rac gradient. Other RTKs such as HER2, FGFRs and MET that, like EGFR, also signal to PI3K–MAPK^61^ may similarly engage in this corralling-based mechanism to contribute to polarity during bleb-based migration. Thus, this study thus reveals the biophysical and molecular underpinnings of cell polarity in bleb-based migration in confined microenvironments (Extended Data Fig. 5a).

The same set of signalling modules arranged in opposite orientations appear to drive forward movement in both mesenchymal and leader-bleb-based migration. In mesenchymal migration, integrin binding to ECM at the leading edge activates PI3K–Rac1 signalling^62^, promoting Arp2/3-mediated, branched actin polymerization-driven protrusion of lamellipodia^63^, whereas Rho GTPase coordinates activation of myosin II contractility in the cell centre and rear to drive actin retrograde flow at focal adhesions^64,65^, acting as a regulatable molecular clutch to drive cells forward^23^. By contrast, in confinement, we found that EGFR–PI3K–Rac signalling localized at the bleb rear and activated RhoA concentrated at the bleb leading edge. Here, RhoA activity at the bleb tip is likely to mediate formin-dependent assembly of actin, which drives the retrograde flow of actin bundles that we observed^28^. The sparsity of these bundles at the bleb tip membrane enables pressure, as opposed to a growing actin meshwork^66^, to continue driving the leading edge forward. However, continuous actin assembly is still required to feed actin retrograde flow along the bleb length to sweep transmembrane, cytoskeleton-associated proteins rearward to mediate their non-specific friction with the substrate that drives the cell forward^20^. The transition from actin bundles at the bleb tip to meshwork at the bleb rear is probably mediated by the spatial switch between leading edge Rho-driven formin activity to Rac1-driven Arp2/3-mediated actin meshwork assembly. Why leading edge Rho activity does not promote contractility at the bleb tip is not clear, however both Rac1 and CD44 have been shown to promote myosin contractility in migrating cells^67,68^, which could possibly mediate the myosin II assembly we observe in the bleb rear to pull actin rearward and disassemble it at the bleb neck^20,28^. This spatial reorganization of Rho GTPases and PI3K signalling^69^ may enable cancer cells to use the same basic motility building blocks to rapidly shift from mesenchymal to bleb-based migration without the need for activating different transcriptional programs, thus quickly suiting the migratory mode to varying environments encountered on the metastatic journey.

Our study identifies CD44 as a key regulator for maintaining polarity and bleb stability in metastatic melanoma cells, in line with its high expression at tumour fronts^70^. CD44 is known to function both as a biophysical regulator of plasma membrane receptor activity and as a signalling receptor itself through binding hyaluronic acid. Our findings suggest that during bleb-based migration, CD44’s primary role is biophysical, acting as a transmembrane ‘picket’, to corral EGFR via an ERM protein and actin meshwork at the bleb rear, where lower receptor density at the front limits activation^71,72^. This configuration could form a feedback loop as growth factor signalling promotes downstream ERM protein phosphorylation, linking CD44 to actin. Although hyaluronan degradation did not alter EGFR polarity in our experiments, this mechanism might vary in the tumour microenvironment. Moreover, our preliminary data show EGFR and CD44 gradient in the large bleb in 3D under more physiological conditions. Recent work has shown that this pathway is conserved in 3D, where upstream PI3K and Rac activity is restricted to bleb base along with other potential picket proteins such as septin^69,73^. Our data reveal that CD44 restricts EGFR diffusion across the bleb, affecting its spatial distribution and contributing to polarized signalling.

The spontaneous polarization of metastatic melanoma cells in the absence of chemical or haptotactic gradients, coupled with their efficient migration in confined, low-adhesion environments, has important implications for tumour invasion and metastasis. Even in complex environments with ECM ligands present, cancer cells navigating high confined spaces are able to switch to pressure based motility^5,68,74^. Melanoma cells have high ERK and MAPK signalling due to BRAF mutation conferring high contractility that increases the invasive potential of cancer cells, confer anoikis resistance^73,75^ and promote drug resistance^68^ possibly by EGFR signalling at the back of the bleb. The effect of confinement on these signalling pathways, how common these phenomena are across different solid tumours and how they change with disease progression remain important areas for further exploration.

## Methods

### Cell culture

A375M A2 cells were obtained from American Type Culture Collection (ATCC CRL-3223) and were maintained at 37 °C with 5% CO_2_ for fewer than 15 passages in tissue culture treated dishes (Falcon). Cells were cultured in DMEM supplemented with 10% FBS (Atlanta Biologicals), GlutaMAX (Gibco) and 20 mM Hepes (pH 7.4). Transient transfection of cDNAs or siRNAs were performed using the Amaxa nucleofector kit V (Lonza) with 1.5 µg of DNA for 2 × 10^6^ cells, or with 1 µM of siRNA for 48 h prior to experimental use. For drug treatments or siRNA or cDNA transfections, cells were plated on 6 well pre-treated tissue culture plates (Nunc 6 well multiwell plate, Thermo fischer scientific).

For serum starvation, cells were plated on 6 well tissue culture plates for 2 h and then the culture medium was replaced with DMEM without FBS for 12 h. Cells were trypsinized, pelleted and resuspended in fresh FBS-deprived medium. For EGF stimulation in serum-starved cells, medium was supplemented with 50 ng ml^−1^ EGF (Thermo Scientific, PHG0311L).

For 3D collagen ECM cultures, A375M A2 cells were embedded in 2.5 mg ml^−1^ type I collagen containing Alexa 647-labelled and unlabelled collagen (1:1 ratio). Plasma-cleaned 35 mm glass-bottom dishes were coated with 300 µl of 1.5 mg ml^−1^ collagen containing 3,000 cells and allowed to polymerize at 37 °C for 1 h. An additional 500 µl of 1.5 mg ml^−1^ unlabelled collagen was layered on top and polymerized for a further 1 h. After polymerization, 1 ml of culture medium was gently added, and cells were incubated overnight at 37 °C. Fresh medium was added 1 h prior to imaging or fixation.

### cDNA expression vectors and lentiviral expression

The following cDNAs were used for DNA transfections: EGFR-GFP (Addgene 32751), Akt-PH-GFP (kind gift from T. Balla), GFP-C1-PLCδ-PH (Addgene 21179), Pak-GBD-YFP and Rtkn-RBD-GFP (gifts from K. Hahn), pTriEX-PA-Rac1(C450A) (Addgene 22025), pTriEx-PA-Rac1 (Addgene 22024), FR-F-tractin (gift from M. Baird (from M. Davidson collection)), CD44-GFP (gift from S. Mayor)^72^, GPI–GFP, Gt46-GFP, LAT–GFP and CAAX–GFP (gifts from J. Lippincott-Schwartz)^57^, C1-MPAct-mCitrine (Addgene 155220)^58^. EGFR-FR, Myr-FR-Cry2Drop-EGFR and Clover-ZtSH2 and EGFR-CD3ε-FR are described in refs. ^45,49^. For siRNA-based knockdowns, ON-TARGET plus SMART pool siRNAs (Dharmacon) were used for human *CD44* (L-009999-00-0005), ezrin (L-017370-00-0005), moesin (L-011732-00-0005), siGENOME siRNA Human *EGFR* (D-003114-34-0005, referred as *EGFR*-siRNA1) and predesigned Human siRNA *EGFR* (s565 from Invitrogen/Thermo Scientific, referred as *EGFR*-siRNA2) were used. For CD44 rescue, two independent siGENOME 3′ UTR human *CD44* siRNAs were utilized (Dharmacon, D-009999-20-0005 and D-009999-21-0005).

### Lentivirus

Lentiviral particles were generated by transfecting HEK293FT (~1 million cells, from ATCC, CRL-3216) cultured in DMEM with Lipofectamine 2000 (Thermo Fisher) according to manufacturer’s protocols using 2.5 µg plasmid DNA of interest, 1 µg pMD2.G, and 2.5 µg psPax2, and collecting the virus-containing supernatant at 48 h in medium without FBS. Supernatant was filtered through a 0.45-µm filter (Sigma), then immediately placed on target A375M A2 cells that had been seeded 24 h before transduction at a 1:1 ratio of viral supernatant to supplemented DMEM medium and allowed to incubate for 48 h, after which cells were passaged into medium containing a final concentration of 2.0 µg ml^−1^ puromycin for selection. Stable cell lines were generated with the following constructs: EGFR–FR, Myr-FR-Cry2Drop-EGFR, Clover-ZtSH2 and EGFR–CD3ε–FR^45,49^, selected with 2.0 µg ml^−1^ puromycin, and then expanded for 2 weeks or more (to reach around 80 percent confluency), followed by sorting by flow cytometry for Clover- and FR-positive cells of medium fluorescence intensity level.

### Drug treatments

Stock concentration of erlotinib 10 µM (Sigma-Aldrich, SML2156), lapatinib 50 µM (Sigma-Aldrich), gefitinib 50 µM (Sigma-Aldrich), wortmannin 10 µM (Sigma-Aldrich, W3144) and jasplakinolide 100 nM (Invitrogen), hyaluronidase 20 units ml^−1^ from bovine testes (Sigma-Aldrich). Working concentrations were prepared by mixing these with the cell culture medium. Working concentrations are mentioned throughout the manuscript.

### Cell confinement assays

#### Dynamic confiner

To subject the cells to confinement for high-resolution live cell imaging, the 1-well Dynamic Cell Confiner System (4Dcell) was used per manufacturer’s directions^76^. The device consists of a pressure cup, a confiner coverslip affixed with micropillars, and a glass-bottom dish^20^. The height of the micropillars determined the height for the spatial confinement of the cells between the coverslip and the substrate, which was 3 μm in this study. The confiner coverslips were sonicated in PBS, air dried under the hood. The glass-bottom dishes (Fluorodish, 35 mm, FD3510-100) and the confiner coverslips were plasma-treated for 1 min, incubated with 0.5 mg ml^−1^ pLL-*g*-PEG (SuSoS, PLL(20)-g[3.5]-PEG(2)) in 10 mM pH 7.4 HEPES buffer for 1 h at room temperature. Confiner coverslips and glass-bottom dishes were rinsed briefly with PBS and incubated in cell culture medium for at least 2 h before confining the cells. Prior to confinement, cells were trypsinized (Tyrpsin-EDTA 0.25%), washed, and collected in a pellet by brief centrifugation at 2,000*g* and resuspended in warm DMEM with FBS (without phenol red) and then plated on glass-bottom dishes that were pre-treated with PLL-g-PEG and incubated at 37 °C for 30 min and was then placed on the microscope. The confiner coverslip with pillars, attached to the confiner suction cup, was then lowered over the sample and attached with low pressure seal sufficient for attachment to the glass dish but insufficient for confinement of the cells. Confinement was then initiated slowly by increasing the pressure utilizing the custom software (4Dcell) to control the vacuum pump.

#### Agarose pads

Cell confinement for immunostaining was performed under agarose slabs. Agarose slabs for cell confinement^18^ were made by boiling 750 mg of ultrapure agarose (Life Technologies) added to 50 ml of DMEM without phenol red and pouring 1 ml directly in a fluorodish (35 mm, FD3510-100). After gelation, a hole was punched in the agarose using a small biopsy needle. Prior to confining, 1 ml of 1% BSA made in DMEM was added to the agarose slabs and was left to equilibrate and coat overnight. Before use, medium with BSA was thoroughly aspirated and 100 µl of medium containing cells was pipetted below the agarose pad by gently lifting the pad slightly from the empty hole. The remaining medium was thoroughly vacuumed out of the punch hole. To prevent drying, the dish was sealed using parafilm and was incubated for 1 h in the incubator. After 1 h, immunostaining was performed using the protocol described below.

### Immunofluorescence

Samples under agarose pad confinement were fixed with 4% paraformaldehyde (PFA; Electron Microscopy Sciences, 15710) in cytoskeleton buffer (10 mM MES, 3 mM MgCl_2_, 138 mM KCl, 2 mM EGTA) for 20 min at 37 °C, after which the agarose pad was gently removed. Cells were permeabilized with 0.5% Triton X-100 in cytoskeleton buffer for 5 min at room temperature and quenched with 10 mM glycine in cytoskeleton buffer. Samples were washed with Tris-buffered saline (TBS; 20 mM Tris, pH 7.6, 137 mM NaCl) (2 × 5 min, then 2 × 10 min) and blocked for 1 h at room temperature in blocking buffer (2% IgG-free and protease-free BSA and 0.1% Tween-20 in TBS).

Cells were incubated for 2 h at room temperature or overnight at 4 °C with primary antibodies against CD44 (BJ18; BioLegend), ezrin (3C12; Invitrogen), EGFR (H11; Invitrogen) or phospho-ERM (Thr567/564/558; Cell Signaling Technology) diluted in TBS. Following 3 TBS washes, samples were incubated for 1 h at room temperature with fluorophore-conjugated secondary antibodies (1:500; Jackson ImmunoResearch) and Alexa Fluor 647–phalloidin (1:200; Thermo Fisher Scientific) diluted in blocking buffer. Samples were washed with TBS (2 × 10 min) and mounted in Dako mounting medium (S3023) on glass-bottom dishes.

For cells embedded in collagen, samples in 35 mm glass-bottom dishes were fixed with 4% paraformaldehyde in cytoskeleton buffer (10 mM MES, pH 6.1, 138 mM KCl, 3 mM MgCl_2_, 2 mM EGTA) for 60 min at 37 °C, followed by permeabilization with 0.5% Triton X-100 in cytoskeleton buffer for 3 h at room temperature. Samples were washed with TBS-T (0.1% Tween-20; 3 × 15 min) and TBS (3 × 10 min), then blocked overnight at 4 °C. Primary antibody staining was performed overnight at 4 °C with anti-CD44 (BJ18; BioLegend) and anti-EGFR (H11; Invitrogen) in TBS. After washing, samples were incubated with fluorophore-conjugated secondary antibodies (1:500; Jackson ImmunoResearch) for 3 h at room temperature, washed with TBS (3 × 10 min), and imaged.

### Immunoblotting

Cells were lysed in 2× SDS sample buffer, separated by SDS–PAGE (4–20% Tris-Glycine gels (Thermo Fisher Scientific)) and electro-transferred at room temperature for 1 h to Immobilon-P PVDF membrane. Membranes were blocked for 1 h at room temperature with 5% nonfat dry milk in TBS-T buffer (TBS + 0.1% Tween-20) then incubated for 2 h at room temperature with indicated primary antibodies. Subsequently, membranes were washed 3 × 5 min in TBS-T, incubated with appropriate horseradish peroxidase (HRP)-conjugated secondary antibodies (1:10,000) for 1 h at room temperature then washed 3 × 5 min in TBS-T. An ECL detection system (iBright 1500, Invitrogen) was used to visualize protein bands. The following antibodies were used: anti-phospho-EGFR (1:1,000; Tyr1068, D7A5 Cell Signaling Technology), anti-EGFR (1:1,000; D381B1, Cell Signaling Technology), anti-CD44 (BJ18, Biolegend), anti-phospho-AKT (1:2,000; Ser473, D9E, Cell Signaling Technology), anti-ezrin (3145, Cell Signaling Technology) and anti-GAPDH (14C10, Cell Signaling Technology). The secondary antibodies HRP-conjugated goat anti-mouse (115-035-003) or goat anti-rabbit (111-035-003) were from Jackson ImmunoResearch Laboratories.

### Spinning disk confocal imaging

Confocal fluorescence imaging was performed on a Nikon Eclipse Ti2 microscope equipped with a Yokogawa CSU-W1 spinning disk scan-head along with Perfect FocusTM (Nikon). Either a Nikon Plan Apo 60× oil 1.49 NA DIC or Nikon Plan Apo 20×/0.75 Ph2 DM objective lens was used. Illumination was provided by a Nikon LUNV 6-line laser unit, and images were captured with a Hamamatsu Orca-Flash 4.0 v3 camera. Phase-contrast illumination was provided by a 100 W halogen bulb or LED using an 0.52 NA condenser lens. Microscopes were equipped with the Nikon motorized stage with *xy* linear encoders and a Mad City (Madison, WI) Nano-Z100 piezo insert with 200um travel. Laser confocal or transmitted illumination were selected with electronic shutters and an automated filter turret containing a multi-bandpass dichromatic mirror together with an electronic emission filter wheel. Microscope functions were controlled by NIS-Elements software (Nikon). Cells were imaged in 35 mm glass-bottom dishes (FluoroDish) or the 1-well Dynamic Cell Confiner System (4Dcell), depending on experimental conditions.

### Laser scanning confocal imaging

FRAP was performed on an inverted Nikon A1R resonance-scanning confocal microscope on a Nikon Ti body with perfect focus system and a motorized stage with *xy* encoder and Mad City piezo stage insert. Images were acquired with a Nikon Apo 60×/1.4 oil λS DIC N2 objective lens and controlled by NIS-Elements software (with advanced research package). For photobleaching experiments, either the 488 nm or 405 nm lasers in the Nikon LU-n4 laser combiner was used to photobleached using the Galvanometric scanner, and the same scanner was used for image acquisition.

### Super-resolution confocal imaging

Imaging of confined live and fixed cells for super-resolution imaging was performed using a Zeiss LSM880 with the Axio Observer7 confocal microscope stand equipped with Airyscan and a piezo *z* stage (Wienecke & Sinske GmrH), and was controlled by ZenBlack software (Zeiss), Images were acquired using a Plan Apo 63× 1.4 NA DIC M27 oil objective with a 32-channel GaAsP-PMT area detector. Airyscan image reconstructions were processed in auto strength mode using ZenBlack software (Version 2.3). 488 nm multi-line 25 mW Argon laser, 561 DPss solid state 15 mW laser and 633 nm HeNe 5 mW lasers were used for illumination. For optogenetics experiments, the bleaching sub-module with the 488 nm laser was used (using 10-20% laser power) and images were acquired using multidimensional acquisition module of the Zeiss software. Additional analysis was performed in ImageJ (NIH).

### Image analysis

#### Cell migration analysis

Cell migration under confinement was analysed from time-lapse phase-contrast images acquired every 1 min for 50–60 min. Cell trajectories were manually tracked in Fiji using the Manual Tracking plug-in (MTrackJ^77^), by following the cell body centroid. The tracking coordinates were exported to Diper^78^ (Microsoft Excel plug-in), to compute mean squared displacement over time and speed using overlapping time intervals (Supplementary Note 1). MSD was computed for a given cell for step size *n* with *N* being the total number of displacements per trajectory and Δ*t* the minimum time interval between the adjacent points in the trajectory. MSD for step size *n* was calculated for each trajectory and fitted to the persistent random walk model using Furth’s equation:

$${MSD}=4D\left[\Delta t-P\left(1-\exp \left(\frac{-\Delta t}{P}\right)\right)\right]$$

where *D* is the diffusion coefficient and *P* is the persistence time. Nonlinear fitting was performed in Origin (Pro) using the Levenberg–Marquardt algorithm, and only fits with *R*^2^ ≈ 1 were accepted. Persistence time and diffusion coefficients were plotted in GraphPad Prism.

#### Bleb distribution and lifetime

Blebbing cells were classified from time-lapse images based on bleb lifetime, defined as the duration of one bleb cycle (nucleation, expansion, persistence, and retraction). Spherical blebs with lifetimes <1 min were categorized as transient blebs. Cylindrical blebs with lifetimes >1 min were classified as elongated blebs. Elongated bleb length was measured at each time point using segmented line regions of interest (ROIs) in Fiji. Cells were further categorized as having multiple elongated blebs, dynamic elongated blebs (<25 min lifetime), or leader blebs (>25 min lifetime).

#### Normalized intensity profiles

Fluorescence intensity profiles were measured using a segmented line ROI (10-pixel width) drawn from the base to the tip of each bleb. Intensities were normalized to the maximum value and divided into 11 bins (0, base; 1, tip). Mean normalized intensity profiles were averaged across blebs and plotted.

#### Polarity index

Polarity index in elongated blebs was defined as PI = (*I*_front_ − *I*_rear_)/(*I*_front_ + *I*_rear_) where *I*_front_ is the integrated intensity of fluorescently tagged membrane proteins at the bleb tip (at bin 1 of the bleb length) and *I*_rear_ is intensity at the bleb base (at bin 0 of the bleb length).

#### Analysis of EGFR sensor

Intensity profiles from EGFR–CD3ε–FR (EGFR–FR) and Clover-ZtSH2 (EGFR sensor) were calculated using a segmented line function in Fiji by drawing an ROI of 10-point width from the base to the tip of the bleb. Measured intensity was normalized to the maximum intensity and was divided as a function of bleb length into 11 bins, taking 0 for the bleb base and 1 for bleb tip. The ratio of the intensity of EGFR sensor to EGFR was calculated for each bin and plotted in a similar fashion. Normalized mean intensity profiles from all the blebs were averaged for each position bin and plotted. Polarity indices of EGFR sensor, EGFR–FR and ratio were computed and plotted as described above.

#### Bleb aspect ratio

Aspect ratio was calculated as the ratio of bleb length to maximum width, measured using segmented line ROIs in Fiji.

#### Length of bleb pre- and post-photoactivation

For EGFR activation in the tips of the blebs, a rectangular ROI was selected for photoactivation in the blebs of the cells expressing Myr-FR–Cry2Drop–EGFR (Opto-EGFR–FR) or EGFR–FR. ROIs and the size of ROI were selected such that it covered the tip of the bleb and some additional space for bleb movement. Continuous activation using 488 nm laser was performed in this defined region with image acquisition approximately every 3 s for around 25 min. For Rac activation experiments, a circular ROI of diameter ~15 µm was selected for defining the region of activation in blebs in the cells co-expressing PA-Rac1 or PA-Rac1(C450A) with FR–F-tractin. Slight differences in the ROI diameter was used based on the size of the tip of the bleb. Continuous activation using 488 nm laser was performed with image acquisition every ~1 s for 5 min to capture changes in the actin dynamics and architecture. To measure the bleb length pre- and post-photoactivation, a segmented line function was used in Fiji (ImageJ) to a draw line from the base of the bleb to the tip and values corresponding to the length of the bleb were extracted and exported to graph.

#### Actin coherency measurement

Actin coherency was measured in the cells expressing FR–F-tractin by using the OrientationJ macro in Fiji (ImageJ) that derives local orientation and isotropic values (coherency and energy) of every pixel. Coherency across the leader bleb was measured by dividing the bleb into 20 equal oval regions of interest (ROIs) slightly overlapping each other from the rear to the tip of the bleb. The area of the ROIs were based on the area of the leader bleb. The coherency from each ROI was extracted and average was computed and plotted for many blebs.

### FRAP

#### Spot FRAP and analysis

For spot FRAP experiments, the intensity recovery over time was extracted from small area of ~1 μm diameter where photobleaching was performed. Intensity data was extracted from this region pre- and post-bleaching. Two other similar sized ROIs were used to extract intensities from the background of the image and another region in an unbleached area of the cell used for bleaching control. The extracted intensities from each ROI was then uploaded in EasyFRAP-web FRAP analysis tool^79^. Intensity values were normalized and fit with a single-term exponential equation. The web tool computes *t*_1/2_ (half-time of maximal recovery) for each of the of the recovery profiles and plots for different conditions.

EGFR diffusion coefficient was computed by averaging the normalized recovery intensity and the average was fitted with one phase association, least squares fit to compute *t*_1/2_

$$y\left(t\right)=A\times \left(1-\exp \left(-K\times {t}_{1/2}\right)\right)$$

*t*_1/2_ was used to calculate the diffusion coefficient of EGFR–GFP on the membrane using Soumpasis equation^80,81^ and *K* was the rate constant.

$$D=0.224{r}^{2}/{t}_{1/2}$$

Where *D* is the diffusion coefficient, *r* is the radius of the circular FRAP area and *t*_1/2_ is half maximal recovery time.

#### Rectangular FRAP and flow analysis

A rectangular area spanning the width of the leader bleb was photobleached in the rear quadrant of the bleb and intensity recovery was analysed by drawing a line perpendicular to the long axis of the rectangle in Fiji. The intensity line scans were measured over time and each was fitted with an inverse nonlinear gaussian curve fit using a Gaussian amplitude (GaussAMp) function in Origin Lab to find the minima of the fitted inverse gaussian, where the function is given by

$$y-{y}_{0}+A{{\rm{e}}}^{-{\left(x-{x}_{{\rm{C}}}\right)}^{2}/2{\omega }^{2}}$$

where *x*_C_ = centre of the peak, *y*_0_ = offset, *w* = width, *A* = amplitude of the gaussian; the position of the centre of the minima of the gaussian was tracked in the images taken in the first few seconds of intensity recovery and then averaged across several blebs.

#### Whole-bleb FRAP and recovery analysis

An area corresponding to the whole bleb was photobleached and the intensity recovery was analysed by drawing a ROI line segment across the bleb spanning from the rear to the tip of the leader bleb. For analysis of the intensity recovery rate constant, a small circular ROI of 1 µm was drawn close to the neck of the bleb and the recovery intensities were recorded. The extracted intensities from each ROI was then uploaded in EasyFRAP-web FRAP analysis tool^79^. Intensity values were normalized, and the rate constant was computed from the average by fitting with one phase association, least squares fit to compute *K* as above.

#### Characteristic bleb length and width measurements

To measure the characteristic length of the bleb, a line segment was drawn from the base to the tip of the bleb in Fiji (ImageJ) and length was extracted and average of several such leader-bleb lengths was taken as characteristic bleb length. For the characteristic width of the bleb, each bleb was divided into 10 equal reference points and a line segment was drawn at each point spanning across the width of the bleb and this value was extracted for the width at each of the reference points for several blebs. An average of the width across each of the reference points were reported as a characteristic width measurement.

#### Actin PIV analysis

The motion of the F-tractin–FR-labelled actin network was determined in consecutive frames ~6 s apart in PIVLab^82^ MATLAB (Mathworks). The background and the cell body were excluded from the analysis by manually masking it out. Vector velocities were used to calculate magnitudes in the leader bleb.

#### Bleb elongation measurement and heat map

As the bleb protruded and elongated, the intensity measurements of either EGFR–GFP or CD44–GFP were made by drawing a line segment in Fiji (ImageJ) from the rear to the front of the bleb manually. Intensity measurements were extracted and the heat map was plotted in Origin lab.

### Mathematical modelling and solutions

#### Membrane protein flux and material balance

We consider that our membrane protein (density *P*) is subject to diffusion, advection, and turnover (removal by endocytosis or degradation) in the bleb (see Supplementary text 2). Based on evidence, we assume that all synthesis of the protein occurs in the cell body, and therefore there is a net transport of protein from the cell body into the bleb. The apparent diffusivity (*D*_p_), the advection velocity (*V*_p_), and turnover frequency (*k*_p_) of the protein potentially vary with position *x* in the bleb, measured from the neck of the bleb. In the material balances, we also need to account for the geometry of the bleb; Its length *L* (set to 45 μm), and position-dependent width *w*(*x*) (see below), were estimated from mean measurements. Flux (*N*_p_) and steady-state material balance equations for the protein may thus be written as

$${N}_{p}\left(x\right)=-{D}_{p}\frac{dP}{dx}+{V}_{p}P;$$

$$0=-\frac{{\rm{d}}}{dx}\left({N}_{{\rm{p}}}w\right)-{k}_{{\rm{p}}}{Pw}.$$

The spatial dimension *x* is defined as the distance from the neck of the bleb (μm); *x* = *L* is at the tip of the bleb. *N*_p_ and *V*_p_ and point in the direction of *x* by convention, and therefore a positive or negative value indicates net flux or flow towards the tip or neck, respectively. Zero net flux at the tip dictates the boundary condition,

$${N}_{{\rm{p}}}\left(L\right)=0.$$

The calculated protein density profiles are presented on a normalized scale, and therefore the second boundary condition is arbitrary.

#### Protein advection velocity, diffusivity and turnover

To further define the aforementioned *V*_p_(*x*) and *k*_p_(*x*), it was necessary to consider the material balance of the bulk membrane. Defining *V*_m_(*x*) and *k*_m_(*x*) as the flow velocity and turnover frequency of bulk membrane, respectively, and with the ansatz that the total mass density is constant throughout the bleb (confirmed by experiment),

$$0=-\frac{{\rm{d}}}{{\rm{d}}x}\left({V}_{{\rm{m}}}w\right)-{k}_{{\rm{m}}}w.$$

If the bleb has a constant shape, it implies that there is zero flow at the tip (*V*_m_(*L*) = 0). This boundary condition dictates that *V*_m_(*x*) ≥ 0; membrane flow is towards the tip of the bleb. For the calculations presented here, we took *k*_m_(*x*) to be constant and set its value to 0.003 s^−1^, thus approximately matching the estimated bulk flow velocity of *V*_m_ ≈ 0.1 μm s^−1^ measured at *x* ≈ 10 μm. Relative to the calculated *V*_m_(*x*), we consider that *V*_p_(*x*) is potentially influenced by two distinct, protein-corralling effects: restriction of free protein mobility, and physical interaction with the F-actin retrograde flow. Defining *V*_F_(*x*) as the local velocity of the F-actin, we take

$${V}_{{\rm{p}}}\left(x\right)=\left(1-{\varepsilon }_{1}\right)\left(1-{\varepsilon }_{2}\right){V}_{{\rm{m}}}+{\varepsilon }_{2}{V}_{{\rm{F}}},$$

where ${\varepsilon }_{1}(x)$ are ${\varepsilon }_{2}(x)$ are variables, each ranging between 0 and 1, which we refer to as corralling efficiencies. Their values reflect the ‘strengths’ of protein mobility restriction and interaction with flowing F-actin, respectively. The function, *V*_p_(*x*) = −0.10 + 0.08e^−0.2x^ (μm s^−1^), was used to approximate the experimentally estimated profile from PIV analysis. Regarding the protein diffusivity, it is reduced by the corralling effects as well, and so we likewise take

$${D}_{{\rm{p}}}\left(x\right)=\left(1-{\varepsilon }_{1}\right)\left(1-{\varepsilon }_{2}\right){D}_{{\rm{p}}0},$$

where *D*_p0_ is the diffusivity value in the absence of corralling; for each scenario, the value of *D*_p0_ was set to approximately match the estimated EGFR diffusivity *D*_p_ ≈ 0.02 μm^2^ s^−1^ measured at *x* ≈ 10 μm. Considering the potential for spatially varying *ε*_*i*_(*x*) (with *i* = 1 or 2) in a simple manner, we allow each function to be characterized by its value evaluated at the neck (*x* = 0), *ε*_*i,0*_, and another parameter, *λ*_*i*_, the length scale of its gradient:

$${\varepsilon }_{i}\left(x\right)={\varepsilon }_{i,0}\frac{\cosh \left[\left(L-x\right)/{\lambda }_{i}\right]}{\cosh \left(L/{\lambda }_{i}\right)}.$$

In the limit of ${\lambda }_{i}\to \infty$, ${\varepsilon }_{i}\left(x\right)\approx {\varepsilon }_{i,0}$ (constant). Finally, to model membrane protein turnover frequency in the simplest manner, consistent with observations, we equated it to that of the bulk membrane.

$${k}_{{\rm{p}}}={k}_{{\rm{m}}}.$$

##### Solution scheme

The scheme for solving the model equations was to transform them into a set of coupled, first-order differential equations:

$$\frac{d{N}_{p}}{d\hat{x}}=-\left(\frac{dw/d\hat{x}}{w}\right){N}_{p}+\left({k}_{p}L\right)P;$$

$$\frac{{dP}}{{\rm{d}}\hat{x}}=\frac{L}{{D}_{{\rm{p}}}}\left({N}_{{\rm{p}}}-{V}_{{\rm{p}}}P\right);$$

$$\frac{{\rm{d}}{V}_{{\rm{m}}}}{{\rm{d}}\hat{x}}=-\left(\frac{{\rm{d}}w/{\rm{d}}\hat{x}}{w}\right){V}_{{\rm{m}}}+{k}_{{\rm{m}}}L,$$

where

$$\hat{x}=1-\frac{x}{L};$$

$$w\left(\hat{x}\right)\,=-117.82{\hat{x}}^{4}+213.92{\hat{x}}^{3}-137.88{\hat{x}}^{2}+39.833\hat{x}+6.079.$$

The latter equation, was fit to the mean of 17 representative width profiles (μm). This scheme permitted forward integration, with boundary conditions

$${\left.{N}_{p}\right|}_{\hat{x}=0}=0;\,\,\,\,\,\,{\left.P\right|}_{\hat{x}=0}=1;\,\,\,\,\,\,{\left.{V}_{m}\right|}_{\hat{x}=0}=0.$$

The domain was discretized with a spatial step size of 0.001, and the initial value problem was solved using the improved Euler method/Heun’s method, implemented in Excel for rapid evaluation of the model output and analysis of numerical accuracy and stability. Once the solution for each scenario was obtained, the $P(x)$ profile was normalized by the maximum value for presentation in figures.

### Statistics and reproducibility

Statistical analyses were performed using GraphPad Prism. Data are presented as mean ± s.d. from at least three independent experiments (*N*), as indicated in the figure legends. No statistical methods were used to pre-determine sample sizes but our sample sizes are in line with the field^20^. Reproducibility was determined based on at least three individual repeats. Data distribution was assumed to be normal but this was not formally tested. Statistical significance was assessed using Student’s *t*-test (Welch’s correction), Kolmogorov–Smirnov test, or Mann–Whitney test, as specified in the figure legends. For persistence analyses, Welch’s *t*-test was applied to individual cell data (*n* represents number of cells). For violin plots of leader-bleb speed, statistical significance was calculated using the means of biological replicates due to large *n* values. Experiments in Fig. 5j,l,n and Extended Data Figs. 1l–n and 4a,c,d,f were performed at least three times independently with similar results. Acquisition of images of samples were performed in random. Data collection and analysis were not performed blind to the conditions of the experiments. No data points were excluded from the analysis. Significance levels are reported in the figure legends, with *P* > 0.05 considered not significant.

#### Scientific illustrations

Illustrations and cartoons were made using Adobe illustrator and BioRender.

### Reporting summary

Further information on research design is available in the Nature Portfolio Reporting Summary linked to this article.

## Online content

Any methods, additional references, Nature Portfolio reporting summaries, source data, extended data, supplementary information, acknowledgements, peer review information; details of author contributions and competing interests; and statements of data and code availability are available at 10.1038/s41556-026-01981-1.

## Supplementary information

Supplementary Information Supplementary note and Supplementary Fig. 1.

Reporting Summary

Peer Review File

Supplementary Video 1 **Cells show different bleb phenotypes under confinement**. Four different bleb phenotypes that A375M2 cells achieve under 3-μm confinement and low-adhesive substrate (PLL-*g*-PEG). The video shows small transient blebs (top left), multiple elongated blebs (top right), dynamic elongated blebs (bottom left) and leader blebs (bottom right). Scale bar 25 μm, elapsed time shown.

Supplementary Video 2 **Growth factor signalling is required for leader bleb formation and migration**. A375M2 cells under 3-μm confinement and low-adhesive substrate (PLL-*g*-PEG) after serum starvation (middle) and stimulation with EGF (right) compared to FBS-containing medium control. Scale bar 25 μm, elapsed time shown.

Supplementary Video 3 **PI3K and EGFR activities are required for leader bleb formation and migration**. A375M2 cells under 3-μm confinement and low-adhesive substrate (PLL-*g*-PEG) after pre-treatment with Wortmannin (PI3K inhibition) and Erlotinib, Lapatinib, Gefitinib (EGFR kinase inhibition) compared to DMSO control. Scale bar 25 μm, elapsed time shown.

Supplementary Video 4 **EGFR is required for leader bleb formation and migration**. A375M2 cells under 3-μm confinement and low-adhesive substrate (PLL-*g*-PEG) after transfection with EGFR siRNA 1 compared to non-targeting siRNA 1 (control) (top panel) or EGFR siRNA 2 compared to non-targeting siRNA 2 (control, bottom panel). Scale bar 25 μm, elapsed time shown.

Supplementary Video 5 **Mislocalization of Rac activity to the tip of the leader blebs stalls bleb elongation**. A375M2 cells under 3-μm confinement and low-adhesive substrate (PLL-*g*-PEG) expressing either FR–F-Tractin to mark actin filaments and either PA-RAC1 (Photoactivable RAC1) or PA-Rac1(C450A) (Control, Rac1-GTPase dead). Continuous 488 nm activation was done on the bleb tips (regions marked with white circles). Scale bar 10 μm, elapsed time shown.

Supplementary Video 6 **EGFR flows slowly from the cell body to the leader bleb**. A375M2 cells under 3-μm confinement and low-adhesive substrate (PLL-*g*-PEG) expressing EGFR–GFP, where the whole bleb is photobleached (outlined area) and recovery is imaged over time. Scale bar 10 μm, elapsed time shown.

Supplementary Video 7 **Leader blebs have fast actin retrograde flow**. A375M2 cells under 3-μm confinement and low adhesive substrate (PLL-*g*-PEG) expressing FR–F-tractin. Scale bar 20 μm, elapsed time shown.

Supplementary Video 8 **Actin retrograde flow is dispensable for EGFR distribution in leader blebs**. A375M2 cells under 3-μm confinement and low adhesive substrate (PLL-*g*-PEG) co-expressing FR–F-tractin and EGFR–GFP, pre-treated with DMSO (Control) or Jasplakinolide. Scale bar 10 μm, elapsed time shown.

Supplementary Video 9 **EGFR and a membrane marker exhibit anterograde flow in leader blebs**. A375M2 cells under 3-μm confinement and low adhesive substrate (PLL-*g*-PEG) expressing either EGFR–GFP or CAAX–eGFP where a rectangular area across the width of the bleb and close to the neck is photobleached and the direction of recovery is monitored. Scale bar 5 μm, elapsed time shown.

Supplementary Video 10 **EGFR is restricted at the bleb base during leader bleb formation**.A375M2 cells under 3-μm confinement and low adhesive substrate (PLL-*g*-PEG) expressing EGFR–GFP. The video shows the dynamics of EGFR–GFP during bleb initiation and elongation. Scale bar 10 μm, elapsed time shown.

Supplementary Video 11 **EGFR mobility is restricted at the base of the bleb**. A375M2 cells under 3-μm confinement and low adhesive substrate (PLL-*g*-PEG) expressing EGFR–GFP where small areas (white circles) are photobleached in the base and the tip of the bleb simultaneously. Scale bar 10 μm, elapsed time shown.

Supplementary Video 12 **CD44 restricts EGFR mobility at the base of the bleb**. A375M2 cells under 3-μm confinement and low adhesive substrate (PLL-*g*-PEG) expressing EGFR–GFP where a small area is photobleached in the bleb base in cells transfected with either scramble-siRNA (control) or pooled CD44-siRNA. Scale bar 10 μm, elapsed time shown.

Supplementary Video 13 **CD44 is restricted at the bleb base during leader bleb formation**. A375M2 cells under 3 μm confinement and low adhesive substrate (PLL-*g*-PEG) expressing CD44–GFP. The video shows the dynamics of CD44–GFP during bleb initiation and elongation. Scale bar 10 μm, elapsed time shown.

Supplementary Video 14 **CD44 is required for leader bleb based migration**. A375M2 cells under 3-μm confinement and low adhesive substrate (PLL-*g*-PEG) transfected with control siRNA (Control, top left), two different CD44 siRNA or s (targeting the 3′ UTR, top middle and right) control siRNA together with CD44–GFP (bottom right) or two different CD44 siRNA or s (targeting the 3′ UTR) together with CD44–GFP (bottom middle and right). Scale bar 25-μm, elapsed time shown.

## Source data

Source Data Fig. 1 All the statistical source data for all the figures and extended figures.

Source Data Fig. 2 Unprocessed western blots.

## Data Availability

All data needed to evaluate the conclusions are provided in the main text and figures, extended data figures or supplementary text. All the unprocessed immunoblots or raw data and associated statistical calculations are provided with this study. Source data are provided with this paper with data points and statistical analysis. All other data supporting the findings of this study are available from the corresponding author on reasonable request. Source data are provided with this paper.

## Code availability

Mathematical algorithms used are provided in the methods and in the supplementary text.
