# Supplementary material for: CD44 restricts EGFR mobility to polarize cytoskeletal signalling modules driving bleb-based migration
Source: Nat Cell Biol. 2026 Jul 6;28(7):1408–23. doi: 10.1038/s41556-026-01981-1 (PMC13364714; doi:10.1038/s41556-026-01981-1)
Supplement: Supplementary file 1 — Supplementary note and Supplementary Fig. 1. [file 41556_2026_1981_MOESM1_ESM.pdf]

nature cell biology

Supplementary information https://doi.org/10.1038/s41556-026-01981-1

CD44 restricts EGFR mobility to polarize cytoskeletal signalling modules driving bleb-based migration

In the format provided by the authors and unedited

Supplementary information

1) Cell migration analysis (detailed)

Migration analysis of the cells under low-adhesion confinement was performed on time-lapse phase contrast image series (acquired every 1min for 60 min) in Fiji using "Manual Tracking plug-in" (MTrackJ [1]), where each step was manually tracked following the center of the cell body. The tracking coordinates were then exported to the Microsoft Excel plug-in, Diper[2], and mean squared displacement over time and speed were computed according to:

$$MSD\ (n) = \frac{1}{N-n+1} \sum_{1=0}^{N:n} \left[\left(x_{(i+n)\Delta t} - x_{i\Delta t} \right)^2 + \left(y_{(i+n)\Delta t} - y_{i\Delta t} \right)^2 \right]$$

by utilizing overlapping time intervals. MSD is computed for a given cell for step size *n* with N being the total number of displacements per trajectory and Δt is the minimum time interval between the adjacent points in the trajectory. We used Diper exclusively to calculate MSD with overlapping time windows[3] because it provides with a robust averaging method since the number of displacement vectors decreases with increasing time interval. The MSD from each track was then exported to Origin (Pro) and the data was fitted to Furth's formula according to persistent random walk (PRW) model of migration to obtain persistence time (P) and diffusion coefficient (D) according to:

$$MSD = 4D[\Delta t - P\left(1 - \exp\left(\frac{-\Delta t}{P}\right)\right)]$$

Fittings were performed in Origin (Pro) using the non-linear curve function Levenberg Marquardt. Only fits with R (square) closer to 1 was accepted. Examples of a good fit and a poor fit is shown (Supplementary Figure 1A and 1B). For migration under confinement, the MSD is visually very similar to what is expected from Persistent Random Walk where cells move persistently at short time intervals (<MSD> ~t^2) and more like random walk at longer time intervals (<MSD> ~t)[4]. Persistence and diffusion coefficient values were then plotted using GraphPad Prism.

2) Mathematical model formulation

A. Initial formulation of the model equations from general conservation principles

For the membrane protein of interest, with local density $P(x)$, its transport is characterized by local flux in the x direction, $N_p(x)$, defined according Fick's law of diffusion plus the potential contribution of advective flow:

$$N_p(x) = -D_p \frac{dP}{dx} + V_p P.$$

In this equation, $D_p(x)$ is the local protein diffusivity, and $V_p(x)$ is the local advection velocity of the protein; for the sake of generality, both are allowed to vary with position x at this point. Considering the geometry of the bleb, defined by length, L, and width, $w(x)$, the steady-state material balance for the membrane protein is

$$0 = -\frac{d}{dx}\left(N_p w\right) + R_p w,$$

where $R_p(x)$ is generally defined as the local rate of protein generation (net insertion into the membrane less the loss from the membrane, regardless of the mechanism(s) involved). A similar steady-state balance is invoked for the bulk membrane (mass density M), except for the consideration that there is, by definition, no diffusion of total mass:

$$0 = -\frac{d}{dx}(V_m M w) + R_m w.$$

In this equation, $V_m(x)$ is the local bulk membrane velocity, and $R_m(x)$ is the local rate of bulk membrane generation (net addition minus loss). Based on this formulation, the quantities $-R_p/P$ and $-R_m/M$ are defined as the local turnover frequencies of the membrane protein and of the bulk membrane, respectively.

B. Derivation of conditions for maintenance of any protein gradient

Starting from the general formulation posed above, we stipulate (supported by experimental observations, that the mass density of the bulk membrane is uniform (M is constant). Hence, we manipulate the balance on bulk membrane to obtain,

$$0 = -\frac{d}{dx}(V_m w) + \frac{R_m}{M} w,$$

and again by simply multiplying both sides by $P(x)$:

$$0 = \left(-\frac{d}{dx}(V_m w) + \frac{R_m}{M} w\right) P.$$

Turning to the protein balance, we incorporate the flux expression to obtain,

$$0 = -\frac{d}{dx}(N_p w) + R_p w = \frac{d}{dx}\left(D_p w \frac{dP}{dx}\right) - \frac{d}{dx}(V_p w P) + R_p w.$$

Manipulating the above, to separate the terms that contain the protein gradient, $\frac{dP}{dx}$,

$$\frac{d}{dx}\left(D_p w \frac{dP}{dx}\right) - V_p w \frac{dP}{dx} = P \frac{d}{dx}(V_p w) - R_p w.$$

Next, we add the bulk membrane balance (which is equal to zero on both sides) to the above:

$$\frac{d}{dx}\left(D_p w \frac{dP}{dx}\right) - V_p w \frac{dP}{dx} = P \frac{d}{dx}(V_p w) - R_p w + \left(-\frac{d}{dx}(V_m w) + \frac{R_m}{M} w\right) P.$$

Combining the terms on the right-hand side, we obtain,

$$\frac{d}{dx}\left(D_p w \frac{dP}{dx}\right) - V_p w \frac{dP}{dx} = \left\{\frac{d}{dx}\left[(V_p - V_m) w\right] - \left(\frac{R_p}{P} - \frac{R_m}{M}\right) w\right\} P.$$

Inspection of the above indicates that, if there is no gradient ($\frac{dP}{dx} = 0$), the right-hand side of the equation must be equal to zero. For this to be true throughout the domain, the only plausible scenario is

$$V_p(x) = V_m(x); \text{ and, } \frac{R_p(x)}{P} = \frac{R_m(x)}{M}.$$

This scenario may be stated thus: the membrane protein both flows with, and is turned over at the same frequency as, the bulk membrane. If this were true, then the proof of zero gradient throughout can be formalized; transforming the resulting equation into a first-order differential equation yields,

$$\frac{dY}{dx} - \frac{V_p}{D_p} Y = 0; \quad Y = D_p w \frac{dP}{dx}.$$

Given the boundary conditions,

$$N_p(L) = 0; \quad V_p(L) = V_m(L) = 0,$$

we conclude that $Y(L) = w(L)\left[-N_p(L) + V_p(L)P(L)\right] = 0$, and therefore the trivial solution is $Y(x) = 0$.

For the scenarios considered in this paper, we invoked the basic assumption,

$$\frac{R_p(x)}{P(x)} = -k_p = \frac{R_m(x)}{M} = -k_m.$$

In words, membrane protein (EGFR) is removed from the bleb at the same (constant) frequency as (along with) bulk membrane. A difference between the advective flow velocities of EGFR and bulk membrane ($V_p(x) \neq V_m(x)$) is sufficient to predict a gradient of EGFR in the bleb.

References

1. Meijering, E., Dzyubachyk, O. & Smal, I. Methods for Cell and Particle Tracking. in *Methods in Enzymology* vol. 504 183–200 (Elsevier, 2012).
2. Gorelik, R. & Gautreau, A. Quantitative and unbiased analysis of directional persistence in cell migration. *Nat Protoc* **9**, 1931–1943 (2014).
3. Dickinson, R. B. & Tranquillo, R. T. Optimal estimation of cell movement indices from the statistical analysis of cell tracking data. *AIChE Journal* **39**, 1995–2010 (1993).
4. Liu, Y.-J. *et al.* Confinement and Low Adhesion Induce Fast Amoeboid Migration of Slow Mesenchymal Cells. *Cell* **160**, 659–672 (2015).

Supplementary figure 1

A) Good fit example

Model	Furtz
Equation	4 * D * (x - P * (1 - exp(-x / P)))
Plot	MSD
Diffusion coefficient	141.43458 ± 10.21155
Persistence	15.13612 ± 1.65215
Reduced Chi-Sqr	7503.06311
R-Square (COD)	0.99823
Adj. R-Square	0.99815

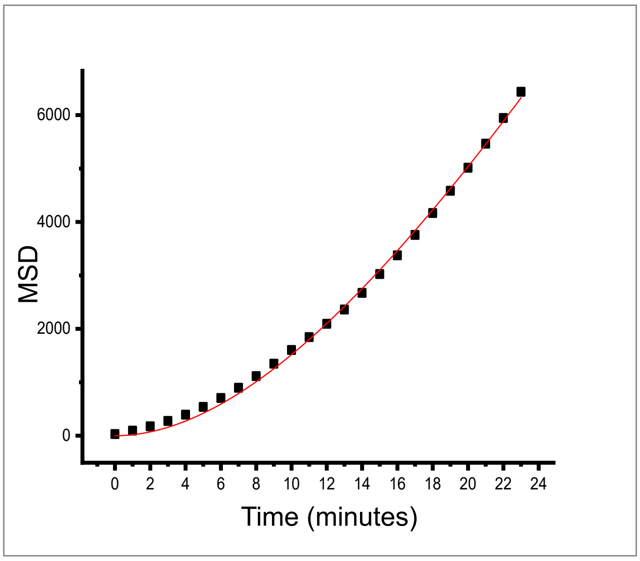

B) Poor fit example

Model	Furtz
Equation	4 * D * (x - P * (1 - exp(-x / P)))
Plot	MSD
Diffusion coefficient	1.00767 ± 3.66229
Persistence	2.88163E−8 ± 49.83256
Reduced Chi-Sqr	217174.83362
R-Square (COD)	−3.95717
Adj. R-Square	−4.1825

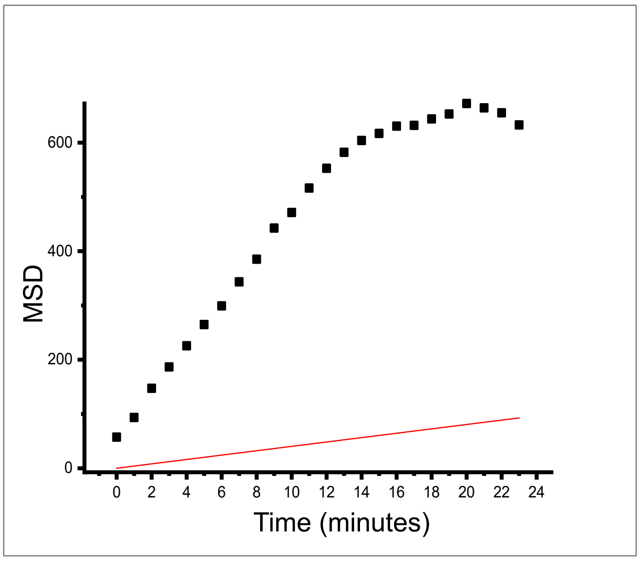

Supplementary figure 1. Mean square displacement (MSD) analysis of bleb-based cell migration under low confinement. Mean square displacement (MSD) as a function of time lag for cells migrating under low-adhesion confined environment. Square symbols represent experimentally measured MSD values, and solid red lines indicate fits to the equation (2) where (D) is the diffusion coefficient and (P) is the persistence time. Calculated diffusion coefficient and persistence time are calculated and mentioned in the table on the left. R-square (marked with orange box) shows the goodness of the fit. Right side has graphs **(A)** Example of a dataset and graph showing a good fit between the experimental MSD and the model, yielding a well-defined persistence time (P). **(B)** Example of a dataset and graph showing a poor fit, where deviations between the experimental MSD and the model indicate non-persistent or heterogeneous migratory behavior, resulting in an unreliable persistence estimate.
