# Supplementary material for: CD44 restricts EGFR mobility to polarize cytoskeletal signalling modules driving bleb-based migration
Source: Nat Cell Biol. 2026 Jul 6;28(7):1408–23. doi: 10.1038/s41556-026-01981-1 (PMC13364714; doi:10.1038/s41556-026-01981-1)
Supplement: Supplementary file 2 — Reporting Summary [file 41556_2026_1981_MOESM2_ESM.pdf]

nature portfolio

Corresponding author(s): Ankita Jha; Clare M Waterman

Last updated by author(s): 04/26/2026

Reporting Summary

Nature Portfolio wishes to improve the reproducibility of the work that we publish. This form provides structure for consistency and transparency in reporting. For further information on Nature Portfolio policies, see our Editorial Policies and the Editorial Policy Checklist.

Statistics

For all statistical analyses, confirm that the following items are present in the figure legend, table legend, main text, or Methods section.

n/a	Confirmed	
☐	☒	The exact sample size (*n*) for each experimental group/condition, given as a discrete number and unit of measurement
☐	☒	A statement on whether measurements were taken from distinct samples or whether the same sample was measured repeatedly
☐	☒	The statistical test(s) used AND whether they are one- or two-sided<br>*Only common tests should be described solely by name; describe more complex techniques in the Methods section.*
☐	☒	A description of all covariates tested
☐	☒	A description of any assumptions or corrections, such as tests of normality and adjustment for multiple comparisons
☐	☒	A full description of the statistical parameters including central tendency (e.g. means) or other basic estimates (e.g. regression coefficient) AND variation (e.g. standard deviation) or associated estimates of uncertainty (e.g. confidence intervals)
☐	☒	For null hypothesis testing, the test statistic (e.g. F, t, r) with confidence intervals, effect sizes, degrees of freedom and P value noted<br>*Give P values as exact values whenever suitable.*
☒	☐	For Bayesian analysis, information on the choice of priors and Markov chain Monte Carlo settings
☒	☐	For hierarchical and complex designs, identification of the appropriate level for tests and full reporting of outcomes
☐	☒	Estimates of effect sizes (e.g. Cohen's *d*, Pearson's *r*), indicating how they were calculated

Our web collection on statistics for biologists contains articles on many of the points above.

Software and code

Policy information about availability of computer code

Data collection	Data was collected on Nikon Elements, Zeiss Zen, 4Dcell Confinement Package
Data analysis	Analysis was performed on Imaris and ImageJ 1.54, Graphpad Prism 9 and 10, Origin Lab, MATLAB. Microsoft Excel

For manuscripts utilizing custom algorithms or software that are central to the research but not yet described in published literature, software must be made available to editors and reviewers. We strongly encourage code deposition in a community repository (e.g. GitHub). See the Nature Portfolio guidelines for submitting code & software for further information.

Data

Policy information about availability of data

All manuscripts must include a data availability statement. This statement should provide the following information, where applicable:

- Accession codes, unique identifiers, or web links for publicly available datasets
- A description of any restrictions on data availability
- For clinical datasets or third party data, please ensure that the statement adheres to our policy

All data needed to evaluate the conclusions are provided in the main text and figures, extended data figures or supplementary text. All the unprocessed immunoblots or raw data and associated statistical calculations are provided with this study. Source data are provided with this paper with data points and statistical analysis.

Research involving human participants, their data, or biological material

Policy information about studies with human participants or human data. See also policy information about sex, gender (identity/presentation), and sexual orientation and race, ethnicity and racism.

Reporting on sex and gender	N/A
Reporting on race, ethnicity, or other socially relevant groupings	N/A
Population characteristics	N/A
Recruitment	N/A
Ethics oversight	N/A

Note that full information on the approval of the study protocol must also be provided in the manuscript.

Field-specific reporting

Please select the one below that is the best fit for your research. If you are not sure, read the appropriate sections before making your selection.

☒ Life sciences ☐ Behavioural & social sciences ☐ Ecological, evolutionary & environmental sciences

For a reference copy of the document with all sections, see nature.com/documents/nr-reporting-summary-flat.pdf

Life sciences study design

All studies must disclose on these points even when the disclosure is negative.

Sample size	No statistical methods were used to pre-determine sample sizes but our sample sizes are in line with the field (references)
Data exclusions	No data points were excluded from the analysis.
Replication	All experiments include multiple biological and sample replicates as described in either the figure legends or methods sections and in source data. Reproducibility was determined based on at least three individual repeats.
Randomization	Acquisition of images of samples were performed in random.
Blinding	Blinding was not performed for data collection or analysis as the experimental conditions were analyzed using unbiased methods.

Reporting for specific materials, systems and methods

We require information from authors about some types of materials, experimental systems and methods used in many studies. Here, indicate whether each material, system or method listed is relevant to your study. If you are not sure if a list item applies to your research, read the appropriate section before selecting a response.

Materials & experimental systems

n/a	Involved in the study	
☐	☒	Antibodies
☐	☒	Eukaryotic cell lines
☒	☐	Palaeontology and archaeology
☒	☐	Animals and other organisms
☒	☐	Clinical data
☒	☐	Dual use research of concern
☒	☐	Plants

Methods

n/a	Involved in the study	
☒	☐	ChIP-seq
☒	☐	Flow cytometry
☒	☐	MRI-based neuroimaging

Antibodies

Antibodies used	CD44 (BJ18; BioLegend), Ezrin (3C12; Invitrogen), EGFR (H11; Invitrogen), or phospho-ERM (Thr567/564/558; Cell Signaling Technology), secondary antibodies (1:500; Jackson ImmunoResearch) and Alexa Fluor 647–phalloidin (1:200; Thermo Fisher Scientific), anti-phospho-EGFR (1:1000; Tyr1068, D7A5 Cell signaling technology), anti-EGFR (1:1000; D381B1, Cell signaling

	technology), anti-CD44 (BJ18, Biolegend), anti-phospho-Akt (1:2000; Ser473, D9E, Cell signaling technology), anti-Ezrin (3145, Cell signaling technology), anti-GAPDH (14C10, Cell signaling technology). The secondary antibodies (HRP-conjugated goat anti-mouse (115-035-003) or goat anti-rabbit (111-035-003)
Validation	All antibodies were commercially validated by the relevant manufacturer and information is readily available from their websites. Additionally Atlas antibodies was consulted for reproducibility.

Eukaryotic cell lines

Policy information about cell lines and Sex and Gender in Research

Cell line source(s)	A375MA2 cells were obtained from American Type Culture Collection (ATCC CRL-3223, ATCC, Manassas, VA) , HEK293FT (ATCC, CRL-3216)
Authentication	All cell lines were purchased from a commercial vendor. No other methods of authentication was utilized.
Mycoplasma contamination	All cell lines were routinely inspected for contamination and confirmed negative.
Commonly misidentified lines (See ICLAC register)	No commonly misidentified lines per ICLAC register were used

Plants

Seed stocks	N/A
Novel plant genotypes	N/A
Authentication	N/A
