# Supplementary material for: CD44 restricts EGFR mobility to polarize cytoskeletal signalling modules driving bleb-based migration
Source: Nat Cell Biol. 2026 Jul 6;28(7):1408–23. doi: 10.1038/s41556-026-01981-1 (PMC13364714; doi:10.1038/s41556-026-01981-1)
Supplement: Supplementary file 19 — Unprocessed western blots. [file 41556_2026_1981_MOESM19_ESM.pdf]

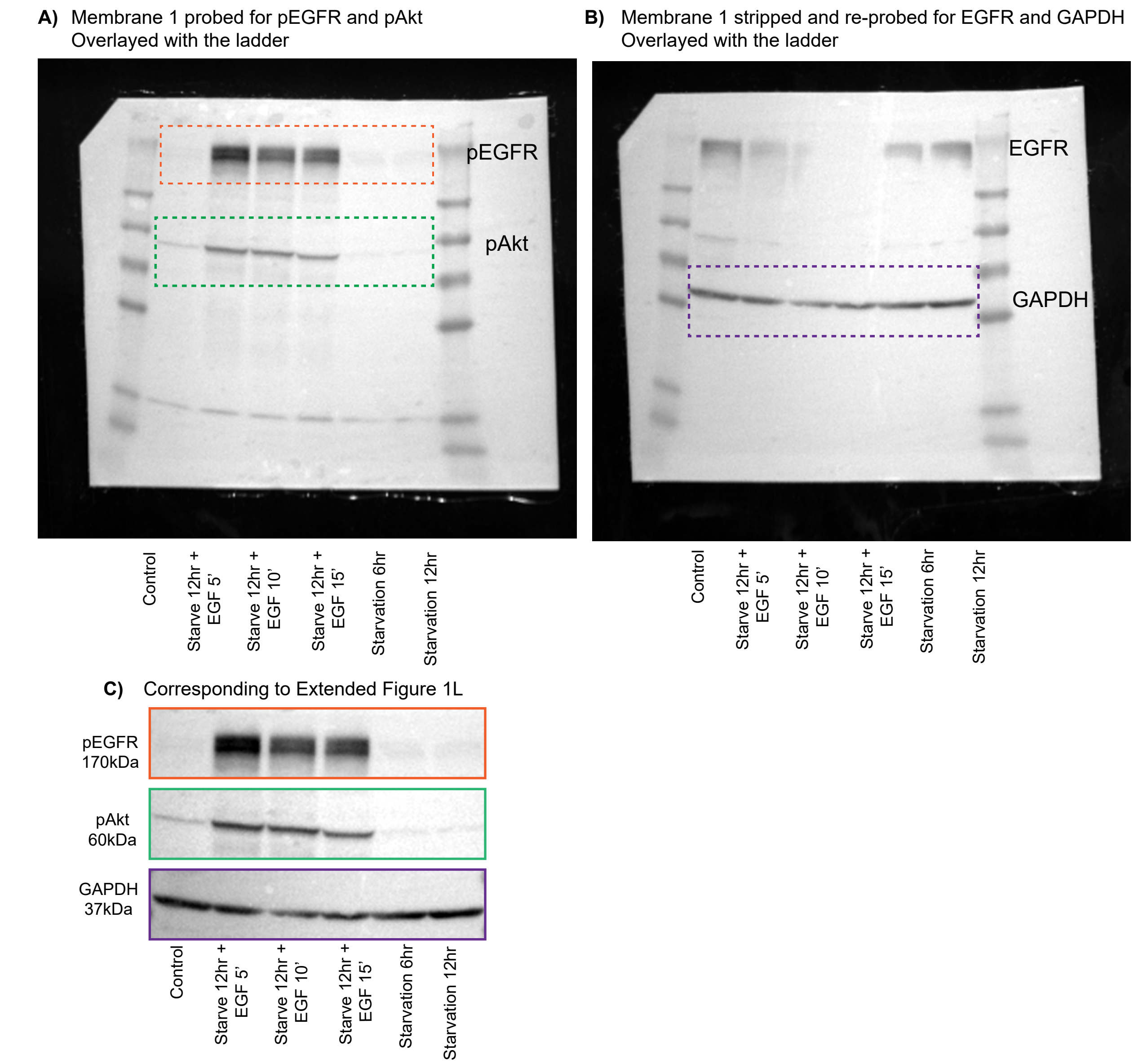

Uncropped immunoblots shown in Extended Figure 1L. Colored outlines identify the main regions in the Extended Figure 1L, color coded to match the sections from the figure. Membrane were first probed for pEGFR and pAkt (A) and stripped prior to re-probing for EGFR and GAPDH. GAPDH is used for loading controls.

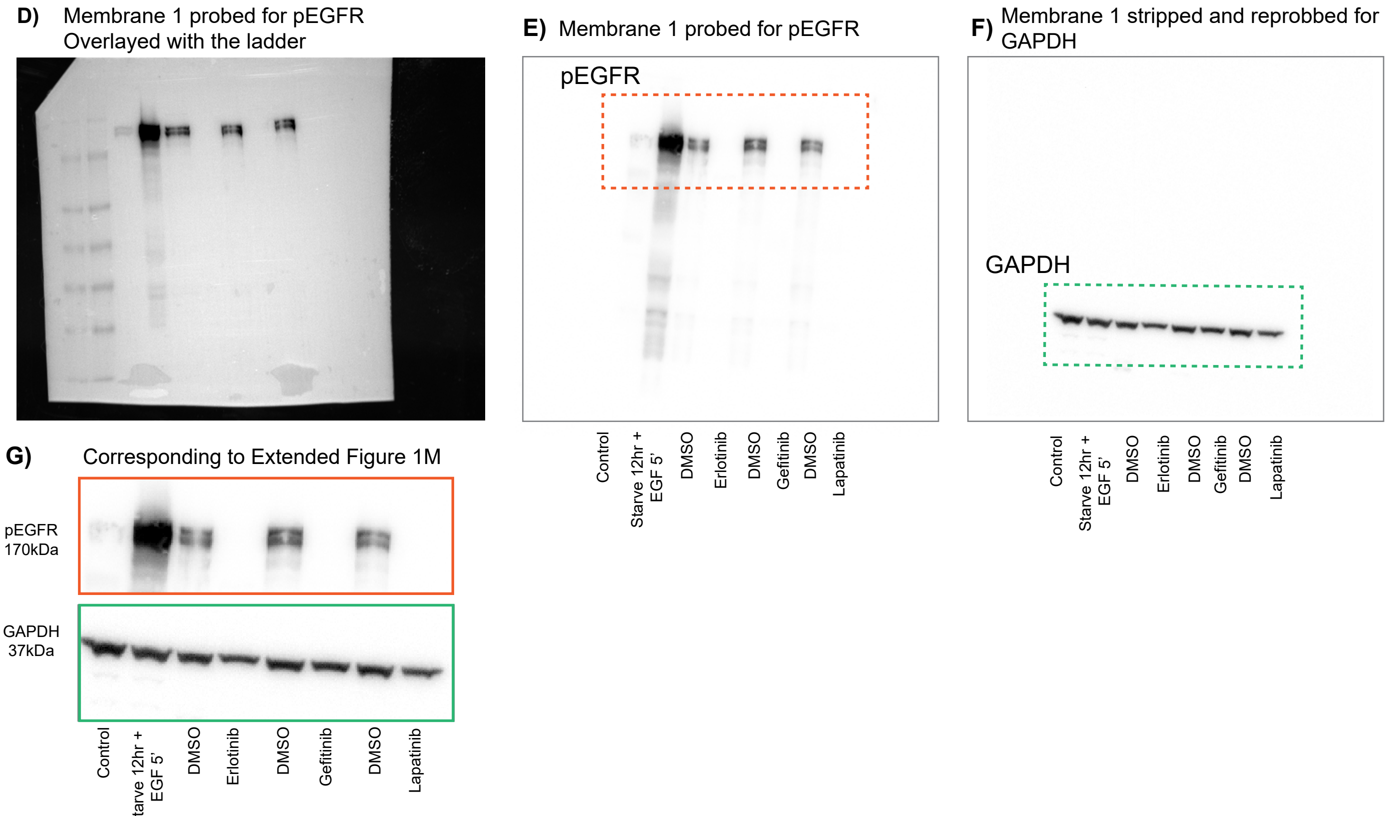

Uncropped immunoblots shown in Extended Figure 1M. Colored outlines identify the main regions in the Extended Figure 1M, color coded to match the sections from the figure. Membrane were first probed for pEGFR (E) and stripped prior to re-probing for GAPDH (F). GAPDH is used for loading controls.

H) Membrane 1 probed for EGFR and GAPDH
Overlayed with the ladder

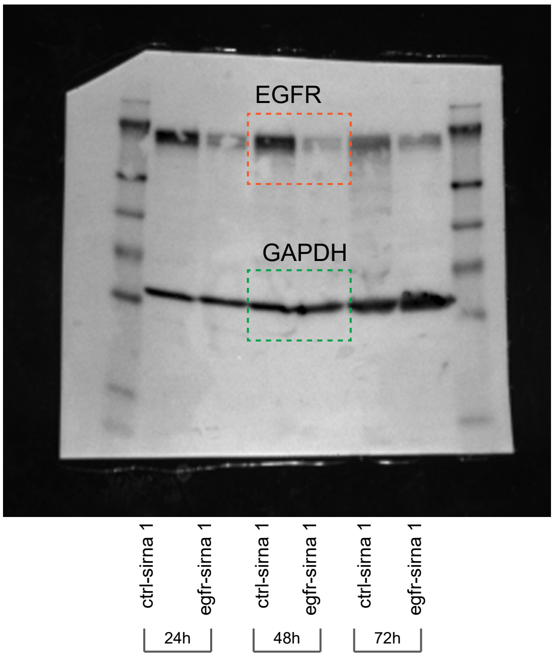

I) Corresponding to
Extended Figure 1N (left panel)

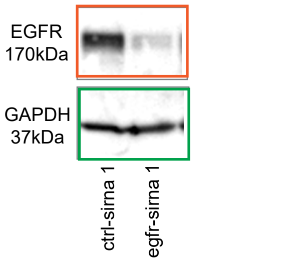

J) Membrane 1 probed for EGFR
Overlayed with the ladder

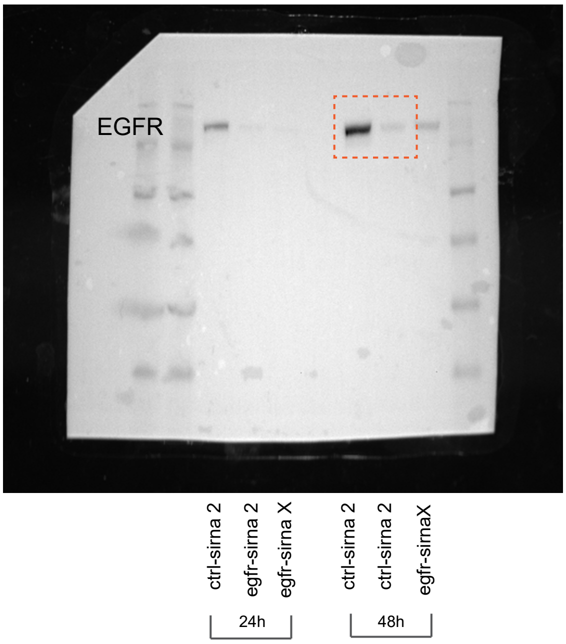

K) Membrane 1 stripped and re-probed with GAPDH
Overlayed with the ladder

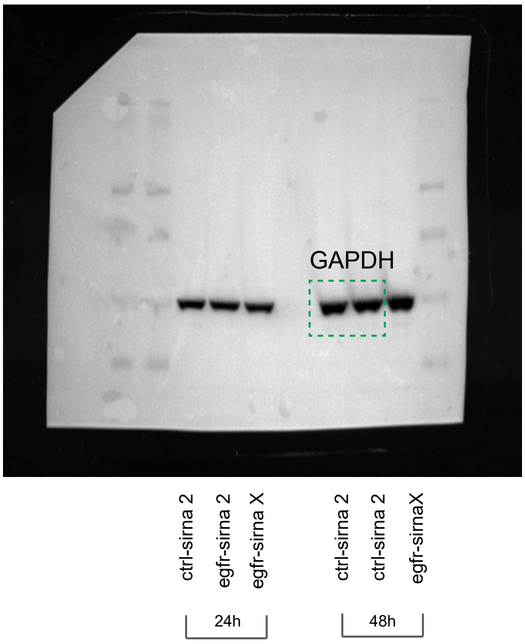

L) Corresponding to
Extended Figure 1N

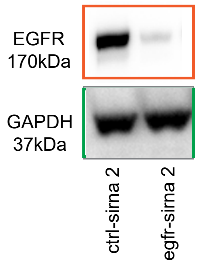

Uncropped immunoblots shown in Extended Figure 1N (both left and right panel). Colored outlines identify the main regions in the Extended Figure 1N, color coded to match the sections from the figure corresponsing to the different sirna sequences that are used in this manuscript. Membrane were probed for EGFR and GAPDH(H). For another experiment membrane was probed with EGFR first (J) and then stripped and reblotted for GAPDH (K). GAPDH is used for loading controls.

M) Membrane 1 probed for Ezrin
Overlayed with the ladder

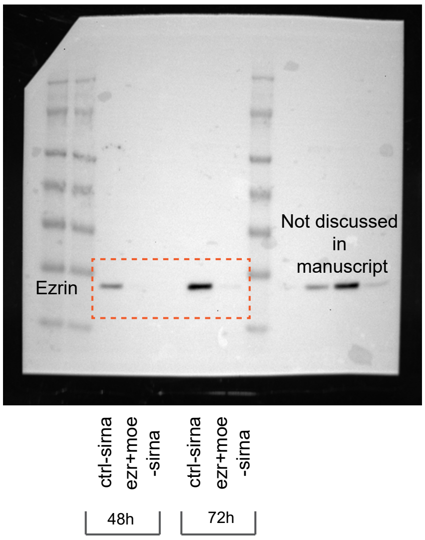

N) Membrane 1 stripped and re-probed with Gapdh
Overlayed with the ladder

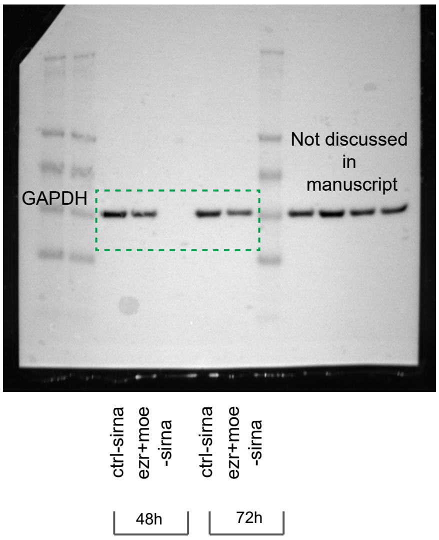

O) Corresponding to
Extended Figure 4C

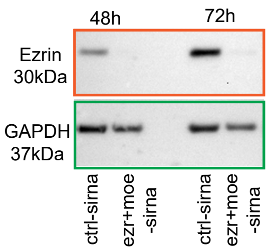

Uncropped immunoblots shown in Extended Figure 4C (O). Colored outlines identify the main regions in the Extended Figure 4C, color coded to match the sections from the figure. Membrane were first probed for Ezrin (M) and stripped prior to re-probing for GAPDH (N). GAPDH is used for loading controls.

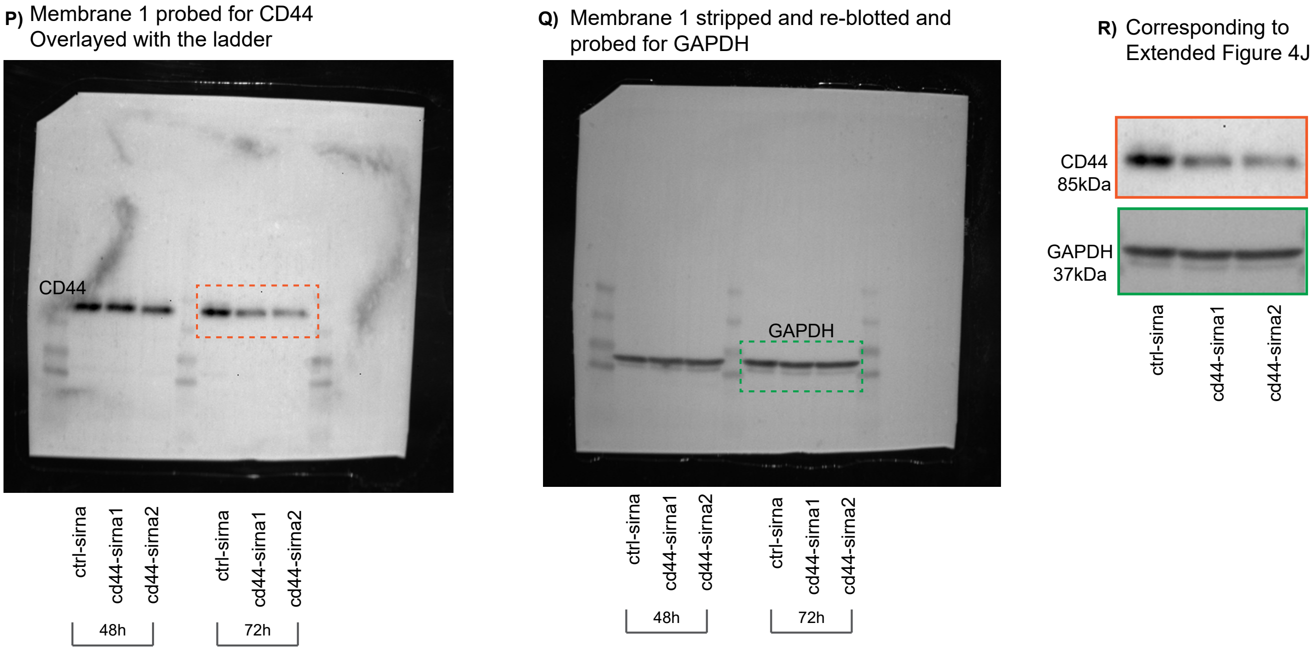

Uncropped immunoblots shown in Extended Figure 4J (R). Colored outlines identify the main regions in the Extended Figure 4J, color coded to match the sections from the figure. Membrane were first probed for CD44 (P) and stripped prior to re-probing for GAPDH (Q). GAPDH is used for loading controls.
