# Supplementary material for: CD44 restricts EGFR mobility to polarize cytoskeletal signalling modules driving bleb-based migration
Source: Nat Cell Biol. 2026 Jul 6;28(7):1408–23. doi: 10.1038/s41556-026-01981-1 (PMC13364714; doi:10.1038/s41556-026-01981-1)
Supplement: Supplementary file 3 — Peer Review File [file 41556_2026_1981_MOESM3_ESM.pdf]

nature portfolio

Peer Review File

CD44 restricts EGFR mobility to polarize cytoskeletal signaling modules driving bleb-based migration.

Corresponding Author: Dr Ankita Jha

Version 0:

Decision Letter:

*Please delete the link to your author homepage if you wish to forward this email to co-authors.

Dear Dr Jha,

I apologize once again for the delay. As mentioned, there was an initial delay in securing sufficient reviewer expertise to comment on your manuscript, then the reviewers were overdue in their reports. We have now received all the reports.

Your manuscript, "CD44 and Ezrin restrict EGF receptor mobility to generate a novel spatial arrangement of cytoskeletal signaling modules driving bleb-based migration", has now been seen by 3 referees, who are experts in actin cortex – membrane interaction (referee 1); cell migration and the cytoskeleton (referee 2); and RTK signalling (referee 3). As you will see from their comments (attached below) they find this work of potential interest, but have raised substantial concerns, which in our view would need to be addressed with considerable revisions before we can consider publication in Nature Cell Biology.

Nature Cell Biology editors discuss the referee reports in detail within the editorial team, including the chief editor, to identify key referee points that should be addressed with priority, and requests that are overruled as being beyond the scope of the current study. To guide the scope of the revisions, I have listed these points below. We are committed to providing a fair and constructive peer-review process, so please feel free to contact me if you would like to discuss any of the referee comments further.

In particular, it would be essential to:

A) Assess potential roles of EGFR activity vs. localization and dynamics in your system with further experimental data (all Reviewers).

B) Further test, and disambiguate if possible, the roles of polarity vs. bleb formation vs. migration speed (all Reviewers).

C) Discuss potential physiological relevance given conditions that migrating leader cells may encounter with or without ECM or other cells (Reviewer #2).

D) All other referee concerns pertaining to strengthening existing data, providing controls, methodological details, clarifications and textual changes, should also be addressed.

E) Finally please pay close attention to our guidelines on statistical and methodological reporting (listed below) as failure to do so may delay the reconsideration of the revised manuscript. In particular please provide:

- a Supplementary Figure including unprocessed images of all gels/blots in the form of a multi-page pdf file. Please ensure that blots/gels are labeled and the sections presented in the figures are clearly indicated.

- a Supplementary Table including all numerical source data in Excel format, with data for different figures provided as different sheets within a single Excel file. The file should include source data giving rise to graphical representations and statistical descriptions in the paper and for all instances where the figures present representative experiments of multiple independent repeats, the source data of all repeats should be provided.

We would be happy to consider a revised manuscript that would satisfactorily address these points, unless a similar paper is published elsewhere, or is accepted for publication in Nature Cell Biology in the meantime.

When revising the manuscript please:

- ensure that it conforms to our format instructions and publication policies (see below and www.nature.com/nature/authors/).

- provide a point-by-point rebuttal to the full referee reports verbatim, as provided at the end of this letter.

- provide the completed Editorial Policy Checklist (found here https://www.nature.com/authors/policies/Policy.pdf), and Reporting Summary (found here https://www.nature.com/authors/policies/ReportingSummary.pdf).

This is essential for reconsideration of the manuscript and these documents will be available to editors and referees in the event of peer review. For more information see http://www.nature.com/authors/policies/availability.html or contact me.

Nature Cell Biology is committed to improving transparency in authorship. As part of our efforts in this direction, we are now requesting that all authors identified as 'corresponding author' on published papers create and link their Open Researcher and Contributor Identifier (ORCID) with their account on the Manuscript Tracking System (MTS), prior to acceptance. ORCID helps the scientific community achieve unambiguous attribution of all scholarly contributions. You can create and link your ORCID from the home page of the MTS by clicking on 'Modify my Springer Nature account'. For more information please visit please visit www.springernature.com/orcid.

Please submit the revised manuscript files and the point-by-point rebuttal to the referee comments using this link:

Link Redacted

*This url links to your confidential home page and associated information about manuscripts you may have submitted or be reviewing for us. If you wish to forward this email to co-authors, please delete the link to your homepage.

We would like to receive a revised submission within six months. We would be happy to consider a revision even after this timeframe, however if the resubmission deadline is missed and the paper is eventually published, the submission date will be the date when the revised manuscript was received.

We hope that you will find our referees' comments, and editorial guidance helpful. Please do not hesitate to contact me if there is anything you would like to discuss.

Best wishes,

Daryl

Daryl Jason Verzosa David, PhD

Senior Editor, Nature Cell Biology
Advisory Editor, npj Biological Physics and Mechanics
Nature Portfolio

Heidelberger Platz 3, 14197 Berlin, Germany
Email: daryl.david@nature.com
ORCID: https://orcid.org/0000-0002-9253-4805

Reviewers' Comments:

Reviewer #1 (Remarks to the Author):

Metastatic tumor cells adopt bleb-driven mode of protrusion when ECM ligands are unavailable or integrin signaling is suppressed. However, the precise molecular mechanisms underlying bleb-based migration remain unclear; in particular, the role of polarized receptor signaling in symmetry breaking and contractility gradients across blebs remains unknown. In this paper, Jha and colleagues have combined a series of molecular sensors, high-resolution imaging, and genetic manipulations to propose a novel molecular mechanism for establishing cell polarity in bleb-driven migration. The authors focused on the formation and maintenance of elongated leader blebs, which were shown to be correlated with efficient and persistent migration. Notably, perturbation of EGFR signaling through inhibition or knockdowns results in a decrease of leader bleb population and consequent reduction of migratory range. By using a series of molecular sensors, the authors unveiled a rear-to-front gradient of EGFR localization and activity in leader which is transmitted to downstream Rac1 signaling. In contrast, RhoA is polarized at the bleb tip; thus, bleb-based migration exhibits a reverse Rac1/RhoA polarity compared to mesenchymal cell migration. In accordance with the previous findings, disruption of EGFR/Rac1 polarity through optogenetic manipulation results in reduction of leader bleb length, actin disassembly and bleb retraction. FRAP experiments combined with mathematical modelling revealed that the EGFR gradient originates from a "corralling" of EGFR at the bleb rear, where it resists anterograde membrane flow. This "corralling" appears to be driven by accumulation of CD44, ezrin/moesin, and membrane-proximal actin at the rear of the bleb, resulting in a decrease of EGFR mobility. Further depletion of CD44 or ezrin/moesin abolishes rear-to-front EGFR gradients hence decreasing polarity. In the case of CD44 depletion, the Rac1 gradient is also flattened, ultimately inhibiting leader bleb formation and reducing migratory range.
In summary, the authors have unveiled a novel molecular mechanism for establishing rear-to-front EGFR and Rac1 polarity in bleb-driven migration, opposite to what is observed in mesenchymal cell migration. This polarity appears to be driven by a membrane "corralling" mechanism where CD44, ezrin-moesin and membrane-proximal actin act synergistically to restrict EGFR mobility. Loss of this gradient through various perturbations ultimately affects leader bleb stability and migration persistence. Overall, I consider that the conclusions of this study are well substantiated, and the proposed molecular mechanism is thoroughly explained and fits with the experimental data. I personally find that as its currently written this manuscript does not convey what I think the most significant finding of this work, which is the unveiling of a reverse signaling polarity network in bleb-driven migration; in light also of my first minor point below I would change the order of figures but leave that for the authors to decide.
Either way, I believe that only a few points need to be addressed to bring this study to completion:
• Figure 1 directs the reader to a possible role of EGFR in bleb-driven migration, for which the data is not fully conclusive. The role of EGFR is based on its inhibition by drugs (which are not totally specific) while the siRNA appears to have quite an effect even in the control case, since the control iRNA from Figure 1K has a much lower persistence than the DMSO controls (Figure 1H), suggesting a possible toxicity per se. In fact, the persistence appears negative, is there an error in that plot? If not, could the authors clarify how these

values can be achieved? In any case, as the authors acknowledge, the difference between control and EGFR-KD is not significant, which does not fully support a key role of EGFR in leader bleb formation and persistent migration. I am not questioning the sufficiency of EGFR (shown in figure 3D-E) but the necessity. Is this something that lead-bleb migrating cells need? I would encourage the authors to make a cleaner KD or ADS or KO.
• In Figure 4, the authors explain how "Experiment and modeling indicate neither cortical nor membrane flow mediate GFR gradient formation in leader blebs". While the experimental data clearly supports the claim, the relevance of the mathematical model for the results is unclear. Could the authors provide further clarification on the model and its applications in the discussion section?
• In Figure 5, the authors demonstrate that cells migrating on 3D fibrillar ECM, CD44 and EGFR also accumulate at the rear. To understand whether the rear-to-front polarity is fully recapitulated in 3D environments, Rac1 and RhoA sensors could be imaged in the same conditions, as well as ezrin and moesin. To facilitate the comprehension of this point, I would propose to include the EGFR imaging together with the suggested Rac1 and RhoA sensor imaging in Figure 2, while the CD44 and suggested ezrin/moesin images could remain in Figure 5. The authors could further strengthen the robustness of this phenotype by adding quantifications of gradients in 3D.
• In Figure 5D, the rear-to-front ezrin and CD44 gradients appear to be of different magnitudes according to the quantification plot, with CD44 exhibiting a much higher intensity at the rear of the bleb compared to ezrin. However, this is not clearly displayed in the representative image, where they appear to be similar. Could the authors 1) provide representative images which better reflect the quantifications and 2) address in the discussion why the gradients would have different degrees of intensity?
• The loss of EGFR/Rac1 gradients through direct genetic perturbations or CD44 depletion has a more striking effect in migration persistence compared to speed. Indeed, migration speed is not affected upon EGFR pharmacological inhibition as well as upon depletion of CD44. While I acknowledge that addressing this discrepancy would likely require a series of experiments outside the scope of this paper, the authors could propose their interpretation about these observations in the discussion.

Additional minor points:
• For some of the representative microscopy images, the authors could overlay lines to delimit the cell body and the bleb and make the figure clearer to the readers. For instance, in Figures 5D and 5G this would facilitate the identification of the ezrin and CD44 gradients between the bleb rear and tip.
• The same group already suggested the involvement of ezrin in the stability of leader blebs a decade ago. In the discussion they wrote: "Our demonstration the Eps8 localizes to bleb membranes on a similar timescale as ezrin suggests that their interaction (Zwaenepoel et al., 2012) may be critical to Eps8 cortical recruitment". It would be important to refer to their work when ezrin is first introduced in the manuscript.
• I would recommend the authors to also refer to Driscoll et al 2024 Dev Cell, as they nicely showed the importance of PI3K signalling for bleb formation and size.

Reviewer #2 (Remarks to the Author):

In this manuscript Jha and colleagues set out to investigate the molecular mechanisms that underlie the polarization of cytoskeletal signaling proteins and membrane receptors in leader bleb migration. Leader blebs are large pressure driven protrusions that many cells form in response to confinement and are thought to be an integrin independent mode of migration that cancer and other cells can utilize to navigate through low-adhesive confined environments. Previous work from this lab established that Erk signaling plays a prominent role in facilitating leader bleb migration and thus this current works set out to determine whether downstream signaling components displayed specific polarized distributions along the leader bleb, and whether these distributions were crucial for leader-bleb migration. They find that, surprisingly, the traditional (i.e. in mesenchymal migration) polarization of Rac and Rho are flipped in leader blebs, with Rac localizing at the base of the bleb and Rho localizing to the leading edge. Using biosensors and fluorescent constructs they then show that this polarization is established through the restricted mobility of EGFR by CD44 and ERM proteins, which concentrates Rac locally at the base of the bleb.

Overall this is a compelling series of observations that shows that EGFR/CD44/ERM/RAC/PIP3 are all spatially polarized with increased concentrations at the base of the leader bleb, and that PIP2/RhoA are concentrated at the leading edge of the bleb. Activation of EGFR, or its downstream effector Rac, at the bleb leading edge results in retraction of the leader bleb. Similarly, knockdown of CD44 or ERM proteins results in a loss of this gradient and results in reduced leader bleb formation. This seems like a reasonable model and the authors have done a good job breaking it down by components. There are a few comments/questions, however, about the data/interpretation:

One of the most surprising results of all these perturbations that disrupt this polarity, is that they have minimal effect on the persistence (Fig. 1K, 7E) or migration speed (EF. 1J, 5B) of leader bleb cells. How does one reconcile this with the argument that this polarity is crucial for leader bleb migration? Is it just the number of leader bleb cells that is impacted?

With the migration analysis, the speeds are plotted as MSD, while the persistence is found by fitting the Furth equation. From the methods it's unclear how the data is being fit, or what the quality of this fit is. Typically, when calculating the MSD with overlapping time windows, you can only trust a small percentage of the actual MSD. The citation provided ([84]) mentions that normally only the lower 10% of the low-end time intervals are used for determining the slope. Given the parameters here (1 min intervals for 60 min) that would be a deltaT of only 6 min. That reference also explicitly states "DiPer does not calculate persistence time and other parameters that stem from Furth's formula" and includes a healthy discussion of the limitations of defining persistence in this way. What part of the MSD data was used to fit the equation? How good were the fits (this is never shown anywhere)? And is the persistence from the Furth equation a good measurement for this type of migration? Finally, it's unclear from the plots whether the MSD is calculated for all the cells or just the cells with leader blebs.

The difference in kinetics of the optoEGFR and PA-Rac is striking. Why does the EGFR take 25 minutes to show a difference, while the Rac has an effect within just a few minutes? This is confusing too given that the FRAP experiments in Fig. 6A demonstrate that turnover is quite rapid throughout the bleb.

In the polarization measurements of EGFR, there is a clear peak about 25% of the way up the bleb (Fig. 2B, 3A, and seen most easily in

Movie 12 on the left). Why does the EGFR concentrate in this area, while Rac/PIP3/CD44 peak at the base of the bleb? Also, comparing the polarization plots of EGFR-GFP (Figs. 2C, 3C) the distributions don't seem to match very well. What's the difference in conditions between these two plots?

Many of the blebs in these movies seem quite stable and the cell isn't moving much. Is there a correlation between the magnitude of the gradient and the speed of the migration? Does this suggest that bleb polarization is in part decoupled from force transmission?

Since CD44 is helping to stabilize the leader blebs, does overexpression of CD44 result in more stable blebs? And do those cells move faster?

How does CD44 sequester EGFR when the EGFR seems to go further into the bleb during formation than the CD44 (Fig. 5C vs Fig. 6J)? What's causing the initial EGFR retraction towards the bleb base? They only really seem to overlap starting at the 8 min mark.

I understand the basic argument that the transmembrane interaction of CD44/EGFR is helping to activate PIP3/Rac and sequester it locally, but what is driving the localization of Rho to the tip of the bleb? Is it merely a response to local antagonism from Rac activation? Or is it also an actively maintained population?

Lastly, while this is a reproducible phenomenon in vitro where low-adhesion coupled with confinement produces these leader blebs, I struggle with where the cell might encounter such an environment physiologically. While certainly confinement is rampant in physiological systems, it is almost always in the presence of extracellular matrix proteins, or other cells where ligands are abundant (like in Fig. 5D). It would be useful to comment on this seeming contradiction.

Minor points:
- The modeling component was a bit unclear. Why are two different epsilon variables needed? This wasn't explained well in the main text and seemed mostly just to be fitting the experimental data (which was much more convincing).

- The data presentation isn't always consistent. Sometimes individual data points are shown on the violin plots (e.g. Fig. 2C) and not on others (Fig. 2G,I,J) even though there are no significant differences in the number of cells compared.

- In Fig 7E the boxplot have 3 points on them, but the legend is unclear what they represent. Are these the average values of the biological repeats? Do the boxplots represent the combined data? And were statistics performed on the combined individual data points, or the means of the biological repeats?

- The circle in the PA-Rac is given significantly different dimensions throughout the manuscript. In the text (line 303) is says 5 um diameter, Figure 3F says 20 um, movie 5 shows 10 um diameter

- Movie 8 has the actin channel repeat twice before switching to show a single loop of the EGFR?

- It's notoriously hard to make glass truly non-adhesive even at high concentrations of PLL-PEG (PMID: 38781207). Low-adhesive might be a better term.

Reviewer #3 (Remarks to the Author):

Jha et al

CD44 and Ezrin restrict EGF receptor mobility to generate a novel spatial arrangement of cytoskeletal signaling modules driving bleb-based migration

In this manuscript Jha et al set out to identify mechanisms mediating polarized bleb-based migration of metastatic melanoma cells in a confined, non-adhesive environment. Increasing evidence suggests that some tumors, especially melanoma, invoke bleb-based motility as opposed to the much better-studied mesenchymal mode of invasion, so the more we understand how it works the better. Using a sophisticated toolkit of super-resolution confocal imaging, biosensors, and optogenetic experiments together with a careful cell biological analysis of bleb formation in melanoma cells they identified a rear-to-front EGFR gradient that polarizes PI3K-Rac signaling through a mechanism involving the restriction of EGFR mobility at least in part, through CD44 and ezrin.

This well-written experimentally solid manuscript addresses an important cell biological aspect of tumor behavior. The authors missed some opportunities to embrace the complexity of this seemingly simple process and acknowledge necessary limitations of their approach. With some key additional experiments and discussion, it should make an important publication in Nature Cell Biology.

Major points:

Specificity: After a nice morphogenetic analysis of bleb formation in these cells, the authors jump to EGFR as a mechanism largely because they contrast their findings with studies of mesenchymal migration. While they present evidence that EGFR is polarized in leader blebs and contributes to their formation, the effects are somewhat modest, and the phenotype of erlotinib treatment appears to be different from wortmannin treatment. Moreover, the focus on EGFR masks the important question of whether the biology of bleb polarization is specifically driven by one or a subset of receptors and corralling mechanisms. The authors should test the involvement of another receptor (ie FGFR) and another potential ERM-picket (ie ICAM) for comparison to address this important question. It is interesting either way.

EGFR activity vs clustering: The evidence that EGFR activity is required as opposed to clustering/aggregation is modest. In Fig 3 the use

of the optical sensor is a nice tool but should be done with and without EGF and in the presence of erlotinib in order to draw the conclusion that EGFR signaling versus just clustering is important. Additionally, are the distributions of the EGFR, AKT and PAK sensors blocked by erlotinib?

MAPK signaling: These melanoma cells harbor an activating BRAF mutation and this group has previously argued that ERK is required for bleb-based migration (Logue et al. 2015). The authors should discuss their results in the context of these previous results. In the introduction it is pointed out that this signaling is linked to EGFR/Ras/Raf pathways, but it is not addressed further.

Requirement for the leader bleb creation versus polarization – can these be segregated? The optogenetic experiments work to make this distinction. For each perturbation (ie interfering with EGFR, CD44, ERMs), but the authors should show how the distribution of bleb phenotypes is altered (ie purple bar graphs) and relate it to the knockdown efficiency (which is not shown). It may be hard to fully segregate but this should be addressed up front and discussed.

How do you harmonize your observations with the established dynamic role of ERM proteins bound to PIP2 in bleb retraction? Indeed, Ezrin looks to be also enriched at the bleb tip in Fig 5D exactly as you would expect. The authors should at least discuss how the state of multiple small blebs, that are perhaps retracted by PIP2-bound ERM proteins, transition to a single elongated bleb with ERMs also concentrated at the base. Do ERM proteins also localize similarly in 5G?

Other Points:

- Please clarify the concentration of drugs used. The values given in the text, figure legend and methods are different. If erlotinib is being used at 10uM that is extremely high and lower doses should be used but I think this is a typo.

- Can you apply statistics to Fig 1G?

- Is 1J it statistically significant? the shRNA EGFR data is quite modest - do these cells express other ErbB family members?

- Ext. Fig. 1F: Include color scale on temporal projection

- Ext. Fig. 1K: This doesn't really tell you that EGFR signaling is normally maintaining pEGFR or pAkt in these cells, but rather that they can be stimulated and seen at short timepoints. To argue that EGF is the 'serum factor responsible for promoting leader bleb-based migration…' would require replacing serum with EGF and measuring steady-state levels after >24h.

- Ext. Fig. 1N: Was this blot carried out under steady state conditions or with EGF stimulation? Results are inconsistent with steady state signaling from the blot in K.

- add back a kinase dead version of EGFR to confirm that EGFR activity

- It would be helpful to include EGF concentration in the figure legend.

- There is a typo in line 341

- Fig. 3: Why is the timescale so different for EGFR and Rac optogenetic experiments? In the Rac optogenetic experiment a 4 min. timepoint is used – at this point is it possible to distinguish between a leader and dynamic bleb? The retraction results seem quite modest. It is difficult to see lamellipodia protrusions in 3F – it would be helpful to show a zoomed inset.

- Fig. 3: it is not clear that EGFR activity is required - this is exogenous
-
- Fig. 5G: is this endogenous EGFR? The addition of an additional model is nice, but it would be helpful to use it in additional contexts in the manuscript.

- Fig. 6: Evidence of CD44, ezrin, and moesin knockdown should be shown.

- When the authors say: 'Neither CD44 or ezrin/moesin knockdown impacted actin organization at the base of the bleb' what do they mean? the images in Ext. Fig. 4E and F look quite different

- Are the phenotypes described here specific to this cell line, or generalizable to other melanoma cell lines or other metastatic tumor cells?

Finally, the manuscript would benefit from additional discussion of the role of bleb-based migration in melanoma and melanoma metastasis, and how this work advances that field in general (ie Do melanomas largely use bleb-based amoeboid migration? What other tumors do?). Also a discussion of how this work meshes with earlier studies of the reverse polarity of these same melanoma cells (Lorentzen et al 2011).

GUIDELINES FOR MANUSCRIPT SUBMISSION TO NATURE CELL BIOLOGY

READABILITY OF MANUSCRIPTS – Nature Cell Biology is read by cell biologists from diverse backgrounds, many of whom are not

native English speakers. Authors should aim to communicate their findings clearly, explaining technical jargon that might be unfamiliar to non-specialists, and avoiding non-standard abbreviations. Titles and abstracts should concisely communicate the main findings of the study, and the background, rationale, results and conclusions should be clearly explained in the manuscript in a manner accessible to a broad cell biology audience. Nature Cell Biology uses British spelling.

MANUSCRIPT FORMAT – please follow the guidelines listed in our Guide to Authors regarding manuscript formats at Nature Cell Biology.

TITLE – should be no more than 100 characters including spaces, without punctuation and avoiding technical terms, abbreviations, and active verbs..

AUTHOR NAMES – should be given in full.

AUTHOR AFFILIATIONS – should be denoted with numerical superscripts (not symbols) preceding the names. Full addresses should be included, with US states in full and providing zip/post codes. The corresponding author is denoted by: "Correspondence should be addressed to [initials]."

ABSTRACT AND MAIN TEXT – please follow the guidelines that are specific to the format of your manuscript, as listed in our Guide to Authors (http://www.nature.com/ncb/pdf/ncb_gta.pdf) Briefly, Nature Cell Biology Articles, Resources and Technical Reports have 3500 words, including a 150 word abstract, and the main text is subdivided in Introduction, Results, and Discussion sections. Nature Cell Biology Letters have up to 2500 words, including a 180 word introductory paragraph (abstract), and the text is not subdivided in sections.

ACKNOWLEDGEMENTS – should be kept brief. Professional titles and affiliations are unnecessary. Grant numbers can be listed.

AUTHOR CONTRIBUTIONS – must be included after the Acknowledgements, detailing the contributions of each author to the paper (e.g. experimental work, project planning, data analysis etc.). Each author should be listed by his/her initials.

FINANCIAL AND NON-FINANCIAL COMPETING INTERESTS – the authors must include one of three declarations: (1) that they have no financial and non-financial competing interests; (2) that they have financial and non-financial competing interests; or (3) that they decline to respond, after the Author Contributions section. This statement will be published with the article, and in cases where financial and non-financial competing interests are declared, these will be itemized in a web supplement to the article. For further details please see https://www.nature.com/licenceforms/nrg/competing-interests.pdf.

REFERENCES – are limited to a total of 70 for Articles, Resources, Technical Reports; and 40 for Letters. This includes references in the main text and Methods combined. References must be numbered sequentially as they appear in the main text, tables and figure legends and Methods and must follow the precise style of Nature Cell Biology references. References only cited in the Methods should be numbered consecutively following the last reference cited in the main text. References only associated with Supplementary Information (e.g. in supplementary legends) do not count toward the total reference limit and do not need to be cited in numerical continuity with references in the main text. Only published papers can be cited, and each publication cited should be included in the numbered reference list, which should include the manuscript titles. Footnotes are not permitted.

METHODS – Nature Cell Biology publishes methods online. The methods section should be provided as a separate Word document, which will be copyedited and appended to the manuscript PDF, and incorporated within the HTML format of the paper.

Methods should be written concisely, but should contain all elements necessary to allow interpretation and replication of the results. As a guideline, Methods sections typically do not exceed 3,000 words. The Methods should be divided into subsections listing reagents and techniques. When citing previous methods, accurate references should be provided and any alterations should be noted. Information must be provided about: antibody dilutions, company names, catalogue numbers and clone numbers for monoclonal antibodies; sequences of RNAi and cDNA probes/primers or company names and catalogue numbers if reagents are commercial; cell line names, sources and information on cell line identity and authentication. Animal studies and experiments involving human subjects must be reported in detail, identifying the committees approving the protocols. For studies involving human subjects/samples, a statement must be included confirming that informed consent was obtained. Statistical analyses and information on the reproducibility of experimental results should be provided in a section titled “Statistics and Reproducibility”.

All Nature Cell Biology manuscripts submitted on or after March 21 2016 must include a Data availability statement at the end of the Methods section. For Springer Nature policies on data availability see http://www.nature.com/authors/policies/availability.html; for more information on this particular policy see http://www.nature.com/authors/policies/data/data-availability-statements-data-citations.pdf. The Data availability statement should include:

• Accession codes for primary datasets (generated during the study under consideration and designated as "primary accessions") and secondary datasets (published datasets reanalysed during the study under consideration, designated as "referenced accessions"). For primary accessions data should be made public to coincide with publication of the manuscript. A list of data types for which submission to community-endorsed public repositories is mandated (including sequence, structure, microarray, deep sequencing data) can be found here http://www.nature.com/authors/policies/availability.html#data.

• Unique identifiers (accession codes, DOIs or other unique persistent identifier) and hyperlinks for datasets deposited in an approved repository, but for which data deposition is not mandated (see here for details http://www.nature.com/sdata/data-policies/repositories).

• At a minimum, please include a statement confirming that all relevant data are available from the authors, and/or are included with the manuscript (e.g. as source data or supplementary information), listing which data are included (e.g. by figure panels and data types) and mentioning any restrictions on availability.

• If a dataset has a Digital Object Identifier (DOI) as its unique identifier, we strongly encourage including this in the Reference list and citing the dataset in the Methods.

We recommend that you upload the step-by-step protocols used in this manuscript to protocols.io. More details can found at https://www.protocols.io/help/publish-articles.

DISPLAY ITEMS – main display items are limited to 6-8 main figures and/or main tables for Articles, Resources, Technical Reports; and 5 main figures and/or main tables for Letters. For Supplementary Information see below.

FIGURES – Colour figure publication costs $600 for the first, and $300 for each subsequent colour figure. All panels of a multi-panel figure must be logically connected and arranged as they would appear in the final version. Unnecessary figures and figure panels should be avoided (e.g. data presented in small tables could be stated briefly in the text instead).

All imaging data should be accompanied by scale bars, which should be defined in the legend.
Cropped images of gels/blots are acceptable, but need to be accompanied by size markers, and to retain visible background signal within the linear range (i.e. should not be saturated). The boundaries of panels with low background have to be demarked with black lines. Splicing of panels should only be considered if unavoidable, and must be clearly marked on the figure, and noted in the legend with a statement on whether the samples were obtained and processed simultaneously. Quantitative comparisons between samples on different gels/blots are discouraged; if this is unavoidable, it should only be performed for samples derived from the same experiment with gels/blots were processed in parallel, which needs to be stated in the legend.

Figures should be provided at approximately the size that they are to be printed at (single column is 86 mm, double column is 170 mm) and should not exceed an A4 page (8.5 x 11"). Reduction to the scale that will be used on the page is not necessary, but multi-panel figures should be sized so that the whole figure can be reduced by the same amount at the smallest size at which essential details in each panel are visible. In the interest of our colour-blind readers we ask that you avoid using red and green for contrast in figures. Replacing red with magenta and green with turquoise are two possible colour-safe alternatives. Lines with widths of less than 1 point should be avoided. Sans serif typefaces, such as Helvetica (preferred) or Arial should be used. All text that forms part of a figure should be rewritable and removable.

We accept files from the following graphics packages in either PC or Macintosh format:

- For line art, graphs, charts and schematics we prefer Adobe Illustrator (.AI), Encapsulated PostScript (.EPS) or Portable Document Format (.PDF). Files should be saved or exported as such directly from the application in which they were made, to allow us to restyle them according to our journal house style.

- We accept PowerPoint (.PPT) files if they are fully editable. However, please refrain from adding PowerPoint graphical effects to objects, as this results in them outputting poor quality raster art. Text used for PowerPoint figures should be Helvetica (preferred) or Arial.

- We do not recommend using Adobe Photoshop for designing figures, but we can accept Photoshop generated (.PSD or .TIFF) files only if each element included in the figure (text, labels, pictures, graphs, arrows and scale bars) are on separate layers. All text should be editable in 'type layers' and line-art such as graphs and other simple schematics should be preserved and embedded within 'vector smart objects' - not flattened raster/bitmap graphics.

- Some programs can generate Postscript by 'printing to file' (found in the Print dialogue). If using an application not listed above, save the file in PostScript format or email our Art Editor, Allen Beattie for advice (a.beattie@nature.com).

Regardless of format, all figures must be vector graphic compatible files, not supplied in a flattened raster/bitmap graphics format, but should be fully editable, allowing us to highlight/copy/paste all text and move individual parts of the figures (i.e. arrows, lines, x and y axes, graphs, tick marks, scale bars etc.). The only parts of the figure that should be in pixel raster/bitmap format are photographic images or 3D rendered graphics/complex technical illustrations.

All placed images (i.e. a photo incorporated into a figure) should be on a separate layer and independent from any superimposed scale bars or text. Individual photographic images must be a minimum of 300+ DPI (at actual size) or kept constant from the original picture acquisition and not decreased in resolution post image acquisition. All colour artwork should be RGB format.

FIGURE LEGENDS – must not exceed 350 words for each figure to allow fit on a single printed NCB page together with the figure. They must include a brief title for the whole figure, and short descriptions of each panel with definitions of the symbols used, but without detailing methodology.

TABLES – main tables should be provided as individual Word files, together with a brief title and legend. For supplementary tables see below.

SUPPLEMENTARY INFORMATION – Supplementary information is material directly relevant to the conclusion of a paper, but which cannot be included in the printed version in order to keep the manuscript concise and accessible to the general reader. Supplementary information is an integral part of a Nature Cell Biology publication and should be prepared and presented with as much care as the main display item, but it must not include non-essential data or text, which may be removed at the editor's discretion. All supplementary material is fully peer-reviewed and published online as part of the HTML version of the manuscript. Supplementary Figures and Supplementary Notes are appended at the end of the main PDF of the published manuscript.

Supplementary items should relate to a main text figure, wherever possible, and should be mentioned sequentially in the main manuscript, designated as Supplementary Figure, Table, Video, or Note, and numbered continuously (e.g. Supplementary Figure 1, Supplementary Figure 2, Supplementary Table 1, Supplementary Table 2 etc.).

Unprocessed scans of all key data generated through electrophoretic separation techniques need to be presented in a supplementary

figure that should be labelled and numbered as the final supplementary figure, and should be mentioned in every relevant figure legend. This figure does not count towards the total number of figures and is the only figure that can be displayed over multiple pages, but should be provided as a single file, in PDF or TIFF format. Data in this figure can be displayed in a relatively informal style, but size markers and the figures panels corresponding to the presented data must be indicated.

The total number of Supplementary Figures (not including the "unprocessed scans" Supplementary Figure) should not exceed the number of main display items (figures and/or tables (see our Guide to Authors and March 2012 editorial http://www.nature.com/ncb/authors/submit/index.html#suppinfo; http://www.nature.com/ncb/journal/v14/n3/index.html#ed). No restrictions apply to Supplementary Tables or Videos, but we advise authors to be selective in including supplemental data.

Each Supplementary Figure should be provided as a single page and as an individual file in one of our accepted figure formats and should be presented according to our figure guidelines (see above). Supplementary Tables should be provided as individual Excel files. Supplementary Videos should be provided as .avi or .mov files up to 50 MB in size. Supplementary Figures, Tables and Videos much be accompanied by a separate Word document including titles and legends.

GUIDELINES FOR EXPERIMENTAL AND STATISTICAL REPORTING

REPORTING REQUIREMENTS – To improve the quality of methods and statistics reporting in our papers we have recently revised the reporting checklist we introduced in 2013. We are now asking all life sciences authors to complete two items: an Editorial Policy Checklist (found here https://www.nature.com/authors/policies/Policy.pdf) that verifies compliance with all required editorial policies and a reporting summary (found here https://www.nature.com/authors/policies/ReportingSummary.pdf) that collects information on experimental design and reagents. These documents are available to referees to aid the evaluation of the manuscript. Please note that these forms are dynamic 'smart pdfs' and must therefore be downloaded and completed in Adobe Reader. We will then flatten them for ease of use by the reviewers. If you would like to reference the guidance text as you complete the template, please access these flattened versions at http://www.nature.com/authors/policies/availability.html.

STATISTICS – Wherever statistics have been derived the legend needs to provide the n number (i.e. the sample size used to derive statistics) as a precise value (not a range), and define what this value represents. Error bars need to be defined in the legends (e.g. SD, SEM) together with a measure of centre (e.g. mean, median). Box plots need to be defined in terms of minima, maxima, centre, and percentiles. Ranges are more appropriate than standard errors for small data sets. Wherever statistical significance has been derived, precise p values need to be provided and the statistical test used needs to be stated in the legend. Statistics such as error bars must not be derived from n<3. For sample sizes of n<5 please plot the individual data points rather than providing bar graphs. Deriving statistics from technical replicate samples, rather than biological replicates is strongly discouraged. Wherever statistical significance has been derived, precise p values need to be provided and the statistical test stated in the legend.

Information on how many times each experiment was repeated independently with similar results needs to be provided in the legends and/or Methods for all experiments, and in particular wherever representative experiments are shown.

We strongly recommend the presentation of source data for graphical and statistical analyses as a separate Supplementary Table, and request that source data for all independent repeats are provided when representative experiments of multiple independent repeats, or averages of two independent experiments are presented. This supplementary table should be in Excel format, with data for different figures provided as different sheets within a single Excel file. It should be labelled and numbered as one of the supplementary tables, titled "Statistics Source Data", and mentioned in all relevant figure legends.

--------- Please don't hesitate to contact NCB@nature.com should you have queries about any of the above requirements ---------

Version 1:

Decision Letter:

Our ref: NCB-A56697A

13th March 2026

Dear Dr. Jha,

Thank you for submitting your revised manuscript "Transmembrane picket CD44 restricts EGFR mobility to generate a novel spatial arrangement of cytoskeletal signaling modules driving bleb-based migration" (NCB-A56697A). It has now been seen by the original referees and their comments are below. The reviewers find that the paper has improved in revision, and therefore we'll be happy in principle to publish it in Nature Cell Biology, pending minor revisions to satisfy the referees' final requests and to comply with our editorial and formatting guidelines.

The current version of your manuscript is in a PDF format, so please email us a copy of the file in an editable format (Microsoft Word or LaTex)-- we can not proceed with PDFs at this stage.

We are now performing detailed checks on your paper and will send you a checklist detailing our editorial and formatting requirements in about a week. Please do not upload the final materials and make any revisions until you receive this additional information from us.

Thank you again for your interest in Nature Cell Biology Please do not hesitate to contact me if you have any questions.

Sincerely,

Daryl

Daryl Jason Verzosa David, PhD

Senior Editor, Nature Cell Biology
Advisory Editor, npj Biological Physics and Mechanics
Nature Portfolio

Heidelberger Platz 3, 14197 Berlin, Germany
Email: daryl.david@nature.com
ORCID: https://orcid.org/0000-0002-9253-4805

Reviewer #1 (Remarks to the Author):

Jha et al. present an elegant and strong publication where they show that the same polarity machinery used in lamellipodia/mesenchymal migration can be repurposed and used to drive bleb-based migration under confinement. Their experimental work has a strong solid foundation that is supported by modelling. In the initial submission, we did not have strong concerns with the mechanism nor the evidence they provide for their work. In the response to the reviewer comments, the authors have tried to address to the best of their abilities and technical capabilities all the comments not only from our reviews, but from most of the reviewer's comments. All and all, we are happy with the current state of the paper and believe this would be a great addition for NCB.

Jha et al have done a great effort to revised their manuscript and completed most (if not all) the necessary changes that the reviewers asked. With that said some final minor comments to take into account:

Comment 1: Strong proof of EGFR direct effect in bleb-based migration
The authors corrected the negative migration persistence from the original plots. The original concern over EGFR function was that only pharmacological inhibitions showed a strong reduction in the migration phenotype of confined cells. These treatments lead to off-targets effects that may interfere with migration independent of EGFR. The authors have strengthened their proof of EGFR and its effect in bleb-based migration by adding an independent siRNA that shows a stronger effect. Along with these experiments, western blots show the protein depletion of EGFR for siRNA efficiency.
The authors have re-plotted, and in line with our comments and other reviewers, have added population level speeds using cumulative frequency and total mean squared displacement, strengthening the results and supporting the claim that EGFR drives bleb-based migration in confined cells.
Furthermore, we acknowledge it is not a trivial task to generate KO of EGFR and the experimental limitations this brings. Therefore, the current figure has strengthened the proof that EGFR and Pi3K pathways influence bleb-based migration in confinement and we feel this satisfies our initial concerns.

Comment 2: Model strength
The authors have further explained the use of the model and how it helped them identify that retrograde actin flow cannot explain the existence of this EGFR gradient. The initial model predicted that changing actin flows (as in Fig Ext 2 K) would affect the gradient, but upon experimental perturbations this did not change the gradient of EGFR, meaning this was independent of actin retrograde flow. Later on, the model showed that if the actin retrograde flow was left constant, the change in corralling would change the EGFR gradient (as in Fig 5 A).
The authors have now added a more detail explanation of the mathematical model importance and strengthened their evidence on corralling as the driver of the EGFR gradient with both experimental and modelling approaches. We feel the current text explanations and changes strengthened the model use and is now clearer why it was needed.

Comment 3: 3D bleb migration gradient proof lacking
The authors have raised concerns regarding the technical complexity of performing 3D imaging experiments for the proof that the current 2D phenomena can be interpolated to 3D migration. We acknowledge a full characterization of Rho/Rac sensors is technically challenging. Nevertheless, the current figure (5N) does not fully show the gradient observed in 2D, there is no quantification and the sample size is a single blebbing cell in a 3D ECM. Furthermore, the image is a single slice that lacks any volumetric information regarding the 3D distribution of the stained proteins.
Since the authors claim this phenomenon should be explored in 3D in a different publication work, we recommend strengthen the discussion with further evidence (earlier referenced) where upstream/downstream biochemical molecules are explored.

Comment 4: Gradients of CD44-Ezrin in blebs and differences in gradient
We appreciate that the authors have modified their analysis and quantified through a normalization that better shows the image represented. In addition, the authors have compared the localization of p-ERMs compared to actin within the bleb. This comparison has now clarified that the differences on gradient between CD44 and ERMs arise from the un-specificity of labelling all Ezrin forms (active and inactive). The pERM staining clarifies the increase localization in the bleb bottom where most actin localizes.
As a technical comment, I do not understand why the authors did not compare the pERM to CDC44 or EGFR in the same cell and the sample size seems to be of a single blebbing cell.
Even though the sample size for this work is small, the addition of the p-ERM clarifies the earlier concerns we had and further reiterates how important it is to compare active ERMs compared to its bulk. We are satisfied with the new data.

Comment 5: Loss of EGFR/ Rac and CD44 gradients with optogenetic inhibition is affecting more persistence than speed.
This comment seems to be a concerned shared across all reviewers so it was important to address it. The authors have now clarified the discrepancies in these results. Now, there are figures to compare population level changes using MSD, frequency and distribution of

phenotypes. And specifically look at the speed and persistence of cells that successfully form stable blebs. Now this allows us to compare the population and migration level differences. This has changed across the paper and we greatly appreciate the clarity that this brings for the experiment.
Since there are no significant differences in the mean speed but as mentioned Maiuri et al 2012, the distribution of the speed is reduced upon the different treatments, we recommend the authors to state this in the main text since very clearly even though the mean significance does not change, distribution seem to be a better metric to compare the changes in speed.

Reviewer #2 (Remarks to the Author):

The authors have largely addressed my comments. While I appreciate the response in the rebuttal to the comment about how normally cells in confinement still have access to ligands in the ECM, I really think this should be included in the discussion section of the manuscript. It provides important context for these results and extends their relevance beyond the artificial low-adhesive environment used in this work.

Patrick Oakes

Reviewer #2 (Remarks on Protocol(s)):

The troubleshooting section of the protocol is empty. It would be useful to provide more details in this section about potential issues that can come up.

Reviewer #3 (Remarks to the Author):

Jha and colleagues have done a nice job of revising this manuscript per my comments. I still think they should better discuss the likely much more complex nature of signaling that drives this fascinating and potentially clinically important phenotype (and missed a bit of an opportunity to bring a wider audience to this important fundamental science). These melanoma cells have a constitutively active Raf mutation and therefore abnormally high levels of MAPK signaling and likely well-known negative feedback - especially given their previous work on the role of ERK in bleb-based migration in these cells. But I will not stand in the way of publishing this otherwise lovely work.

Version 2:

Decision Letter:

Dear Dr Jha,

I am pleased to inform you that your manuscript, "CD44 restricts EGFR mobility to polarize cytoskeletal signaling modules driving bleb-based migration.", has now been accepted for publication in Nature Cell Biology.

Thank you for sending us the final manuscript files to be processed for print and online production, and for returning the manuscript checklists and other forms. Your manuscript will now be passed to our production team who will be in contact with you if there are any questions with the production quality of supplied figures and text.

Over the next few weeks, your paper will be copyedited to ensure that it conforms to Nature Cell Biology style. Once your paper is typeset, you will receive an email with a link to choose the appropriate publishing options for your paper and our Author Services team will be in touch regarding any additional information that may be required.

After the grant of rights is completed, you will receive a link to your electronic proof via email with a request to make any corrections within 48 hours. If, when you receive your proof, you cannot meet this deadline, please inform us at rjsproduction@springernature.com immediately.

You will not receive your proofs until the publishing agreement has been received through our system.

Due to the importance of these deadlines, we ask that you please let us know now whether you will be difficult to contact over the next month. If this is the case, we ask you provide us with the contact information (email, phone and fax) of someone who will be able to check the proofs on your behalf, and who will be available to address any last-minute problems.

If you have any questions about our publishing options, costs, Open Access requirements, or our legal forms, please contact ASJournals@springernature.com

Once your paper has been scheduled for online publication, the Nature press office will be in touch to confirm the details. An online order form for reprints of your paper is available at https://www.nature.com/reprints/author-reprints.html. All co-authors, authors' institutions and authors' funding agencies can order reprints using the form appropriate to their geographical region.

Publication is conditional on the manuscript not being published elsewhere and on there being no announcement of this work to any media outlet until the online publication date in Nature Cell Biology.

You may wish to make your media relations office aware of your accepted publication, in case they consider it appropriate to organize some internal or external publicity. Once your paper has been scheduled you will receive an email confirming the publication details. This is normally 3-4 working days in advance of publication. If you need additional notice of the date and time of publication, please let the production team know when you receive the proof of your article to ensure there is sufficient time to coordinate. Further information on our embargo policies can be found here: https://www.nature.com/authors/policies/embargo.html

Please note that *Nature Cell Biology* is a Transformative Journal (TJ). Authors may publish their research with us through the traditional subscription access route or make their paper immediately open access through payment of an article-processing charge (APC). Authors will not be required to make a final decision about access to their article until it has been accepted. Find out more about Transformative Journals

Authors may need to take specific actions to achieve compliance with funder and institutional open access mandates. If your research is supported by a funder that requires immediate open access (e.g. according to Plan S principles or the NIH public access policy) then you should select the gold OA route, and we will direct you to the compliant route where possible. Because authors warrant under our subscription licensing terms that they haven't committed to licensing any version of their article under a licence inconsistent with the terms of our agreement – including the applicable embargo period – publication under the subscription model isn't suitable for authors whose funders require no embargo.

To assist our authors in disseminating their research to the broader community, our SharedIt initiative provides you with a unique shareable link that will allow anyone (with or without a subscription) to read the published article. Recipients of the link with a subscription will also be able to download and print the PDF.

If your paper includes color figures, please be aware that in order to help cover some of the additional cost of four-color reproduction, Nature Portfolio charges our authors a fee for the printing of their color figures. Please contact our offices for exact pricing and details.

As soon as your article is published, you will receive an automated email with your shareable link.

If you have not already done so, we strongly recommend that you upload the step-by-step protocols used in this manuscript to protocols.io (https://protocols.io), an open online resource that allows researchers to share their detailed experimental know-how. All uploaded protocols are made freely available and are assigned DOIs for ease of citation. Protocols and Nature Portfolio journal papers in which they are used can be linked to one another, and this link is clearly and prominently visible in the online versions of both. Authors who performed the specific experiments can act as primary authors for the Protocol as they will be best placed to share the methodology details, but the Corresponding Author of the present research paper should be included as one of the authors. By uploading your Protocols onto protocols.io, you are enabling researchers to more readily reproduce or adapt the methodology you use, as well as increasing the visibility of your protocols and papers. You can also establish a dedicated workspace to collect your lab Protocols. Further information can be found at https://www.protocols.io/help/publish-articles.

You can use a single sign-on for all your accounts, view the status of all your manuscript submissions and reviews, access usage statistics for your published articles and download a record of your refereeing activity for the Nature Portfolio.

Nature Cell Biology encourages authors presenting evidence for cell, biological, molecular, and genetic interactions to consider communicating these findings using Biofactoid (https://biofactoid.org/). This tool helps users share a searchable representation of interactions (e.g. binding, gene expression, post-translational modification) between genes, gene products, or chemicals. Information added to Biofactoid, with author attribution, is shared on social media and public databases, such as Pathway Commons, where it can be discovered and analyzed in the context of a large and growing corpus of knowledge.

Please feel free to contact us if you have any questions.

With kind regards,
Daryl

Daryl Jason Verzosa David, PhD

Senior Editor, Nature Cell Biology
Advisory Editor, npj Biological Physics and Mechanics
Nature Portfolio

Heidelberger Platz 3, 14197 Berlin, Germany
Email: daryl.david@nature.com
ORCID: https://orcid.org/0000-0002-9253-4805

Click here if you would like to recommend Nature Cell Biology to your librarian
http://www.nature.com/subscriptions/recommend.html#forms

** Visit the Springer Nature Editorial and Publishing website at www.springernature.com/editorial-and-publishing-jobs for more information about our career opportunities. If you have any questions please click here.**

Open Access This Peer Review File is licensed under a Creative Commons Attribution 4.0 International License, which permits use, sharing, adaptation, distribution and reproduction in any medium or format, as long as you give appropriate credit to the original author(s) and the source, provide a link to the Creative Commons license, and indicate if changes were made.
In cases where reviewers are anonymous, credit should be given to 'Anonymous Referee' and the source.
The images or other third party material in this Peer Review File are included in the article's Creative Commons license, unless indicated otherwise in a credit line to the material. If material is not included in the article's Creative Commons license and your intended use is not permitted by statutory regulation or exceeds the permitted use, you will need to obtain permission directly from the copyright holder.
To view a copy of this license, visit https://creativecommons.org/licenses/by/4.0/

Reviewers' Comments:

Reviewer #1 (Remarks to the Author):

Metastatic tumor cells adopt bleb-driven mode of protrusion when ECM ligands are unavailable or integrin signaling is suppressed. However, the precise molecular mechanisms underlying bleb-based migration remain unclear; in particular, the role of polarized receptor signaling in symmetry breaking and contractility gradients across blebs remains unknown. In this paper, Jha and colleagues have combined a series of molecular sensors, high-resolution imaging, and genetic manipulations to propose a novel molecular mechanism for establishing cell polarity in bleb-driven migration. The authors focused on the formation and maintenance of elongated leader blebs, which were shown to be correlated with efficient and persistent migration. Notably, perturbation of EGFR signaling through inhibition or knockdowns results in a decrease of leader bleb population and consequent reduction of migratory range. By using a series of molecular sensors, the authors unveiled a rear-to-front gradient of EGFR localization and activity in leader which is transmitted to downstream Rac1 signaling. In contrast, RhoA is polarized at the bleb tip; thus, bleb-based migration exhibits a reverse Rac1/RhoA polarity compared to mesenchymal cell migration. In accordance with the previous findings, disruption of EGFR/Rac1 polarity through optogenetic manipulation results in reduction of leader bleb length, actin disassembly and bleb retraction. FRAP experiments combined with mathematical modelling revealed that the EGFR gradient originates from a ”corralling” of EGFR at the bleb rear, where it resists anterograde membrane flow. This “corralling” appears to be driven by accumulation of CD44, ezrin/moesin, and membrane-proximal actin at the rear of the bleb, resulting in a decrease of EGFR mobility. Further depletion of CD44 or ezrin/moesin abolishes rear-to-front EGFR gradients hence decreasing polarity. In the case of CD44 depletion, the Rac1 gradient is also flattened, ultimately inhibiting leader bleb formation and reducing migratory range.

In summary, the authors have unveiled a novel molecular mechanism for establishing rear-to-front EGFR and Rac1 polarity in bleb-driven migration, opposite to what is observed in mesenchymal cell migration. This polarity appears to be driven by a membrane “corralling” mechanism where CD44, ezrin-moesin and membrane-proximal actin act synergistically to restrict EGFR mobility. Loss of this gradient through various perturbations ultimately affects leader bleb stability and migration persistence. Overall, I consider that the conclusions of this study are well substantiated, and the proposed molecular mechanism is thoroughly explained and fits with the experimental data. I personally find that as its currently written this manuscript does not convey what I think the most significant finding of this work, which is the unveiling of a reverse signaling polarity network in bleb-driven migration; in light also of my first minor point below I would change the order of figures but leave that for the authors to decide.

Either way, I believe that only a few points need to be addressed to bring this study to completion:
1) Figure 1 directs the reader to a possible role of EGFR in bleb-driven migration, for which the data is not fully conclusive. The role of EGFR is based on its inhibition by drugs (which are not totally specific) while the siRNA appears to have quite an effect even in the control case, since the control iRNA from Figure 1K has a much lower persistence than the DMSO controls (Figure 1H), suggesting a possible toxicity per se. In fact, the persistence appears negative, is there an error in that plot? If not, could the authors clarify how these values can be achieved? In any case, as the authors acknowledge, the difference between control and EGFR-KD is not significant, which does not fully support a key role of EGFR in leader bleb formation and persistent migration. I am not questioning the sufficiency of EGFR (shown in figure 3D-E) but the

necessity. Is this something that lead-bleb migrating cells need? I would encourage the authors to make a cleaner KD or ADS or KO.

We thank the reviewer for this thoughtful comment.

Error in the persistence plot: We appreciate the reviewer pointing out the discrepancy in Figure 1H. The negative persistence values for siRNA controls arose from an exporting error while transferring graphs between applications. This has now been corrected and the new figure corresponds to Figure 1O. As noted, persistence of control siRNA cells is slightly lower than DMSO-treated controls, which we attribute to mild toxicity from the chemical transfection procedure.

siRNA knockdown controls: We agree that the difference in persistence between EGFR-KD and control siRNA was not striking in the initial dataset, since we initially only quantified motility for cells that form a leader bleb. Indeed, we have found that once a cell actually does form a stabilized leader bleb, it is generally capable of efficient migration, independent of the treatment or condition. However, to address our weak conclusion based on a single siRNA targeting a unique sequence EGFR, we repeated experiments with an independent siRNA targeting a different unique sequence in EGFR and a different control siRNA. These data confirm that EGFR-KD causes a significant reduction in leader bleb formation and a corresponding increase in the population of cells displaying only small transient blebs (Figure 1L, 1M), with also a drastic reduction in the efficiency of migration of all the cells (Figure 1N). We also now measured the speed of all the cells and saw a significant population of cells moving slowly, as demonstrated in cumulative frequency plots of cell speeds (Figure 1P), strengthening the conclusion that EGFR depletion disrupts leader bleb–based migration.

Specificity of pharmacological inhibition: We agree with the reviewer that small-molecule inhibitors are not fully specific. EGFR (HER1) is indeed one of several receptor tyrosine kinases (RTKs) capable of influencing MAPK signaling, along with HER2, c-Met, and FGFR. To further address this concern, we tested two additional inhibitors: Lapatinib (HER1/2 dual inhibitor) and Gefitinib (EGFR-specific inhibitor). Like our results with erlotinib, both treatments resulted in a strong reduction in leader bleb formation and a dramatic increase in cells with small blebs (Figure 1F and 1G) and severely impacted the efficiency of migrating cells (Figure 1H). We did not make a EGFR KO because often that leads to very low cell count due to effects on cell proliferation (Orofiamma et al. 2022; Ludwig et al. 2023)

Taken together, evidence from two independent siRNAs, multiple pharmacological inhibitors, and functional correlation with bleb morphology, support our conclusion that EGFR is one of the primary RTK required for stabilization of leader blebs and persistent migration under confinement.

The text has been modifies as follows:

"…targeting either EGFR tyrosine kinase activity with erlotinib (50nM), gefitinib (1µM), lapatinib (1µM) or blocking its downstream effector phosphatidylinositol 3-kinase (Pi3K) with wortmannin (100nM, Extended Figure 1M), strongly reduced leader bleb formation, cell migratory range and speed of the cells (Figure 1F-H,1J; Movie 3). The few cells that did make leader blebs under erlotinib or wortmannin treatment showed significantly reduced migration persistence, but no difference in migration speed compared to leader bleb cells in DMSO control (Figure 1I and 1K). Similarly, knocking down EGFR (EGFR-KD) by two independent siRNAs (Extended Figure 1N) strongly inhibited the formation of leader blebs, cell migratory range and speed compared to non-targeting siRNAs (NT), (Figure 1L-M, 1P; Movie 4J). Due to a drastic reduction in the number of cells exhibiting leader bleb morphology, especially in cells treated with siRNA2, EGFR-KD reduced both migration persistence and speed (Figure 1O, 1Q). Thus, EGF signaling through PI3K is required for stabilizing and maintaining polarized, elongated stable leader bleb morphology to drive persistent migration and promote migratory range under low-adhesive confinement."

• In Figure 4, the authors explain how “Experiment and modeling indicate neither cortical nor membrane flow mediate GFR gradient formation in leader blebs”. While the experimental data clearly supports the claim, the relevance of the mathematical model for the results is unclear. Could the authors provide further clarification on the model and its applications in the discussion section?

We thank the reviewer for recognizing our shortcoming in communicating how the model contributed to our study. The mathematical model was instrumental in providing conceptual guidance beyond our own intuition and what we could directly observe experimentally. Specifically, the model predicted that both EGFR and bulk membrane should undergo a net anterograde movement from the bleb base toward the tip, despite the presence of strong retrograde actin flow. This was very counterintuitive to us, and prompted us to directly test EGFR and membrane marker dynamics using photobleaching experiments, which confirmed anterograde flow of both, but revealed that EGFR movement was slowed relative to the bulk membrane (Figure 4H). Thus, the model helped us formulate and test the hypothesis that restricted mobility, rather than cytoskeletal or membrane flow, underlies EGFR gradient formation.

The text has been modified as follows to clarify this:

“To differentiate between the hypotheses of advection and local restriction of free mobility as possible mechanisms of maintaining a rear-to-front gradient of EGFR in leader blebs, we used a combination of mathematical modelling and experiment. The model was based on the assumptions that EGFR and membrane synthesis occur in the cell body, the bleb has a geometry based on our average measurements (Extended Figure 2C and 2D), EGFR has an apparent diffusivity based on FRAP measurements((D_p), Extended Figure 2E and 2F), a forward advection velocity from bleb base to tip (V_p), a negative retrograde cytoskeletal flow velocity ((V_f), Extended Figure 2G-2J), and a turnover (degradation or endocytic removal) frequency (membrane k_m and protein k_p), each of which can potentially vary with position along the bleb length (L) (Figure 4A). An important insight gained from initial consideration that EGFR and membrane synthesis occur in the cell body and that both bulk membrane and EGFR must both undergo a net anterograde movement from the bleb base towards the tip (See Supplementary text). This was surprising, and in opposition to the notion that advective movement via retrograde cytoskeletal flow would mediate the formation of the rear-to-front EGFR gradient.”

We have also revised the Discussion, clarifying how modeling complemented the experimental data by making a prediction that was very counterintuitive to us in the first ppg of the discussion:

“Our mathematical modeling surprised us by predicting net anterograde EGFR movement from bleb base to tip, despite the presence of strong actin retrograde flow.”

• In Figure 5, the authors demonstrate that cells migrating on 3D fibrillar ECM, CD44 and EGFR also accumulate at the rear. To understand whether the rear-to-front polarity is fully recapitulated in 3D environments, Rac1 and RhoA sensors could be imaged in the same conditions, as well as ezrin and moesin. To facilitate the comprehension of this point, I would propose to include the EGFR imaging together with the suggested Rac1 and RhoA sensor imaging in Figure 2, while the CD44 and suggested ezrin/moesin images could remain in Figure 5. The authors could further strengthen the robustness of this phenotype by adding quantifications of gradients in 3D.

We thank the reviewer for this thoughtful suggestion. We have similarly contemplated efforts to address this by imaging Rac1 and RhoA activity sensors in cells migrating within 3D fibrillar ECM. However, these experiments require generation of stable biosensor-expressing cell lines and the development of advanced

3D image analysis quantification approaches, since simple line-scan measurements on a single optical plane as is done on cells under confinement in 2D where the plasma membrane is pressed against the coverslip, are not sufficient in 3D. We therefore believe this represents an important but more extensive line of investigation that will be better addressed in a future study dedicated to dissecting signaling gradients in bleb-based migration in 3D microenvironments and *in vivo*.

For the current manuscript, we have focused on CD44 and EGFR distributions in 3D using immunostaining (Figure 5), which already qualitatively demonstrates that these receptors form rear-to-front gradients similar to those observed in confinement assays in 2D. Furthermore, recent studies support our findings by showing that PI3K localizes to the bleb base (Weems et al. 2023) and that additional components such as septins and PIP2 (Driscoll et al. 2019) are spatially organized within blebs in 3D. These observations strengthen the physiological relevance of the polarity we describe. We have clarified this limitation in the Discussions and noted that extending our analysis and findings to 3D models and *in vivo* will be an exciting direction for future work. This is noted in the last sentence of the paper:

"How common these phenomena are across different solid tumors and how they change with disease progression remain important areas for further exploration."

• In Figure 5D, the rear-to-front ezrin and CD44 gradients appear to be of different magnitudes according to the quantification plot, with CD44 exhibiting a much higher intensity at the rear of the bleb compared to ezrin. However, this is not clearly displayed in the representative image, where they appear to be similar. Could the authors 1) provide representative images which better reflect the quantifications and 2) address in the discussion why the gradients would have different degrees of intensity?

We thank the reviewer for this valuable comment.

Representative images and quantification display: To better represent the gradients, we have now normalized the intensity values in the quantification plots (Figure 5J). In addition, we have labeled the region which is bleb and cell body for better visualization.

Different gradient magnitudes: CD44 is a transmembrane protein that is immobilized at the bleb rear through its cytoplasmic tail and ERM linkages to the actin cortex, whereas ezrin is a cytosolic protein that has a FERM domain to bind the plasma membrane and binds to the cytoskeleton in a phosphorylation-dependent manner. Examination of the figure (Figure 5J and 5K) shows that CD44 and actin share very similar localizations, suggesting that the bulk of the CD44 in the cell is trapped at the bleb rear by association with the membrane-proximal cytoskeleton, likely through ERM proteins. In contrast, ezrin itself may have a cytosolic or non-cytoskeleton bound fraction in the rest of the bleb. Furthermore, trapping of CD44 by the membrane-proximal cytoskeleton could also be mediated by other members of the ERM protein family, including moesin or radixin.

However, to address the reviewer's concern, we have now performed immunostaining of cells under confinement with phospho-ERM (Fig 5L, 5M), which revealed a very similar localization as actin showing the cytoskeleton-bound fraction of ERM proteins at the same location where bulk CD44 is trapped (Figure 5F and 5G). We have included this new finding in the text as follows:

"Immunostaining for CD44, ezrin, or active, phosphorylated ERM proteins (phospho-ERM) in confined cells revealed a concentration of CD44 in the cell body and both ezrin and phospho-ERM concentrated in the leader bleb. Line-scan analysis of leader blebs showed that while ezrin was localized throughout the bleb, CD44 and phospho-ERM formed rear-to-front gradients (Figure 5J-5M). "

The loss of EGFR/Rac1 gradients through direct genetic perturbations or CD44 depletion has a more striking effect in migration persistence compared to speed. Indeed, migration speed is not affected upon EGFR pharmacological inhibition as well as upon depletion of CD44. While I acknowledge that addressing this discrepancy would likely require a series of experiments outside the scope of this paper, the authors could propose their interpretation about these observations in the discussion.

We thank the reviewer for raising this important point. As noted, our initial analysis did not show a significant difference in migration speed between control cells and those with genetic or drug perturbations of EGFR or CD44 undergoing leader bleb-based migration. This is because once a cell forms a leader bleb, as long as that bleb is stabilized and elongated, the cell inherently migrates in an efficient manner with high directional persistence (Figure 1D), more efficiently (Extended Figure 1B), and with high speed compared to other morphologies (Extended Figure 1D). **In our initial submission, we only measured speed and persistence during the time that the cell maintains a leader bleb morphology**. We have noted that if the bleb morphology is not fully stable, this will inherently impact migration persistence, because if the bleb destabilizes for a short period, this can be followed by bleb re-establishment of a new motility bout in a new direction (Figure 1D). However, to clarify, **we have now added cumulative frequency plots of the speed and MSD plots of all the cells, independent of their morphological state, for all of our cell migration experiments.** Coupled with the bleb morphology distribution, MSD and cumulative frequency now reports changes in the total population for each perturbation. The persistence and speed plots display only the trajectories of cells that successfully established leader blebs. Within this subpopulation, cells migrate efficiently, consistent with previous reports that once leader blebs form, they provide a robust mechanism for persistent migration, independent of the condition. However, when polarity is disrupted, fewer cells can access this highly efficient state. Thus, the major phenotype is a population-level reduction in the ability of cells to form stabilized leader blebs and thus undergo leader bleb–driven migration, rather than a strong effect on the performance of individual leader bleb cells.

We have modified the text in several places in the Results section to clarify this point:

"Analysis of cell motility showed that serum starvation strongly reduced migratory range for all cells, and the few cells that exhibited leader bleb morphology had reduced migration persistence but no difference in their speed while moving compared to leader bleb cells in serum (Extended Figure 1H-1K)."

and:

"Importantly, addition of EGF to serum-starved cells partially rescued the leader bleb morphology (Extended Figure 1F, 1G; Movie 2), increased the migratory range of all cells, and for leader bleb cells, increased the migration persistence, but had no significant effect on their speed (Extended Figure 1H-1K). Furthermore, targeting either EGFR tyrosine kinase activity with erlotinib (50nM), gefitinib (1µM), lapatinib (1µM) or blocking its downstream effector phosphatidylinositol 3-kinase (Pi3K) with wortmannin (100nM, Extended Figure 1M), strongly reduced leader bleb formation, cell migratory range and speed of the cells (Figure 1F-H,1J; Movie 3). The few cells that did make leader blebs under erlotinib or wortmannin treatment showed significantly reduced migration persistence, but no difference in migration speed compared to leader bleb cells in DMSO control (Figure 1I and 1K). Similarly, knocking down EGFR (EGFR-KD) by two independent siRNAs (Extended Figure 1N) strongly inhibited the formation of leader blebs, cell migratory range and speed compared to non-targeting siRNAs (NT), (Figure 1L-M, 1P; Movie 4J). Due to a drastic reduction in the number of cells exhibiting leader bleb morphology, especially in cells treated with siRNA2, EGFR-KD reduced both migration persistence and speed (Figure 1O, 1Q)".

and:

"Analysis of cell motility parameters showed that siRNAs targeting the 3'UTR of CD44 (CD44-3'KD, Extended Figure 4J) significantly inhibited leader bleb formation (Figure 7C and 7D; Movie 14) and drastically reduced the migratory range of cells in low-adhesive confinement (Figure 7E). However, cells that managed to undergo leader bleb-based migration exhibited persistent migration with similar velocity (Figure 7F, 7H; Movie 15)."

Moreover, previous work has also shown **that changes in the mean speed of migration for a given cell type does not typically reflect a global shift in the distribution, but rather results from broadening of the distribution due to a subpopulation of faster-moving cells** (Maiuri et al. 2012). These analyses show that while average speeds of cells that stabilize leader blebs are not significantly altered, the proportion of highly persistent leader bleb–driven cells is reduced upon EGFR or CD44 perturbation, explaining the stronger effect on persistence compared to speed.

Additional minor points:

• For some of the representative microscopy images, the authors could overlay lines to delimit the cell body and the bleb and make the figure clearer to the readers. For instance, in Figures 5D and 5G this would facilitate the identification of the ezrin and CD44 gradients between the bleb rear and tip.

We agree with the reviewers and thank them for their input. In order to make the figure clearer we have now have added overlay lines to delimit cell body and the bleb when we first show these gradients (Figure 2A). We have also labelled bleb and cell body in Figure 5J, 5L and 5N for clarity.

• The same group already suggested the involvement of ezrin in the stability of leader blebs a decade ago. In the discussion they wrote: "Our demonstration the Eps8 localizes to bleb membranes on a similar timescale as ezrin suggests that their interaction (Zwaenepoel et al., 2012) may be critical to Eps8 cortical recruitment". It would be important to refer to their work when ezrin is first introduced in the manuscript.

We thank the reviewer for this insight and have now cited the work and it reads as follows-

"As CD44 and ERM (Ezrin-Radixin-Moesin) proteins are known to link to the sub-membrane cortical actin meshwork to create membrane corrals that restrict free diffusion of transmembrane proteins in other cell types[36] and ezrin is crucial for bleb stability[19,28], we examined their distributions in leader blebs"

• I would recommend the authors to also refer to Driscoll et al 2024 Dev Cell, as they nicely showed the importance of PI3K signalling for bleb formation and size.

We thank the reviewer and have now cited and discussed the noted work.

"This spatial reorganization of Rho-GTPases and PI3K signaling[70] may allow cancer cells to use the same basic motility building blocks to rapidly shift from mesenchymal to bleb-based migration without the need for activating different transcriptional programs, thus quickly suiting the migratory mode to varying environments encountered on the metastatic journey."

Reviewer #2 (Remarks to the Author):

In this manuscript Jha and colleagues set out to investigate the molecular mechanisms that underlie the polarization of cytoskeletal signaling proteins and membrane receptors in leader bleb migration. Leader

blebs are large pressure driven protrusions that many cells form in response to confinement and are thought to be an integrin independent mode of migration that cancer and other cells can utilize to navigate through low-adhesive confined environments. Previous work from this lab established that Erk signaling plays a prominent role in facilitating leader bleb migration and thus this current works set out to determine whether downstream signaling components displayed specific polarized distributions along the leader bleb, and whether these distributions were crucial for leader-bleb migration. They find that, surprisingly, the traditional (i.e. in mesenchymal migration) polarization of Rac and Rho are flipped in leader blebs, with Rac localizing at the base of the bleb and Rho localizing to the leading edge. Using biosensors and fluorescent constructs they then show that this polarization is established through the restricted mobility of EGFR by CD44 and ERM proteins, which concentrates Rac locally at the base of the bleb.

Overall this is a compelling series of observations that shows that EGFR/CD44/ERM/RAC/PIP3 are all spatially polarized with increased concentrations at the base of the leader bleb, and that PIP2/RhoA are concentrated at the leading edge of the bleb. Activation of EGFR, or its downstream effector Rac, at the bleb leading edge results in retraction of the leader bleb. Similarly, knockdown of CD44 or ERM proteins results in a loss of this gradient and results in reduced leader bleb formation. This seems like a reasonable model and the authors have done a good job breaking it down by components. There are a few comments/questions, however, about the data/interpretation:

One of the most surprising results of all these perturbations that disrupt this polarity, is that they have minimal effect on the persistence (Fig. 1K, 7E) or migration speed (EF. 1J, 5B) of leader bleb cells. How does one reconcile this with the argument that this polarity is crucial for leader bleb migration? Is it just the number of leader bleb cells that is impacted?

We understand and appreciate the reviewer’s concern. Based on the fact that several reviewers had similar concerns, we now realize that we did not clearly convey in our manuscript which graphs represented motility analyses only on the fraction pf population that exhibited leader bleb morphology versus all the morphologies. Indeed, while perturbations that disrupt polarity (EGFR/CD44 depletion or mislocalization of signaling) do not dramatically alter the persistence (Figure 1I, 1O) or average speed (Figure 1K, 1Q) of the subset of cells that successfully form leader blebs, they lead to a significant reduction in the *number* of cells able to actually form a leader bleb and maintain this migratory mode (Figure 1G, 1M). This decrease in the leader bleb–migrating population is reflected in the mean squared displacement (MSD) analyses of the **total cell population**, which show a striking loss in overall migration efficiency compared to controls (Figure 1H, 1N). **However to clarify we have now added cumulative frequency plots of speed of the entire cell population along with MSD of the whole population for all of our cell migration experiments (Figure 1J and 1P)**. We have revised the manuscript text and figure legends to clearly and concisely convey when analyses were performed on the whole population versus only on the fraction of cells in the leader bleb state.

Moreover, previous work has also shown that changes in the mean cell speed of a cell type does not typically reflect a global shift in the distribution but rather results from broadening of the distribution due to a subpopulation of faster-moving cells (Maiuri et al. 2012). These analyses show that while average speeds of cells that stabilize leader blebs are not significantly altered, the proportion of highly persistent leader bleb–driven cells is reduced upon EGFR or CD44 perturbation. Together, these observations reconcile the apparent discrepancy: polarity is indeed crucial for leader bleb migration, but its disruption primarily decreases the fraction of cells capable of entering this mode, rather than changing the intrinsic migration properties of those that do.

The persistence and speed plots referenced by the reviewer display only the trajectories of cells that successfully established leader blebs. Within this subpopulation, cells migrate efficiently, consistent with previous reports that once leader blebs form, they provide a robust mechanism for persistent migration, independent of the condition. However, when polarity is disrupted, fewer cells can access this highly efficient state. Thus, the major phenotype is a population-level reduction in leader bleb–driven migration rather than a strong effect on the performance of individual leader bleb cells.

We have modified the text in several places in the Results section to clarify this point:

"Analysis of cell motility showed that serum starvation strongly reduced migratory range for all cells, and the few cells that exhibited leader bleb morphology had reduced migration persistence but no difference in their speed while moving compared to leader bleb cells in serum (Extended Figure 1H-1K)."

and*:*

"Importantly, addition of EGF to serum-starved cells partially rescued the leader bleb morphology (Extended Figure 1F, 1G; Movie 2), increased the migratory range of all cells, and for leader bleb cells, increased the migration persistence, but had no significant effect on their speed (Extended Figure 1H-1K). Furthermore, targeting either EGFR tyrosine kinase activity with erlotinib (50nM), gefitinib (1μM), lapatinib (1μM) or blocking its downstream effector phosphatidylinositol 3-kinase (Pi3K) with wortmannin (100nM, Extended Figure 1M), strongly reduced leader bleb formation, cell migratory range and speed of the cells (Figure 1F-H,1J; Movie 3). The few cells that did make leader blebs under erlotinib or wortmannin treatment showed significantly reduced migration persistence, but no difference in migration speed compared to leader bleb cells in DMSO control (Figure 1I and 1K). Similarly, knocking down EGFR (EGFR-KD) by two independent siRNAs (Extended Figure 1N) strongly inhibited the formation of leader blebs, cell migratory range and speed compared to non-targeting siRNAs (NT), (Figure 1L-M, 1P; Movie 4J). Due to a drastic reduction in the number of cells exhibiting leader bleb morphology, especially in cells treated with siRNA2, EGFR-KD reduced both migration persistence and speed (Figure 1O, 1Q). Thus, EGF signaling through PI3K is required for stabilizing and maintaining polarized, elongated stable leader bleb morphology to drive persistent migration and promote migratory range under low-adhesive confinement. Furthermore, these results show that once cells establish and maintain a leader bleb, they migrate persistently and rapidly, independent of the perturbation, with the primary effect of perturbations being to inhibit formation and maintenance of leader blebs."

and:

"Analysis of cell motility parameters showed that siRNAs targeting the 3'UTR of CD44 (CD44-3'KD, Extended Figure 4J) significantly inhibited leader bleb formation (Figure 7C and 7D; Movie 14) and drastically reduced the migratory range of cells in low-adhesive confinement (Figure 7E). However, cells that managed to undergo leader bleb-based migration exhibited persistent migration with similar velocity (Figure 7F, 7H; Movie 15)."

With the migration analysis, the speeds are plotted as MSD, while the persistence is found by fitting the Furth equation. From the methods it's unclear how the data is being fit, or what the quality of this fit is. Typically, when calculating the MSD with overlapping time windows, you can only trust a small percentage of the actual MSD. The citation provided ([84]) mentions that normally only the lower 10% of the low-end time intervals are used for determining the slope. Given the parameters here (1 min intervals for 60 min) that would be a deltaT of only 6 min. That reference also explicitly states "DiPer does not calculate persistence time and other parameters that stem from Furth's formula" and includes a healthy discussion of the limitations of defining persistence in this way. What part of the MSD data was used to fit the equation? How good were the fits (this is never shown anywhere)? And is the persistence from the Furth equation a

good measurement for this type of migration? Finally, it's unclear from the plots whether the MSD is calculated for all the cells or just the cells with leader blebs.

We thank the reviewer for this important point and the opportunity to clarify.

Use of DiPer and MSD fitting: We used DiPer exclusively to calculate the mean squared displacement (MSD) with overlapping time windows, which provides a robust averaging method since the number of displacement vectors decreases with increasing time interval (Gorelik and Gautreau 2014). Importantly, we **did not use the MSD slope from DiPer to calculate persistence**. Instead, **we fit the MSD curves to the Fürth equation for a persistent random walk to extract persistence time and the diffusion coefficient.**

Fitting quality: As suggested, we now provide representative examples of both a good and a poor or no fit of Furth equation on the MSD in a Methods and Supplementary Figure 1 and here for the reference. Fittings were performed in Origin (Pro) using the non-linear curve function Levenberg Marquardt. Only fits with R (square) closer to 1 was accepted.

A) Good fit example

Model	Furtz
Equation	4 * D * (x - P * (1 - exp(-x / P)))
Plot	MSD
Diffusion coefficient	141.43458 ± 10.21155
Persistence	15.13612 ± 1.65215
Reduced Chi-Sqr	7503.06311
R-Square (COD)	0.99823
Adj. R-Square	0.99815

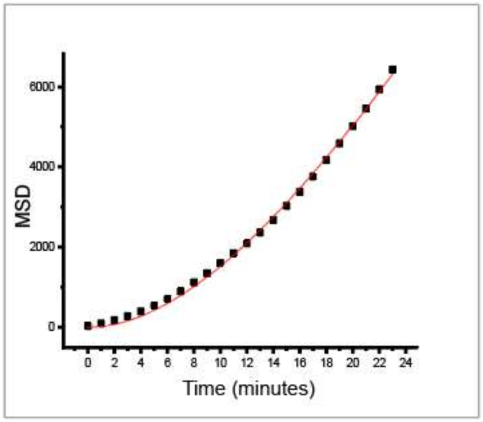

B) Poor fit example

Model	Furtz
Equation	4 * D * (x - P * (1 - exp(-x / P)))
Plot	MSD
Diffusion coefficient	1.00767 ± 3.66229
Persistence	2.88163E−8 ± 49.83256
Reduced Chi-Sqr	217174.83362
R-Square (COD)	−3.95717
Adj. R-Square	−4.1825

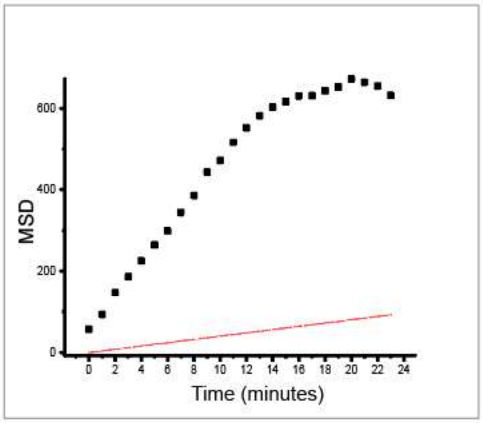

Time intervals and fitting range: While previous work (Gorelik and Gautreau 2014) cautions against using high time lags for slope-based persistence estimates, **our approach does not rely on slope extraction but instead on direct curve fitting.** We clarify this distinction in the Methods and supplementary text. It reads as

"MSD was computed for a given cell for step size n with N being the total number of displacements per trajectory and Δt the minimum time interval between the adjacent points in the trajectory. MSD for step size n was calculated for each trajectory and fitted to the persistent random walk (PRW) model using Furth's equation:

$$MSD = 4D[\Delta t - P\left(1 - exp\left(\frac{-\Delta t}{P}\right)\right)]$$

where D is the diffusion coefficient and P is the persistence time. Nonlinear fitting was performed in Origin (Pro) using the Levenberg–Marquardt algorithm, and only fits with $R^2 \approx 1$ were accepted. Persistence time and diffusion coefficients were plotted in GraphPad Prism."

Cell population included: We confirm that MSDs were calculated for the entire tracked population of cells, not only those forming leader blebs. Persistence values are plotted specifically for cells that exhibited leader bleb morphology (Figure 1H, 1I, 1N, 1O, 7E, 7F, Extended Figure 1H and 1I).

Discussion of limitations: We agree with the reviewer that defining persistence from the Fürth equation has inherent limitations. For migration under confinement, the MSD is visually very similar to what is expected from Persistent Random Walk where cells move persistently at short time intervals ($\langle MSD \rangle \sim t^2$) and more like random walk at longer time intervals ($\langle MSD \rangle \sim t$). This has now been shown in several seminal works (Stokes et al. 1991; Liu et al. 2015; Maiuri et al. 2015).

Together, these clarifications and additions strengthen the robustness of our analysis and make transparent the assumptions of using Fürth fits in this context.

The difference in kinetics of the optoEGFR and PA-Rac is striking. Why does the EGFR take 25 minutes to show a difference, while the Rac has an effect within just a few minutes? This is confusing too given that the FRAP experiments in Fig. 6A demonstrate that turnover is quite rapid throughout the bleb

We thank the reviewer for this insightful point. The most likely explanation for the difference in kinetics between opto-EGFR and PA-Rac reflects the distinct mechanisms by which these optogenetic constructs activate signaling. PA-Rac is a direct cytosolic effector: light stimulation immediately recruits and activates Rac at the membrane, leading to downstream signaling within minutes. In contrast, Opto-EGFR construct relies on light-induced clustering of cytoplasmic EGFR domains at the membrane (FusN-CRY2, OptoDroplet system discussed in details in the work (Farahani et al. 2021)), which is necessary for autophosphorylation and activation. The original work where Opto-EGFR was developed showed that Erk activation, that is downstream of EGFR signaling, reached peak around 30 min, explaining the ~25-minute timescale for observable effects (Farahani et al. 2021). It is to note that this does not mean activation of EGFR-PI3K-Rac pathway takes this long, except the opto-genetics constructs we are using have differences in the operating mechanism.

In summary, the slower response of opto-EGFR compared to PA-Rac is expected given the mechanistic differences and differences in the constructs: rapid cytosolic activation versus light-induced receptor clustering and autophosphorylation.

In the polarization measurements of EGFR, there is a clear peak about 25% of the way up the bleb (Fig. 2B, 3A, and seen most easily in Movie 12 on the left). Why does the EGFR concentrate in this area, while Rac/PIP3/CD44 peak at the base of the bleb? Also, comparing the polarization plots of EGFR-GFP (Figs. 2C, 3C) the distributions don't seem to match very well. What's the difference in conditions between these two plots?

We thank the reviewer for their keen eyes. We also have noted this peak of EGFR about a quarter of the way up the bleb compared to Rac, PIP3, and CD44 which exhibit distributions closer to an exponential decay

from the bleb base towards the tip. Examination of the dynamics of EGFR distribution during leader bleb formation shows that this peak forms as soon as the bleb is fully extended and is maintained. Our modeling explains the initial increase of density from the base forward; i.e., the observation that there is a peak. The explanation offered by the model is anterograde flow of bulk membrane.

However, we do suspect that there could be a very small contribution from actin retrograde flow at the back of the bleb. Since actin is not in close proximity to the membrane in the distal half of the bleb where flow is fast, it cannot act to sweep EGFR back. But right where the peak in EGFR density is, this is also approximately where actin is switching from a bundled organization to an isotropic network, and coming in close proximity to the membrane, and the retrograde flow, although much slower that in more distal regions of the bleb, is still active. Thus, we think that in this zone membrane proximal actin (Extended Figure 3C and 3D) can sweep the EGFR back a bit (though poorly, since EGFR does not bind to actin directly) and cause it to pile up in this region. In the bleb base, actin is in an isotropic mesh close to the membrane and retrograde flow is negligible, and here the high levels of CD44 simply trap the EGFR, but since there's no actin flow, it is not swept. Since the positions of the change in actin architecture from bundled to mesh, membrane distal to membrane proximal, and fast to slow flow rate and the position of EGFR "pileup" are slightly variable from cell to cell, it really is impossible to test this prediction experimentally.

CD44 has a stronger interaction with actin with ERM proteins and hence is trapped itself effectively at the base of the bleb as the bleb protrudes, more efficiently compared to EGFR (compare Figure 6G to Figure 5C). With our current scope of the work we do not know how does active Rac and PIP3 peak at the base of the bleb. Our hypothesis is that this could possibly be because of local activation of PIP3/Rac and the on/off rates of proteins on the inner leaflet of the plasma membrane.

Many of the blebs in these movies seem quite stable and the cell isn't moving much. Is there a correlation between the magnitude of the gradient and the speed of the migration? Does this suggest that bleb polarization is in part decoupled from force transmission?

We thank the reviewer for this insightful comment. Our data include movies captured at two different timescales. Movies depicting migration are 60 min long with 1 min intervals and captured with a low mag, low NA lens to show cells actively moving, whereas movies used for EGFR and actin dynamics, as well as optogenetics, biosensor, and FRAP experiments are taken with high mag, high NA lenses and are <1 min long, with images taken at time intervals on the seconds scale to show dynamics of proteins and their activities, during which blebs remain relatively stationary. Because of these very different length- and time-scales, it is impossible to correlate gradient steepness to cell migration speed on a cell-by-cell basis.

Across our datasets, we observe that cells with leader blebs, which migrate more persistently, exhibit more strongly polarized EGFR (as measured by the polarity index, Figure 3A and 3B) compared to cells with dynamic elongated blebs. However between dynamic and leader blebs, we do not see any significant differences in the instantaneous speed (Extended Figure 1 D and E). This suggests that while polarization and force transmission are related, bleb signaling polarization can occur independently of immediate net movement, indicating a partial decoupling between signaling organization and traction generation.

Since CD44 is helping to stabilize the leader blebs, does overexpression of CD44 result in more stable blebs? And do those cells move faster?

Overexpression of CD44 does not result in more stable blebs. We have now added this to our analysis in (Figure 7C and 7D) where the bleb distribution does not change compared to the controls. In addition, over expression of CD44 decreased the efficiency of migration as measured by average MSD of all the cells

moving under confinement (Figure 7E) and we find that cells actually move slightly slower. We have added a description of this new result to the text as follows:

"Notably, reintroducing CD44-GFP into CD44-3'KD cells rescued the percentage of cells undergoing leader bleb-based migration (Figure 7D) and restored the migratory range to that of NT controls (Figure 7E), while overexpression of CD44 in non-targeting controls had no effect on leader bleb formation."

How does CD44 sequester EGFR when the EGFR seems to go further into the bleb during formation than the CD44 (Fig. 5C vs Fig. 6J)? What's causing the initial EGFR retraction towards the bleb base? They only really seem to overlap starting at the 8 min mark.

Thanks to the reviewer for this thoughtful comment. As noted above, every cell, and every bleb is different, with the peak of EGFR in a slightly different place, different locations for the transitions in actin speed, membrane proximity and architecture, with slight differences in the steepness of the CD44 gradient, etc. For our figures showing EGFR or CD44 dynamics during the process of bleb formation, it should be noted that given the timescale of imaging fluorescent protein dynamics, the photobleaching of the probes, and the time required for bleb formation, catching the event of leader bleb formation was a rare and fortuitous event. Thus, we do not have enough data on these events to quantify them for average EGFR or CD44 distribution as a function of time during bleb formation.

While we can draw some qualitative conclusions from a few examples, conclusions about the timing of the EGFR and CD44 gradients relative to each other during bleb formation cannot be drawn from the data we were able to obtain. Based on the available data we believe that as the bleb protrudes, EGFR and CD44 moves forward (anterograde movement and lateral diffusion, supported by modelling and FRAP experiments), this is at the same time while the membrane proximal actin is establishing. As the bleb protrudes and becomes more stable, the strength of CD44 and actin interaction also increases corralling EGFR more efficiently. To understand this correlation more, we did try to co-image EGFR-RFP and CD44-GFP in transient transfections, however EGFR-RFP constructs were not efficiently localizing to the plasma membrane, making these experiments difficult.

I understand the basic argument that the transmembrane interaction of CD44/EGFR is helping to activate PIP3/Rac and sequester it locally, but what is driving the localization of Rho to the tip of the bleb? Is it merely a response to local antagonism from Rac activation? Or is it also an actively maintained population?

We thank the reviewer for this insightful question and is currently one of the major questions in our follow up work. While local Rac activation at the bleb base contributes to spatial segregation by inhibiting Rho in that region, Rho accumulation at the tip could also be actively maintained.

Mechanistically, Rho localization likely involves multiple coordinated inputs:

Local lipid environment: Bleb tips are enriched in PIP2, which provides a favorable environment for maintaining active RhoA (Budnar et al. 2019).

Spatial inhibition from Rac/PIP3: Rac at the bleb base helps restrict Rho to the tip by local antagonism, sharpening the gradient (De Belly et al. 2024).

Delivery of specific Rho-GEFs: Bleb tips might be enriched with active Rho by delivery or trapping of specific RhoGEFs to the tip the bleb with the help of microtubules that run all the way to the tip of the bleb (Adams et al. 2021).

However, the precise upstream signals responsible for activating Rho at the bleb tip remain unknown and represent an interesting avenue for future investigation.

Lastly, while this is a reproducible phenomenon in vitro where low-adhesion coupled with confinement produces these leader blebs, I struggle with where the cell might encounter such an environment physiologically. While certainly confinement is rampant in physiological systems, it is almost always in the presence of extracellular matrix proteins, or other cells where ligands are abundant (like in Fig. 5D). It would be useful to comment on this seeming contradiction.

We thank the reviewer for this thoughtful comment and fully appreciate the concern regarding the physiological relevance of confinement-induced blebbing in the presence of extracellular matrix (ECM) proteins. We agree that in vivo confinement is rarely experienced in the complete absence of matrix- or cell-associated adhesive ligands. Indeed, high confinement is abundant in physiological settings such as tumor microenvironments and interstitial tissues, as shown by intravital imaging (Weigelin et al. 2012).

Importantly, whether cells form blebs or lamellipodia under confinement is strongly influenced by their contractility state. Highly contractile cells have a greater tendency to undergo bleb-based migration (Lomakin et al. 2020; Venturini et al. 2020). Under extreme confinement, deformation of the nucleus stretches the nuclear envelope, activating stretch-sensitive proteins and the enzymatic activity of cPLA2 at the nuclear membrane. This results in increased myosin II recruitment, enhancing intracellular pressure and favoring bleb formation. Thus, in both 2D and 3D adhesive systems or matrices, confinement can bias cells toward blebbing morphologies through a mechano-metabolic mechanism.

Consistent with this, prior work has shown that when confined cells forming stable blebs are plated on fibronectin, they assemble small focal adhesions but maintain a blebbing morphology (Ruprecht et al. 2015). In line with these reports, our own data demonstrate that metastatic melanoma cells form leader blebs not only in low-adhesive confinement but also in 3D collagen matrices, where CD44 and EGFR maintain the same rear-to-front polarized gradients (Fig. 5N; Jha et al., 2025). This confirms that the molecular principles we describe operate in more physiologically relevant, ligand-rich confined environments.

Therefore, while our study utilizes a low-adhesive confinement assay to dissect the fundamental molecular mechanisms of leader bleb polarity, the phenomenon is highly relevant to in vivo settings where cells encounter both high physical confinement and ECM ligands. Rather than a contradiction, we view this as evidence that confinement and contractility can override adhesive signaling to favor bleb-based motility in dense tissues.

Minor points:

- The modeling component was a bit unclear. Why are two different epsilon variables needed? This wasn't explained well in the main text and seemed mostly just to be fitting the experimental data (which was much more convincing).

We thank the reviewer for mentioning this for clarification, we have mentioned this in the methods section these two epsilon parameters account for two, distinct corralling effects. For one, transmembrane proteins do not flow as easily as the bulk membrane flow from base to tip of the bleb; the second epsilon parameter allows for the (retrograde) contribution associated with F-actin interaction. Thus, the model allowed for two contributions, which we subsequently tested experimentally. It currently reads as-

"..If the bleb has a constant shape, it implies that there is zero flow at the tip ($V_m(L) = 0$). This boundary condition dictates that $V_m(x) \geq 0$; membrane flow is towards the tip of the bleb. For the calculations presented here, we took $k_m(x)$ = constant and set its value to 0.003 s^{-1}, thus approximately matching the estimated bulk flow velocity of $V_m \approx 0.1$ $\mu m/s$ measured at $x \approx 10$ μm. Relative to the calculated $V_m(x)$, we

consider that $V_p(x)$ is potentially influenced by two distinct, protein-corralling effects: restriction of free protein mobility, and physical interaction with the F-actin retrograde flow. Defining $V_F(x)$ as the local velocity of the F-actin, we take

$$V_p(x) = (1 - \varepsilon_1)(1 - \varepsilon_2)V_m + \varepsilon_2 V_F,$$

where $\varepsilon_1(x)$ are $\varepsilon_2(x)$ are variables, each ranging between 0 and 1, which we refer to as corralling efficiencies. Their values reflect the 'strengths' of protein mobility restriction and interaction with flowing F-actin, respectively."

- The data presentation isn't always consistent. Sometimes individual data points are shown on the violin plots (e.g. Fig. 2C) and not on others (Fig. 2G,I,J) even though there are no significant differences in the number of cells compared.

We thank the reviewer to mention this, we have now unified the violin plots with individual data points shown.

- In Fig 7E the boxplot have 3 points on them, but the legend is unclear what they represent. Are these the average values of the biological repeats? Do the boxplots represent the combined data? And were statistics performed on the combined individual data points, or the means of the biological repeats?

We apologize to the reviewer for the lack of clarity on our part we now consistently have persistence of cell exhibiting leader bleb morphology (only the ones with successful Furth fits) box plots show individual data points combined from multiple independent experiments. (n=number of cells and N= number of independent experiments). In genetic and drug perturbations, often we saw very few cells exhibit leader bleb morphology and even lower number with Furtj fit to calculate persistence. Hence, the statistics were performed on the combined individual data points, which is now mentioned in the Methods section. We have now provided all the analysis and statistical measurements done in Source data files.

- The circle in the PA-Rac is given significantly different dimensions throughout the manuscript. In the text (line 303) is says 5 um diameter, Figure 3F says 20 um, movie 5 shows 10 um diameter

Thank you for pointing out this error, we have now rectified this.

- Movie 8 has the actin channel repeat twice before switching to show a single loop of the EGFR?

We have now fixed this error.

- It's notoriously hard to make glass truly non-adhesive even at high concentrations of PLL-PEG (PMID: 38781207). Low-adhesive might be a better term.

We thank the reviewer for this input, we have indeed now switched the term to low-adhesive throughout the manuscript.

Reviewer #3 (Remarks to the Author):

Jha et al

CD44 and Ezrin restrict EGF receptor mobility to generate a novel spatial arrangement of cytoskeletal

signaling modules driving bleb-based migration

In this manuscript Jha et al set out to identify mechanisms mediating polarized bleb-based migration of metastatic melanoma cells in a confined, non-adhesive environment. Increasing evidence suggests that some tumors, especially melanoma, invoke bleb-based motility as opposed to the much better-studied mesenchymal mode of invasion, so the more we understand how it works the better. Using a sophisticated toolkit of super-resolution confocal imaging, biosensors, and optogenetic experiments together with a careful cell biological analysis of bleb formation in melanoma cells they identified a rear-to-front EGFR gradient that polarizes PI3K-Rac signaling through a mechanism involving the restriction of EGFR mobility at least in part, through CD44 and ezrin.

This well-written experimentally solid manuscript addresses an important cell biological aspect of tumor behavior. The authors missed some opportunities to embrace the complexity of this seemingly simple process and acknowledge necessary limitations of their approach. With some key additional experiments and discussion, it should make an important publication in Nature Cell Biology.

Major points:

Specificity: After a nice morphogenetic analysis of bleb formation in these cells, the authors jump to EGFR as a mechanism largely because they contrast their findings with studies of mesenchymal migration. While they present evidence that EGFR is polarized in leader blebs and contributes to their formation, the effects are somewhat modest, and the phenotype of erlotinib treatment appears to be different from wortmannin treatment. Moreover, the focus on EGFR masks the important question of whether the biology of bleb polarization is specifically driven by one or a subset of receptors and corralling mechanisms. The authors should test the involvement of another receptor (ie FGFR) and another potential ERM-picket (ie ICAM) for comparison to address this important question. It is interesting either way.

We thank the reviewer for this important comment. We agree that the biology of leader bleb polarization is unlikely to be driven by a single receptor alone, and that corralling mechanisms may extend beyond EGFR/CD44/ERM.

Magnitude of EGFR perturbations: The difference in persistence of cells undergoing leader bleb-based migration between EGFR knockdown and siRNA controls is not surprising because once cells manage to form a stable leader bleb, they migrate efficiently, independent of the condition or treatment (Figure 1O). The main difference between these conditions that we see is the number of cells that manage to form and stabilize a leader bleb. As noted above, our communication was very unclear about which analyses were performed on the total cell population versus only the population of cells that actually did form leader blebs. We have now clarified this in the writing and in how the figures are labeled. Mean square displacement was measured and graphed for all the cells (exhibiting all different morphologies), however the persistence was only calculated for cells that exhibited leader bleb morphology. We have refined our conclusion on this point as follows:

However to reinforce the role of EGFR in the maintenance of leader blebs, in the revised manuscript we have now included additional perturbations targeting EGFR. Specifically, we tested a second independent EGFR siRNA probe that targets a distinct sequence of EGFR as well as two additional, clinically relevant small-molecule inhibitors, **Lapatinib** (dual EGFR/HER2) and **Gefitinib** (EGFR-specific). Our new results show that all of these perturbations showed strong phenotypes, with a significant decrease in the fraction of cells that form stabilized leader blebs and a dramatic increase in the small, transient bleb population (Figure 1G, Figure 1M). Taken together, these results strengthen our conclusion that EGFR is one of the primary receptors required for leader bleb-based migration. We revised the text as follows:

"…addition of EGF to serum-starved cells partially rescued the leader bleb morphology (Extended Figure 1F, 1G; Movie 2), increased the migratory range of all cells, and for leader bleb cells, increased the migration persistence, but had no significant effect on their speed (Extended Figure 1H-1K). Furthermore, targeting either EGFR tyrosine kinase activity with erlotinib (50nM), gefitinib (1µM), lapatinib (1µM) or blocking its downstream effector phosphatidylinositol 3-kinase (Pi3K) with wortmannin (100nM, Extended Figure 1M), strongly reduced leader bleb formation, cell migratory range and speed of the cells (Figure 1F-H,1J; Movie 3). The few cells that did make leader blebs under erlotinib or wortmannin treatment showed significantly reduced migration persistence, but no difference in migration speed compared to leader bleb cells in DMSO control (Figure 1I and 1K). Similarly, knocking down EGFR (EGFR-KD) by two independent siRNAs (Extended Figure 1N) strongly inhibited the formation of leader blebs, cell migratory range and speed compared to non-targeting siRNAs (NT), (Figure 1L-M, 1P; Movie 4J). Due to a drastic reduction in the number of cells exhibiting leader bleb morphology, especially in cells treated with siRNA2, EGFR-KD reduced both migration persistence and speed (Figure 1O, 1Q). Thus, EGF signaling through PI3K is required for stabilizing and maintaining polarized, elongated stable leader bleb morphology to drive persistent migration and promote migratory range under low-adhesive confinement. Furthermore, these results show that once cells establish and maintain a leader bleb, they migrate persistently and rapidly, independent of the perturbation, with the primary effect of perturbations being to inhibit formation and maintenance of leader blebs."

At the same time, we acknowledge the reviewer's point that EGFR may not be the only RTK relevant in this context. EGFR (HER1) is one of several RTKs that converge on the MAPK pathway; others include HER2, FGFR, and c-Met (Gerosa et al. 2020). It is therefore highly likely that additional receptors could influence leader bleb polarity through overlapping signaling pathways. While a systematic comparison of multiple receptors is beyond the scope of the present study, we now explicitly acknowledge in the text that other RTKs may contribute to the signaling architecture we describe, and we have softened the emphasis on EGFR as the *sole* receptor by clarifying the language in the Results and Discussion which reads as follows:

"Other RTKs such as HER2, FGFRs, c-Met that like EGFR also converge on PI3K-MAPK [62] may similarly engage in this corralling-based mechanism to contribute to polarity during bleb-based migration"

Thus, our data firmly establish EGFR as a critical driver of leader bleb polarization while leaving open the important possibility that other RTKs and corralling mechanisms (such as ICAM or other large transmembrane proteins) also play roles in regulating bleb-based migration in confined environments.

EGFR activity vs clustering: The evidence that EGFR activity is required as opposed to clustering/aggregation is modest. In Fig 3 the use of the optical sensor is a nice tool but should be done with and without EGF and in the presence of erlotinib in order to draw the conclusion that EGFR signaling versus just clustering is important. Additionally, are the distributions of the EGFR, AKT and PAK sensors blocked by erlotinib?

We thank the reviewer for raising this important point about distinguishing EGFR activity from clustering. EGFR activation indeed involves ligand binding (e.g., EGF) that drives conformational changes, receptor dimerization/clustering, and subsequent auto-phosphorylation (Kim et al. 2021). Thus, clustering and signaling are inherently linked. In our study, we examined EGFR activity using a phosphorylation-sensitive optical sensor (EGFR-CD3 + ZtSH2), which directly reports on receptor activation. Importantly, these measurements were performed in the presence of serum, which contains EGF and other growth factors, and we observed robust rear-to-front gradients of both total and active EGFR in leader blebs (Fig. 2F–2I).

Consistent with this, stimulation of serum-starved cells with exogenous EGF increases the fraction of cells exhibiting stable leader bleb morphology by ~50% compared to starved cells, confirming the requirement for ligand-induced receptor activation (Extended Fig. 1F, 1G). Conversely, blocking EGFR phosphorylation using erlotinib, lapatinib and gefitinib or inhibiting its downstream effector PI3K with wortmannin significantly

reduces the number of cells with stable leader blebs and efficiency of migration (Figure 1G, 1H, 1M and 1N). These results indicate that EGFR signaling activity is critical for leader bleb stabilization and efficient bleb-based migration.

To address the reviewer's concern about whether EGF activity is required for the formation of the EGFR gradient in leader blebs, we have now analyzed EGFR distribution in confined cells in the presence of erlotinib compared to the DMSO controls. We find that inhibition of EGFR phosphorylation alters the distribution of EGFR-GFP, flattening the rear-to-front gradient and significantly reducing the polarity index (Figure 2D and 2E). This supports the conclusion that EGFR phosphorylation activity contributes to the polarized distribution of the receptor. We have modified the text as follows:

"We found that EGFR activity itself was required for the rear-to-front EGFR gradient in leader blebs, as erlotinib treatment showed that inhibition of EGFR phosphorylation caused flattening of the EGFR-GFP rear-to-front gradient and significantly skewed the polarity index towards zero compared to DMSO control (Figure 2D, 2E)."

Together, these data demonstrate that EGFR activity is required for leader bleb polarization, while clustering provides the structural basis, phosphorylation-driven signaling is essential to establish and maintain the polarized distributions of EGFR that stabilize leader blebs and promote persistent migration.

MAPK signaling: These melanoma cells harbor an activating BRAF mutation and this group has previously argued that ERK is required for bleb-based migration (Logue et al. 2015). The authors should discuss their results in the context of these previous results. In the introduction it is pointed out that this signaling is linked to EGFR/Ras/Raf pathways, but it is not addressed further.

We thank the reviewer for bringing this up. We have now included it in the intro and discussion.

Introduction:

"In this study, we aimed to identify the mechanisms that enable the persistent cell polarity that drives bleb-based migration of metastatic cancer cells in confined, low adhesive environments. We previously showed that leader bleb-based migration in metastatic melanoma cells under confinement is driven by Erk signaling[19], which is linked to the EGF/EGFR/Ras/Raf pathways. We thus hypothesized that the polarized distribution of growth factor RTKs and their downstream signaling components, such as phosphoinositides and Rho GTPases[37–39], are required for maintaining polarity and driving persistent migration in metastatic melanoma cells under confinement."

Discussion:

"The spontaneous polarization of metastatic melanoma cells in the absence of chemical or haptotactic gradients, coupled with their efficient migration in confined, low-adhesion environments, has important implications for tumor invasion and metastasis[5,69,74]. Previous studies have shown high Erk signaling in melanoma cell blebs, likely due to localized, restricted signaling[19]."

Requirement for the leader bleb creation versus polarization – can these be segregated? The optogenetic experiments work to make this distinction. For each perturbation (ie interfering with EGFR, CD44, ERMs), but the authors should show how the distribution of bleb phenotypes is altered (ie purple bar graphs) and relate it to the knockdown efficiency (which is not shown). It may be hard to fully segregate but this should be addressed up front and discussed.

We thank the reviewer for raising this important point. We agree that distinguishing the requirements for *leader bleb creation* versus *polarization/maintenance* is critical, and that this distinction was not sufficiently emphasized in our initial presentation.

<u>Creation versus polarization</u>: Our optogenetic experiments were designed specifically to test **maintenance** of bleb polarization rather than bleb **initiation**. By locally clustering Opto-EGFR or activating PA-Rac at the tip of *pre-existing* leader blebs, we acutely disrupted their rear-to-front signaling gradients. This caused bleb shortening, loss of actin cytoskeletal bundling at the leader bleb tip, and ultimately caused bleb retraction (Fig. 3D–H), demonstrating that EGFR/Rac gradients are required for *stability and polarity maintenance* rather than de novo creation of a leader bleb. We will now clarify this distinction explicitly in the our conclusion as follows:

“Together, these results demonstrate that the rear-to-front gradients of EGFR and Rac1 activities are required for bleb stability and maintenance to drive persistent migration in low-adhesive confinement.”

<u>Bleb phenotype distributions and knock down efficiency (“purple bar graphs”)</u>: For each perturbation, we score cell morphologies in the following categories: 1) transient blebs, 2) multiple elongated blebs, 3) dynamic elongated blebs, or 4) stable leader blebs (Fig. 1B; 1G, 1M, Fig. 7C–E). To meet the reviewer’s request, we have now included western blots for the EGFR knockdowns and drug treatments (Extended figure 1L, 1M and 1O). However bleb morphologies, persistence, and persistence are such a combinatorial effect of corralling, signaling via receptors and contractility, we do not expect to have a direct correlation between knockdowns and these parameters.

We agree with the reviewer that full segregation of creation vs. polarization may be challenging, but our optogenetic experiments strongly support a requirement for signaling gradients in *polarization/maintenance*, while the knockdown/perturbation analyses address the broader role of EGFR, CD44, and ERMs in both initiation and stability.

How do you harmonize your observations with the established dynamic role of ERM proteins bound to PIP2 in bleb retraction? Indeed, Ezrin looks to be also enriched at the bleb tip in Fig 5D exactly as you would expect. The authors should at least discuss how the state of multiple small blebs, that are perhaps retracted by PIP2-bound ERM proteins, transition to a single elongated bleb with ERMs also concentrated at the base. Do ERM proteins also localize similarly in 5G?

We thank the reviewer for bringing this point out. Ezrin (ERM proteins) is a cytosolic protein that has a FERM domain to bind the plasma membrane, prefers PIP2 and binds to the cytoskeleton in a phosphorylation-dependent manner (Ehret et al. 2023). To address the localization disparity the reviewer mentioned, we now show immunostaining of both total Ezrin and phosphorylated ERM proteins in the leader bleb. Even though we see total ezrin in the rear and the tip of the leader bleb (Figure 5J, 5K), **phosphorylated ERM protein is absent from the bleb tip and exclusively concentrated toward the bleb base** (Figure 5L and 5M), where it can bind to both membrane and the cytoskeleton to not only corral EGFR with CD44 but also prevent active retraction of the leader blebs.

Finally, to address the transition the reviewer highlights from “multiple small blebs” (where PIP2-ERM–driven retraction dominates) to a “single elongated leader bleb” with phosphorylated ERMs concentrated (p-ERM) at the bleb base, we now propose and discuss it upfront here that (1) confinement-induced pressure seeds many small blebs; these smaller blebs are retracted back quickly because of ERM localization (Aoki et al. 2016) (2) once a single bleb begins to dominate, actin coherence decreases and membrane-proximal actin increases at the rear, recruiting CD44/ phosphorylated ERMs to form corrals; (3) these corrals restrict EGFR mobility, establishing a rear-to-front EGFR/PI3K/Rac1 activity gradient that stabilizes the elongated

morphology. We apologize to be not able to include this in discussion due to space constraints. However we have modified the text of Result sections as follows:

“Immunostaining for CD44, ezrin, or active, phosphorylated ERM proteins (phospho-ERM) in confined cells revealed a concentration of CD44 in the cell body and both ezrin and phospho-ERM concentrated in the leader bleb. Line-scan analysis of leader blebs showed that while ezrin was localized throughout the bleb, CD44 and phospho-ERM formed rear-to-front gradients (Figure 5J-5M).”

Other Points:

Please clarify the concentration of drugs used. The values given in the text, figure legend and methods are different. If erlotinib is being used at 10uM that is extremely high and lower doses should be used but I think this is a typo.

We thank the reviewer for bringing out this error we have now mentioned the working concentration used in the main text and the stock concentration prepared in the Methods section.

Can you apply statistics to Fig 1G?

We have now added SD error bars for all the bleb distribution stacked column bars and are now new figures (Figure 1G, 1M, Extended Figure 1G, 7D).

Is 1J it statistically significant? the shRNA EGFR data is quite modest - do these cells express other ErbB family members?

EGFR siRNA knockdowns: We thank the reviewer for this point, We agree that the difference in between EGFR-KD and control siRNA was not striking in the initial dataset. However, to address our weak conclusion based on a single siRNA targeting a unique sequence EGFR, we repeated experiments with an independent siRNA targeting a different unique sequence in EGFR and a different control siRNA. These data confirm that EGFR-KD causes a significant reduction in leader bleb formation and a corresponding increase in the population of cells displaying only small transient blebs (Figure 1L, 1M), with also a drastic reduction in the efficiency of migration of all the cells (Figure 1N). We also now measured the speed of all the cells and saw a significant population of cells moving slowly, as demonstrated in cumulative frequency plots of cell speeds (Figure 1P), strengthening the conclusion that EGFR depletion disrupts leader bleb–based migration.

Other ErbB family members: We agree with the reviewer that EGFR (HER1) is indeed one of several receptor tyrosine kinases (RTKs) capable of influencing MAPK signaling, along with HER2, c-Met, and FGFR. Other ErbB members so indeed express in melanoma cells (Gerosa et al. 2020). To further address this concern, we tested two additional inhibitors: **Lapatinib (HER1/2 dual inhibitor) and Gefitinib (EGFR-specific inhibitor).** Like our results with erlotinib, both treatments resulted in a strong reduction in leader bleb formation and a dramatic increase in cells with small blebs (Figure 1F and 1G) and severely impacted the efficiency of migrating cells (Figure 1H).

Ext. Fig. 1F: Include color scale on temporal projection

We thank the reviewer for pointing this out, we have included the missing color scale on the temporal projection.

Ext. Fig. 1K: This doesn’t really tell you that EGFR signaling is normally maintaining pEGFR or pAkt in

these cells, but rather that they can be stimulated and seen at short timepoints. To argue that EGF is the 'serum factor responsible for promoting leader bleb-based migration…' would require replacing serum with EGF and measuring steady-state levels after >24h.

We thank the reviewer for pointing out this, all our experiments were performed after adding EGF (stimulation) and imaging performed within 2-3 hours of adding that. In that time range EGF stimulation of serum starved cells is enough to stabilize leader bleb morphology and hence we conclude that EGF is one of the serum factor responsible for promoting/maintaining leader bleb-based migration. Taken together with drug perturbations impacting phosphorylation of EGFR and Akt (Extended Figure 1L), we conclude that EGF/EGFR/PI3K pathway is one of the major growth factor receptor pathway required for maintaining stable bleb morphology.

Ext. Fig. 1N: Was this blot carried out under steady state conditions or with EGF stimulation? Results are inconsistent with steady state signaling from the blot in K.

Extended figure 1N in our previous submission was to show the efficiency of EGFR siRNA when treated for 24, 48 and 72 hours without any particular EGF stimulation (cells were in the media with serum). These blots are not directly comparable to the blot in K (serum starvation and stimulation with EGF) due to difference in the number of cells collected/ confluency of the dishes. We now have now also added a blot showing the reduction in the phosphorylation of EGFR (cells raised in media with serum) after Erlotinib, Lapatinib and Gefitinib drug perturbations showing indeed there is significant, EGFR phosphorylation that occurs in steady state conditions that is reduced with the drug treatments.

add back a kinase dead version of EGFR to confirm that EGFR activity

Since we could not make a EGFR KO because often that leads to very low cell count due to effects on cell proliferation (Orofiamma et al. 2022; Ludwig et al. 2023), this experiment would have been difficult or clean. To further address this concern, we tested two additional inhibitors: Lapatinib (HER1/2 dual inhibitor) and Gefitinib (EGFR-specific inhibitor). Like our results with erlotinib, both treatments resulted in a strong reduction in leader bleb formation and a dramatic increase in cells with small blebs (Figure 1F and 1G) and severely impacted the efficiency of migrating cells (Figure 1H).

It would be helpful to include EGF concentration in the figure legend.
We thank the reviewer to point out the error, we have now included that in the figure legend.

There is a typo in line 341
We thank the reviewer to point out the error, we have fixed the typo

Fig. 3: Why is the timescale so different for EGFR and Rac optogenetic experiments? In the Rac optogenetic experiment a 4 min. timepoint is used – at this point is it possible to distinguish between a leader and dynamic bleb? The retraction results seem quite modest. It is difficult to see lamellipodia protrusions in 3F – it would be helpful to show a zoomed inset.

We thank the reviewer for this insightful point. The most likely explanation for the difference in kinetics between opto-EGFR and PA-Rac reflects the distinct mechanisms by which these optogenetic constructs activate signaling. PA-Rac is a direct cytosolic effector: light stimulation immediately recruits and activates Rac at the membrane, leading to downstream signaling within minutes. In contrast, Opto-EGFR construct relies on light-induced clustering of cytoplasmic EGFR domains at the membrane (FusN-CRY2, OptoDroplet

system discussed in details in the work (Farahani et al. 2021)), which is necessary for autophosphorylation and activation. The original work where Opto-EGFR was developed showed that Erk activation, that is downstream of EGFR signaling, reached peak around 30 min, explaining the ~25-minute timescale for observable effects (Farahani et al. 2021). It is to note that this does not mean activation of EGFR-PI3K-Rac pathway takes this long, except the opto-genetics constructs we are using have differences in the operating mechanism. In summary, the slower response of opto-EGFR compared to PA-Rac is expected given the mechanistic differences and differences in the constructs: rapid cytosolic activation versus light-induced receptor clustering and autophosphorylation.

Regarding reviewers concern about Rac optogenetic experiments, 4 min is not enough to distinguish between a leader and a dynamic bleb. However, these experiments were done on the blebs that appeared stable within the experimental observation (~2-3 hours). In photo-activable Rac experiments, the retraction was relatively fast since the effect of activation of Rac with these constructs was quicker as mentioned above. The protrusions that we see are lamellipodia like protrusions however we have not resolved if to see the details of these protrusions since these are not very prominent presumably due to lack of integrin activation.

- Fig. 3: it is not clear that EGFR activity is required - this is exogenous

As mentioned before Opto-EGFR construct relies on light-induced clustering of cytoplasmic EGFR domains at the membrane (FusN-CRY2, OptoDroplet system discussed in details in the work (Farahani et al. 2021)), which is necessary for autophosphorylation and activation. This is independent of exogenous presence of EGF. Along with the experiments that show that drug perturbation to inhibit EGFR activity drastically reduce the cells exhibiting leader bleb morphology, we are convinced that EGFR activity is required for bleb stability.

Fig. 5G: is this endogenous EGFR? The addition of an additional model is nice, but it would be helpful to use it in additional contexts in the manuscript.

We thank the reviewer for this, indeed the EGFR the figure (now Figure 5N) shows immunostaining of endogenous CD44, EGFR. However, we limit this only to this since this requires development of advanced 3D image analysis quantification approaches, since simple line-scan measurements on a single optical plane as is done on cells under confinement in 2D where the plasma membrane is pressed against the coverslip, are not sufficient in 3D. We therefore believe this represents an important but more extensive line of investigation that will be better addressed in a future study dedicated to dissecting signaling gradients in bleb-based migration in 3D microenvironments and *in vivo*.

Fig. 6: Evidence of CD44, ezrin, and moesin knockdown should be shown.

We thank the reviewer for their insight and we have now added this in the manuscript (Extended Figure 4).

When the authors say: ‘Neither CD44 or ezrin/moesin knockdown impacted actin organization at the base of the bleb’ what do they mean? the images in Ext. Fig. 4E and F look quite different

We thank the reviewer in pointing this out. The actin organization closer to the neck of the bleb in both CD44 sirna and ezrin+moesin sirna compared to their NTsirna controls were not very different. However the reviewer is correct that the actin organization changes moving from the neck towards the tip. In CD44sirna, the actin remains more mesh like as measured by the actin coherency (Extended Figure 4F, 4G), while in contrast the actin is more loosely arranged in ezrin+moesin sirna (Extended Figure 4H and 4I). We have now corrected this in the manuscript and it reads as-

"Interestingly, imaging of FR-F-tractin showed that CD44-KD and EM-KD caused disparate effects on actin organization, with CD44-KD slightly decreasing and EM-KD increasing actin filament coherency (Extended Figure 4F- 4I)."

EGFR-GFP gradient is lost both in CD44-KD and EM-KD even with disparate effects on actin, in CD44-KD, the transmembrane picket is lost to corral EGFR, in the EM-KD, the link between the actin and the plasma membrane is lost which impacts the corralling of EGFR. Hence EGFR corralling and gradient requires both presence of picket protein an its link to the underlying actin cortex.

Are the phenotypes described here specific to this cell line, or generalizable to other melanoma cell lines or other metastatic tumor cells?

The phenotypes shown here are not just specific to this cell line, large stable blebs are formed by many cell types which have intrinsic high cell polarity (Liu et al. 2015; Ruprecht et al. 2015). We also tested if transmembrane protein gradients are seen in other cell types as well. We saw transmembrane rear-to-front gradient of GT46-GFP (an inert transmembrane chimera) in large blebs in three different cell lines – A549 (lung cancer cell line), U2OS (human osteosarcoma cell line) and A375P (melanoma cell line) formed under 3 micron confinement in low adhesive environments support of this phenomenon that these phenotypes are generalizable to other cell lines as well.

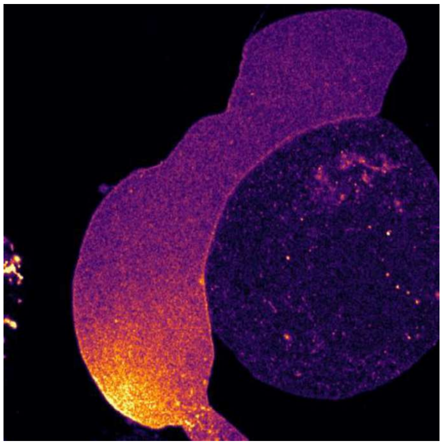

A549 lung cancer

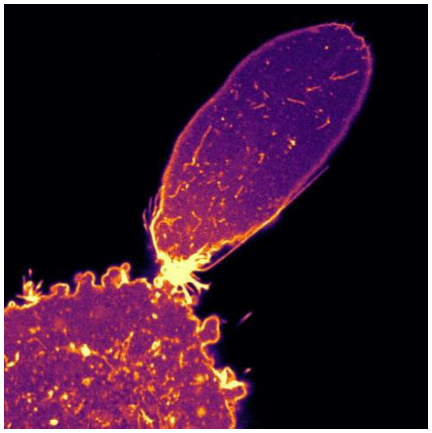

U2OS Osteosarcoma cell

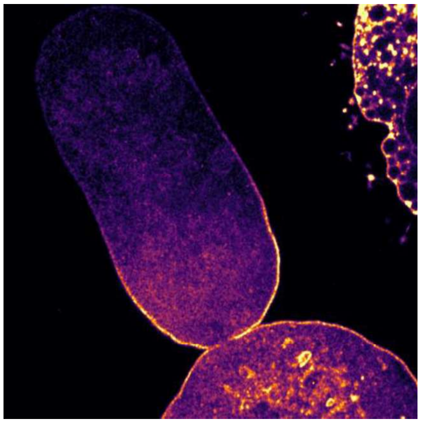

A375P melanoma cell line

Finally, the manuscript would benefit from additional discussion of the role of bleb-based migration in melanoma and melanoma metastasis, and how this work advances that field in general (ie Do melanomas largely use bleb-based amoeboid migration? What other tumors do?). Also a discussion of how this work meshes with earlier studies of the reverse polarity of these same melanoma cells (Lorentzen et al 2011).

We apologize to reviewer for not been able to add this in the Discussion section in the manuscript due to space constraints and indeed agree these are important points. However these have been discussed in details in recent reviews especially in (Graziani et al. 2022; García-Arcos, Jha, et al. 2024) which are cited in the manuscript. However we have modified the discussions for the clarity as follow:

"The spontaneous polarization of metastatic melanoma cells in the absence of chemical or haptotactic gradients, coupled with their efficient migration in confined, low-adhesion environments, has important implications for tumor invasion and metastasis[5,69,74]. Previous studies have shown high Erk signaling in

melanoma cell blebs, likely due to localized, restricted signaling[19]. High contractility can increase the invasive potential of cancer cells, confer anoikis resistance [75,76], and promote drug resistance [69]. How common these phenomena are across different solid tumors and how they change with disease progression remain important areas for further exploration."

References

Adams, Gregory, Magdalena Preciado López, Alexander X. Cartagena-Rivera, and Clare M. Waterman. 2021. "Survey of Cancer Cell Anatomy in Nonadhesive Confinement Reveals a Role for Filamin-A and Fascin-1 in Leader Bleb–Based Migration." *Molecular Biology of the Cell* 32 (18): 1772–91. https://doi.org/10.1091/mbc.E21-04-0174.

Aoki, Kana, Fumiyo Maeda, Tomoya Nagasako, Yuki Mochizuki, Seiichi Uchida, and Junichi Ikenouchi. 2016. "A RhoA and Rnd3 Cycle Regulates Actin Reassembly during Membrane Blebbing." *Proceedings of the National Academy of Sciences* 113 (13): E1863–71. https://doi.org/10.1073/pnas.1600968113.

Budnar, Srikanth, Kabir B. Husain, Guillermo A. Gomez, et al. 2019. "Anillin Promotes Cell Contractility by Cyclic Resetting of RhoA Residence Kinetics." *Developmental Cell* 49 (6): 894-906.e12. https://doi.org/10.1016/j.devcel.2019.04.031.

Cantley, Lewis C, Kurt R Auger, Christopher Carpenter, et al. 1991. "Oncogenes and Signal Transduction." *Cell* 64 (2): 281–302. https://doi.org/10.1016/0092-8674(91)90639-G.

De Belly, Henry, Andreu Fernandez Gallen, Evelyn Strickland, et al. 2024. "Long Range Mutual Activation Establishes Rho and Rac Polarity during Cell Migration." Preprint, Cell Biology, October 2. https://doi.org/10.1101/2024.10.01.616161.

Driscoll, Meghan K., Erik S. Welf, Andrew R. Jamieson, et al. 2019. "Robust and Automated Detection of Subcellular Morphological Motifs in 3D Microscopy Images." *Nature Methods* 16 (10): 1037–44. https://doi.org/10.1038/s41592-019-0539-z.

Driscoll, Meghan K., Erik S. Welf, Andrew Weems, et al. 2024. "Proteolysis-Free Amoeboid Migration of Melanoma Cells through Crowded Environments via Bleb-Driven Worrying." *Developmental Cell* 59 (18): 2414-2428.e8. https://doi.org/10.1016/j.devcel.2024.05.024.

Ehret, Thomas, Tim Heißenberg, Svenja De Buhr, Camilo Aponte-Santamaría, Claudia Steinem, and Frauke Gräter. 2023. "FERM Domains Recruit Ample PI(4,5)P2s to Form Extensive Protein-Membrane Attachments." *Biophysical Journal* 122 (7): 1325–33. https://doi.org/10.1016/j.bpj.2023.02.027.

Fanfone, Deborah, Zhichong Wu, Jade Mammi, et al. 2022. "Confined Migration Promotes Cancer Metastasis through Resistance to Anoikis and Increased Invasiveness." *eLife* 11 (March): e73150. https://doi.org/10.7554/eLife.73150.

Fantl, Wendy J., Jaime A. Escobedo, George A. Martin, et al. 1992. "Distinct Phosphotyrosines on a Growth Factor Receptor Bind to Specific Molecules That Mediate Different Signaling Pathways." *Cell* 69 (3): 413–23. https://doi.org/10.1016/0092-8674(92)90444-H.

Farahani, Payam E., Sandra B. Lemke, Elliot Dine, Giselle Uribe, Jared E. Toettcher, and Celeste M. Nelson. 2021. “Substratum Stiffness Regulates Erk Signaling Dynamics through Receptor-Level Control.” *Cell Reports* 37 (13): 110181. https://doi.org/10.1016/j.celrep.2021.110181.

Freeman, Spencer A., Anthony Vega, Magdalena Riedl, et al. 2018. “Transmembrane Pickets Connect Cyto- and Pericellular Skeletons Forming Barriers to Receptor Engagement.” *Cell* 172 (1–2): 305-317.e10. https://doi.org/10.1016/j.cell.2017.12.023.

García-Arcos, Juan Manuel, Ankita Jha, Clare M. Waterman, and Matthieu Piel. 2024. “Blebology: Principles of Bleb-Based Migration.” *Trends in Cell Biology* 34 (10): 838–53. https://doi.org/10.1016/j.tcb.2024.02.009.

García-Arcos, Juan Manuel, Johannes Ziegler, Silvia Grigolon, et al. 2024. “Rigidity Percolation and Active Advection Synergize in the Actomyosin Cortex to Drive Amoeboid Cell Motility.” *Developmental Cell*, July, S1534580724004015. https://doi.org/10.1016/j.devcel.2024.06.023.

Gerosa, Luca, Christopher Chidley, Fabian Fröhlich, et al. 2020. “Receptor-Driven ERK Pulses Reconfigure MAPK Signaling and Enable Persistence of Drug-Adapted BRAF-Mutant Melanoma Cells.” *Cell Systems* 11 (5): 478-494.e9. https://doi.org/10.1016/j.cels.2020.10.002.

Gorelik, Roman, and Alexis Gautreau. 2014. “Quantitative and Unbiased Analysis of Directional Persistence in Cell Migration.” *Nature Protocols* 9 (8): 1931–43. https://doi.org/10.1038/nprot.2014.131.

Graziani, Vittoria, Irene Rodriguez-Hernandez, Oscar Maiques, and Victoria Sanz-Moreno. 2022. “The Amoeboid State as Part of the Epithelial-to-Mesenchymal Transition Programme.” *Trends in Cell Biology* 32 (3): 228–42. https://doi.org/10.1016/j.tcb.2021.10.004.

Kim, Do-Hyeon, Hong Minh Triet, and Sung Ho Ryu. 2021. “Regulation of EGFR Activation and Signaling by Lipids on the Plasma Membrane.” *Progress in Lipid Research* 83 (July): 101115. https://doi.org/10.1016/j.plipres.2021.101115.

Liu, Yan-Jun, Maël Le Berre, Franziska Lautenschlaeger, et al. 2015. “Confinement and Low Adhesion Induce Fast Amoeboid Migration of Slow Mesenchymal Cells.” *Cell* 160 (4): 659–72. https://doi.org/10.1016/j.cell.2015.01.007.

Logue, Jeremy S, Alexander X Cartagena-Rivera, Michelle A Baird, Michael W Davidson, Richard S Chadwick, and Clare M Waterman. 2015. “Erk Regulation of Actin Capping and Bundling by Eps8 Promotes Cortex Tension and Leader Bleb-Based Migration.” *eLife* 4 (July): e08314. https://doi.org/10.7554/eLife.08314.

Lomakin, A. J., C. J. Cattin, D. Cuvelier, et al. 2020. “The Nucleus Acts as a Ruler Tailoring Cell Responses to Spatial Constraints.” *Science* 370 (6514): eaba2894. https://doi.org/10.1126/science.aba2894.

Ludwig, Megan L., Nicole L. Michmerhuizen, Jiayu Wang, et al. 2023. “Multi-Kinase Compensation Rescues EGFR Knockout in a Cell Line Model of Head and Neck Squamous Cell Carcinoma.” *Archives of Oral Biology* 156 (December): 105822. https://doi.org/10.1016/j.archoralbio.2023.105822.

Maiuri, Paolo, Jean-François Rupprecht, Stefan Wieser, et al. 2015. “Actin Flows Mediate a Universal Coupling between Cell Speed and Cell Persistence.” *Cell* 161 (2): 374–86. https://doi.org/10.1016/j.cell.2015.01.056.

Maiuri, Paolo, Emmanuel Terriac, Perrine Paul-Gilloteaux, et al. 2012. “The First World Cell Race.” *Current Biology* 22 (17): R673–75. https://doi.org/10.1016/j.cub.2012.07.052.

Orgaz, Jose L., Pahini Pandya, Rimple Dalmeida, et al. 2014. “Diverse Matrix Metalloproteinase Functions Regulate Cancer Amoeboid Migration.” *Nature Communications* 5 (1): 4255. https://doi.org/10.1038/ncomms5255.

Orofiamma, Laura A., Dafne Vural, and Costin N. Antonescu. 2022. “Control of Cell Metabolism by the Epidermal Growth Factor Receptor.” *Biochimica et Biophysica Acta (BBA) - Molecular Cell Research* 1869 (12): 119359. https://doi.org/10.1016/j.bbamcr.2022.119359.

Rei, Karin, Catherine D. Nobes, George Thomas, Alan Hall, and Doreen A. Cantrell. 1996. “Phosphatidylinositol 3-Kinase Signals Activate a Selective Subset of Rac/Rho-Dependent Effector Pathways.” *Current Biology* 6 (11): 1445–55. https://doi.org/10.1016/S0960-9822(96)00749-X.

Ruprecht, Verena, Stefan Wieser, Andrew Callan-Jones, et al. 2015. “Cortical Contractility Triggers a Stochastic Switch to Fast Amoeboid Cell Motility.” *Cell* 160 (4): 673–85. https://doi.org/10.1016/j.cell.2015.01.008.

Stokes, Cynthia L., Douglas A. Lauffenburger, and Stuart K. Williams. 1991. “Migration of Individual Microvessel Endothelial Cells: Stochastic Model and Parameter Measurement.” *Journal of Cell Science* 99 (2): 419–30. https://doi.org/10.1242/jcs.99.2.419.

Venturini, Valeria, Fabio Pezzano, Frederic Català Castro, et al. 2020. “The Nucleus Measures Shape Changes for Cellular Proprioception to Control Dynamic Cell Behavior.” *Science* 370 (6514): eaba2644. https://doi.org/10.1126/science.aba2644.

Weems, Andrew D., Erik S. Welf, Meghan K. Driscoll, et al. 2023. “Blebs Promote Cell Survival by Assembling Oncogenic Signalling Hubs.” *Nature* 615 (7952): 517–25. https://doi.org/10.1038/s41586-023-05758-6.

Weigelin, Bettina, Gert-Jan Bakker, and Peter Friedl. 2012. “Intravital Third Harmonic Generation Microscopy of Collective Melanoma Cell Invasion: Principles of Interface Guidance and Microvesicle Dynamics.” *IntraVital* 1 (1): 32–43. https://doi.org/10.4161/intv.21223.

Reviewer #1:
Remarks to the Author:
Jha et al. present an elegant and strong publication where they show that the same polarity machinery used in lamellipodia/mesenchymal migration can be repurposed and used to drive bleb-based migration under confinement. Their experimental work has a strong solid foundation that is supported by modelling. In the initial submission, we did not have strong concerns with the mechanism nor the evidence they provide for their work. In the response to the reviewer comments, the authors have tried to address to the best of their abilities and technical capabilities all the comments not only from our reviews, but from most of the reviewer's comments. All and all, we are happy with the current state of the paper and believe this would be a great addition for NCB.

We thank the reviewer for spending time for such detailed report of the manuscript.

Jha et al have done a great effort to revised their manuscript and completed most (if not all) the necessary changes that the reviewers asked. With that said some final minor comments to take into account:

Comment 1: Strong proof of EGFR direct effect in bleb-based migration
The authors corrected the negative migration persistence from the original plots. The original concern over EGFR function was that only pharmacological inhibitions showed a strong reduction in the migration phenotype of confined cells. These treatments lead to off-targets effects that may interfere with migration independent of EGFR. The authors have strengthened their proof of EGFR and its effect in bleb-based migration by adding an independent siRNA that shows a stronger effect. Along with these experiments, western blots show the protein depletion of EGFR for siRNA efficiency.
The authors have re-plotted, and in line with our comments and other reviewers, have added population level speeds using cumulative frequency and total mean squared displacement, strengthening the results and supporting the claim that EGFR drives bleb-based migration in confined cells.
Furthermore, we acknowledge it is not a trivial task to generate KO of EGFR and the experimental limitations this brings. Therefore, the current figure has strengthened the proof that EGFR and Pi3K pathways influence bleb-based migration in confinement and we feel this satisfies our initial concerns.

We thank the reviewer for their understanding.

Comment 2: Model strength
The authors have further explained the use of the model and how it helped them identify that retrograde actin flow cannot explain the existence of this EGFR gradient. The initial model predicted that changing actin flows (as in Fig Ext 2 K) would affect the gradient, but upon experimental perturbations this did not change the gradient of EGFR, meaning this was independent of actin retrograde flow. Later on, the model showed that if the actin retrograde flow was left constant, the

change in corralling would change the EGFR gradient (as in Fig 5 A).
The authors have now added a more detail explanation of the mathematical model importance and strengthened their evidence on corralling as the driver of the EGFR gradient with both experimental and modelling approaches. We feel the current text explanations and changes strengthened the model use and is now clearer why it was needed.

We thank the reviewer.

Comment 3: 3D bleb migration gradient proof lacking
The authors have raised concerns regarding the technical complexity of performing 3D imaging experiments for the proof that the current 2D phenomena can be interpolated to 3D migration. We acknowledge a full characterization of Rho/Rac sensors is technically challenging. Nevertheless, the current figure (5N) does not fully show the gradient observed in 2D, there is no quantification and the sample size is a single blebbing cell in a 3D ECM. Furthermore, the image is a single slice that lacks any volumetric information regarding the 3D distribution of the stained proteins. Since the authors claim this phenomenon should be explored in 3D in a different publication work, we recommend strengthen the discussion with further evidence (earlier referenced) where upstream/downstream biochemical molecules are explored.

We thank the reviewer for the suggestion. We have now added this in the discussions and it reads as

"Our findings suggest that during bleb based migration, CD44's primary role is biophysical, acting as a transmembrane "picket", to corral EGFR via an ERM protein and actin meshwork at the bleb rear, where lower receptor density at the front limits activation [72,73]. This configuration could form a feedback loop as growth factor signaling promotes downstream ERM protein phosphorylation, linking CD44 to actin. Although hyaluronan degradation didn't alter EGFR polarity in our experiments, this mechanism might vary in the tumor microenvironment. Moreover, our preliminary data show EGFR and CD44 gradient in the large bleb in 3D under more physiological conditions. Recent work has shown that this pathway is conserved in 3D, where upstream PI3K and Rac activity is restricted to bleb base along with other potential picket proteins like septin[70,74]. This suggests that CD44 restricts EGFR diffusion across the bleb, impacting its spatial distribution and contributing to polarized signaling."

Comment 4: Gradients of CD44-Ezrin in blebs and differences in gradient
We appreciate that the authors have modified their analysis and quantified through a normalization that better shows the image represented. In addition, the authors have compared the localization of p-ERMs compared to actin within the bleb. This comparison has now clarified that the differences on gradient between CD44 and ERMs arise from the un-specificity of labelling all Ezrin forms (active and inactive). The pERM staining clarifies the increase localization in the bleb bottom where most actin localizes.
As a technical comment, I do not understand why the authors did not compare the pERM to CDC44 or EGFR in the same cell and the sample size seems to be of a single blebbing cell.
Even though the sample size for this work is small, the addition of the p-ERM clarifies the earlier concerns we had and further reiterates how important it is to compare active ERMs compared to its bulk. We are satisfied with the new data.

We understand reviewers' concerns. The comparison of pERM to CD44 and EGFR was not performed together because our working antibodies could cross react because EGFR and pERM were raised in the same organism species. We currently have a low number of sample size because it is hard to tell the stage of blebbing (extension vs retraction) in immunostaining. Hence averaging across many blebs could actually lose the information.

Comment 5: Loss of EGFR/ Rac and CD44 gradients with optogenetic inhibition is affecting more persistence than speed. This comment seems to be a concerned shared across all reviewers so it was important to address it. The authors have now clarified the discrepancies in these results. Now, there are figures to compare population level changes using MSD, frequency and distribution of phenotypes. And specifically look at the speed and persistence of cells that successfully form stable blebs. Now this allows us to compare the population and migration level differences. This has changed across the paper and we greatly appreciate the clarity that this brings for the experiment. Since there are no significant differences in the mean speed but as mentioned Maiuri et al 2012, the distribution of the speed is reduced upon the different treatments, we recommend the authors to state this in the main text since very clearly even though the mean significance does not change, distribution seem to be a better metric to compare the changes in speed.

We thank the reviewer for this suggestion, we have added this in the beginning of the results in the main text and it reads as follows-

"Indeed, plotting the lifetime of elongated blebs vs either their migration persistence or diffusion coefficient showed positive correlations (Figure 1E, Extended Figure 1C). In contrast, similar analysis of bleb lifetime vs speed showed that cells with an elongated bleb moved at a wide range of speeds, independent of bleb lifetime (Extended Figure 1D, E). This is consistent with the previous studies which shows that cells move with a wide range of speed. Hence distribution of speed is a better metric for the changes in the speed compared to the mean speed[30]. These results show that stabilization and maintenance of a polarized leader bleb morphology is critical to efficient and persistent migration in metastatic melanoma cells in low-adhesive confinement."

Reviewer #2:
Remarks to the Author:
The authors have largely addressed my comments. While I appreciate the response in the rebuttal to the comment about how normally cells in confinement still have access to ligands in the ECM, I really think this should be included in the discussion section of the manuscript. It provides important context for these results and extends their relevance beyond the artificial low-adhesive environment used in this work.

Patrick Oakes

Remarks on Protocol(s):
The troubleshooting section of the protocol is empty. It would be useful to provide more details in this section about potential issues that can come up.

We thank the reviewer for all the wonderful suggestions, with limited space we have added this in the discussions and it currently read as

“The spontaneous polarization of metastatic melanoma cells in the absence of chemical or haptotactic gradients, coupled with their efficient migration in confined, low-adhesion environments, has important implications for tumor invasion and metastasis. Even in complex environments with ECM ligands present, cancer cells navigating high confined spaces are able to switch to pressure based motility[5,69,74]. Melanoma cells have high ERK and MAPK signaling due to BRAF mutation conferring high contractility that increases the invasive potential of cancer cells, confer anoikis resistance [75,76], and promote drug resistance [69] possibly by EGFR signaling at the back of the bleb. What is the impact of confinement on these signaling pathways and how common these phenomena are across different solid tumors and how they change with disease progression remain important areas for further exploration.”

Reviewer #3:
Remarks to the Author:
Jha and colleagues have done a nice job of revising this manuscript per my comments. I still think they should better discuss the likely much more complex nature of signaling that drives this fascinating and potentially clinically important phenotype (and missed a bit of an opportunity to bring a wider audience to this important fundamental science). These melanoma cells have a constitutively active Raf mutation and therefore abnormally high levels of MAPK signaling and likely well-known negative feedback - especially given their previous work on the role of ERK in bleb-based migration in these cells. But I will not stand in the way of publishing this otherwise lovely work.

We thank the reviewer for this suggestion. It is indeed a very interesting point and with limited space we have discussed this in the discussions and it reads as

“The spontaneous polarization of metastatic melanoma cells in the absence of chemical or haptotactic gradients, coupled with their efficient migration in confined, low-adhesion environments, has important implications for tumor invasion and metastasis. Even in complex environments with ECM ligands present, cancer cells navigating high confined spaces are able to switch to pressure based motility[5,69,74]. Melanoma cells have high ERK and MAPK signaling due to BRAF mutation conferring high contractility that increases the invasive potential of cancer cells, confer anoikis resistance [75,76], and promote drug resistance [69] possibly by EGFR signaling at the back of the bleb. What is the impact of confinement on these signaling pathways and how common these phenomena are across different solid tumors and how they change with disease progression remain important areas for further exploration.”
